# Stereoconvergent and Chemoenzymatic Synthesis of Tumor-Associated Glycolipid Disialosyl Globopentaosylceramide for Probing the Binding Affinity of Siglec-7

**DOI:** 10.1021/acscentsci.3c01170

**Published:** 2024-01-24

**Authors:** Yating Liu, Mengkun Yan, Minghui Wang, Shiwei Luo, Shasha Wang, Yawen Luo, Zhuojia Xu, Wenjing Ma, Liuqing Wen, Tiehai Li

**Affiliations:** † State Key Laboratory of Chemical Biology, Shanghai Institute of Materia Medica, Chinese Academy of Sciences, Shanghai 201203, China; ‡ School of Chinese Materia Medica, Nanjing University of Chinese Medicine, Nanjing 210023, China; § University of Chinese Academy of Sciences, Beijing 100049, China

## Abstract

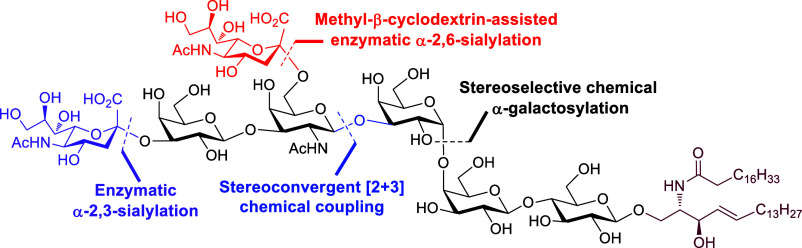

Disialosyl globopentaosylceramide (DSGb5) is a tumor-associated complex glycosphingolipid. However, the accessibility of structurally well-defined DSGb5 for precise biological functional studies remains challenging. Herein, we describe the first total synthesis of DSGb5 glycolipid by an efficient chemoenzymatic approach. A Gb5 pentasaccharide-sphingosine was chemically synthesized by a convergent and stereocontrolled [2 + 3] method using an oxazoline disaccharide donor to exclusively form β-anomeric linkage. After investigating the substrate specificity of different sialyltransferases, regio- and stereoselective installment of two sialic acids was achieved by two sequential enzyme-catalyzed reactions using α2,3-sialyltransferase Cst-I and α2,6-sialyltransferase ST6GalNAc5. A unique aspect of the approach is that methyl-β-cyclodextrin-assisted enzymatic α2,6-sialylation of glycolipid substrate enables installment of the challenging internal α2,6-linked sialoside to synthesize DSGb5 glycosphingolipid. Surface plasmon resonance studies indicate that DSGb5 glycolipid exhibits better binding affinity for Siglec-7 than the oligosaccharide moiety of DSGb5. The binding results suggest that the ceramide moiety of DSGb5 facilitates its binding by presenting multivalent interactions of glycan epitope for the recognition of Siglec-7.

## Abstract 2

Efficient chemoenzymatic synthesis of tumor-associated disialosyl globopentaosylceramide (DSGb5) glycolipid has been accomplished, and molecular insights into its binding with Siglec-7 are revealed.

## Introduction

**Figure 1:**
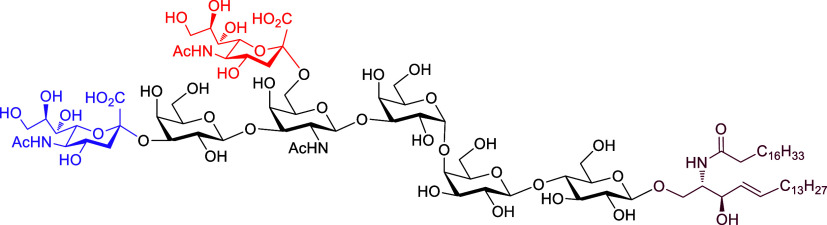
Chemical structure of disialosyl globopentaosylceramide (DSGb5).

Glycosphingolipids, amphipathic biomolecules composed of hydrophilic carbohydrate chains attached to hydrophobic ceramide lipid chains, are essential components of vertebrate cell membranes and involved in various biological activities such as cell adhesion, signal transduction, immune modulation, virus infection, and cancer proliferation and metastasis.^1−3^ Gangliosides are sialic acid-containing complex glycosphingolipids, which are recognized as functional ligands for sialic acid-binding immunoglobulin-like lectins (Siglecs) that are involved in regulation of immune cell functions in disease.^4−6^ Disialosyl globopentaosylceramide (DSGb5) was identified as a major ganglioside from renal cell carcinoma (RCC) tissue extracts.^7^ DSGb5 possesses a unique globopentaosylceramide structure (Galβ1,3GalNAcβ1,4Galα1,4Galβ1,4Glcβ-ceramide) with an α2,3-Neu5Ac at the terminal Gal and an α2,6-Neu5Ac at the internal GalNAc moiety (Figure 1). Overexpression of ganglioside DSGb5 has been found in aggressive RCC cells and promotes the migration of RCC cells.^8^ Moreover, abnormal DSGb5 expression may be related to prostate cancer deterioration.^9^ Variations in ganglioside expression level influence the interactions of gangliosides with Siglecs and result in human pathology such as tumor cell proliferation and metastasis.^4−6^ Recent cell-based studies have also indicated that ganglioside DSGb5 serves as a ligand of sialic acid-binding immunoglobulin-like lectin 7 (Siglec-7) expressed on natural killer cells (NKC), thus inhibiting the NKC cytotoxicity in a DSGb5-Siglec-7-dependent manner to facilitate the survival and metastasis of cancer cells.^10^ Surprisingly, Lin’s work^11^ and our previous work^12^ demonstrated that the glycan structure of DSGb5 showed low or no binding affinity for Siglec-7 by glycan microarray analysis. Given recent evidence that the ceramide structure of disialoganglioside GD3 is essential for its recognition by Siglec-7 on the cell surface,^13^ the binding of DSGb5 and Siglec-7 may be realized by the synergistic effects of both the oligosaccharide moiety and the ceramide chain. Therefore, the structurally well-defined DSGb5 glycosphingo-lipid is in urgent demand, which could be used to investigate its binding with Siglec-7 and other biological functions.

**Scheme 1:**
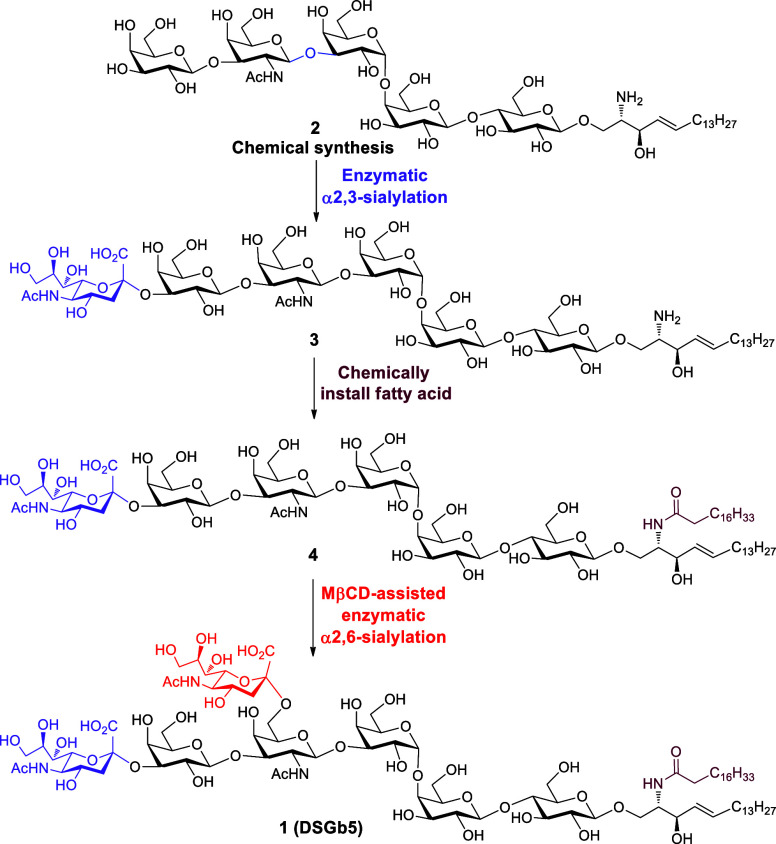
Chemoenzymatic Approach for Synthesis of DSGb5 Glycosphingolipid MβCD = methyl-β-cyclodextrin, DSGb5 = disialosyl globopentaosylceramide.

The microheterogeneity and structural complexity of glycosphingolipids^2,14^ make it difficult or even impossible to acquire structurally well-defined DSGb5 isolated from cancer cells or tissues in high purity with sufficient quantities, which severely restricts its study at the molecular level. Alternatively, chemical and chemoenzymatic approaches provide access to the synthesis of homogeneous and well-defined glycolipids.^15−27^ Due to the exceeding complexity of DSGb5 in structure (Figure 1), the critical challenging aspects of the synthesis of DSGb5 include the following: (1) stereo- and regioselective installment of α2,3- and α2,6-linked sialic acids^28,29^ in optimal sequence for the assembly of the oligosaccharide chain, (2) appropriate incorporation of sphingosine and the fatty acid of ceramide into the oligosaccharide moiety. Previously, great efforts were only dedicated to the synthesis of the oligosaccharide moiety of DSGb5.^11,12,30^ Nevertheless, there is still no approach to synthesize the whole DSGb5 glycosphingolipid. Herein, we report the first total synthesis of DSGb5 glycolipid by an efficient chemoenzymatic approach (Scheme 1). A unique feature of the method is that convergent access to Gb5 pentasaccharide sphingosine is efficiently assembled by a stereocontrolled [2 + 3] chemical glycosylation using an oxazoline disaccharide donor to exclusively form a β-anomeric linkage. More importantly, based on our detailed investigation of the substrate specificity of sialyltransferases, regio- and stereoselective enzymatic installment of two sialic acids was achieved by a bacterial α2,3-sialyltransferase Cst-I that can well recognize Gb5-sphingosine as the substrate and a mammalian α2,6-sialyltransferase ST6GalNAc5 that can recognize α2,3-sialylated Gb5-ceramide as the substrate with the assistance of methyl-β-cyclodextrin (MβCD) to successfully provide DSGb5 glycosphingolipid. Additionally, surface plasmon resonance assays were applied to probe the binding affinity of Siglec-7 with DSGb5 glycolipid, the oligosaccharide moiety of DSGb5, the monosialyl Gb5 (MSGb5) glycolipid, and the nonsialylated Gb5 glycolipid for investigating their structure–binding relationships.

## Results and Discussion

### Chemical Synthesis of Gb5-Sphingosine **2**

**Scheme 2:**
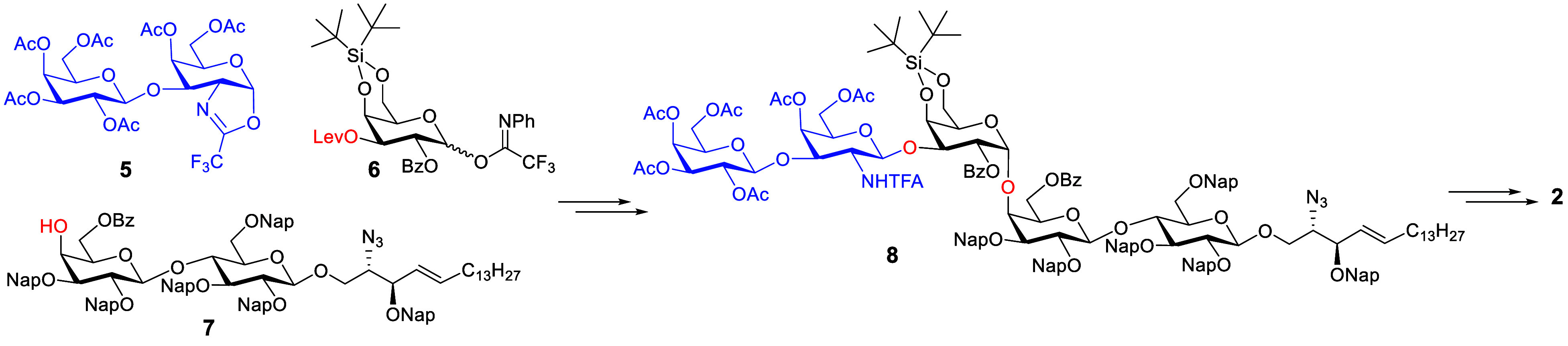
Building Blocks for Chemical Synthesis of Gb5-Sphingosine **2** by a Stereoconvergent Approach Ac = acetyl, Lev = levulinoyl, Bz = benzoyl, Ph = phenyl, Nap = 2-naphthylmethyl, TFA = trifluoroacetyl.

Despite innovative strategies and methodologies having been reported to prepare only the oligosaccharide moiety of Gb5-sphingosine,^31−35^ these methods require either the tedious separation of stereoisomers with low yields in chemical synthesis or large amounts of enzymes with low conversions and unwanted byproducts such as β1,3-Gal-Gb5 in enzymatic synthesis. To address the above problems, it was envisaged that the protected Gb5-sphingosine **8** could be efficiently prepared by a stereoconvergent [2 + 3] method (Scheme 2) that relies on the usage of disaccharide oxazoline donor **5**, which would result in absolute β-anomeric selectivity.^36−38^ The bulky 4,6-di-*O*-*tert*-butylsilylene (DTBS)^39−41^ protecting group of glycosyl donor **6** could prevent the β-face attack of compound **7** for stereoselective installment of the challenging α(1 → 4)-linked Gal–Gal to afford a protected trisaccharide, which would be readily converted to a glycosyl acceptor by selectively removing levulinoyl (Lev) ester with hydrazine acetate.^42,43^ Additionally, 2-naphthylmethyl (Nap) as a permanent protecting group would be selectively cleaved with 2,3-dichloro-5,6-dicyano-1,4-benzoquinone (DDQ)^44,45^ to release a hydroxyl group without affecting the double bond of the lipid moiety. Global deprotection of **8**, conversion of trifluoroacetyl (TFA) to acetyl (Ac), and reduction of azide (N_3_) to amine (NH_2_) would afford the desired compound **2**.

**Scheme 3:**
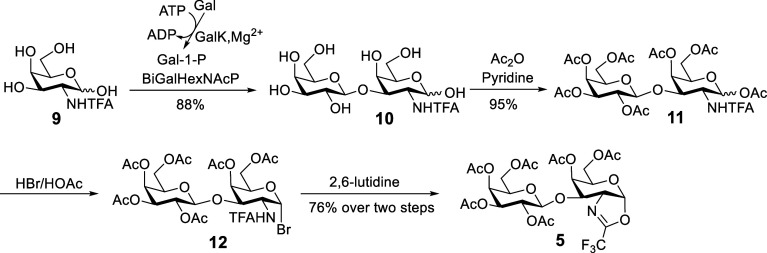
Chemoenzymatic Synthesis of Disaccharide Oxazoline Donor **5** Gal = galactose, ATP = adenosine 5′-triphosphate, ADP = adenosine 5′-diphosphate, GalK = galactokinase, Gal-1-P = galactose-1-phosphate, BiGalHexNAcP = *Bifidobacterium infantis*d-galactosyl-β1,3-*N*-acetyl-d-hexosamine phosphorylase, Ac_2_O = acetic anhydride, TFA = trifluoroacetyl, Ac = acetyl.

Based on the above plan for the preparation of Gb5-sphingosine **2**, the synthesis commenced with disaccharide oxazoline donor **5**, which was prepared by a facile chemoenzymatic protocol (Scheme 3). The commercially available *N*-trifluoroacetyl-d-galactosamine **9** as a starting material was subjected to an efficient one-pot two-enzyme reaction to afford disaccharide **10** by using *Escherichia coli* galactokinase (GalK) and *Bifidobacterium infantis*d-galactosyl-β1,3-*N*-acetyl-d-hexosamine phosphorylase (BiGalHexNAcP) in the presence of galactose (Gal) and ATP.^46^ The acetylation of **10** with an excess amount of acetic anhydride (Ac_2_O) provided per-acetylated disaccharide **11**, which was readily converted into bromide **12** using 33% HBr in acetic acid (HOAc). The resulting bromide was immediately treated with 2,6-lutidine to afford the desired oxazoline donor **5**.^47^

**Scheme 4:**
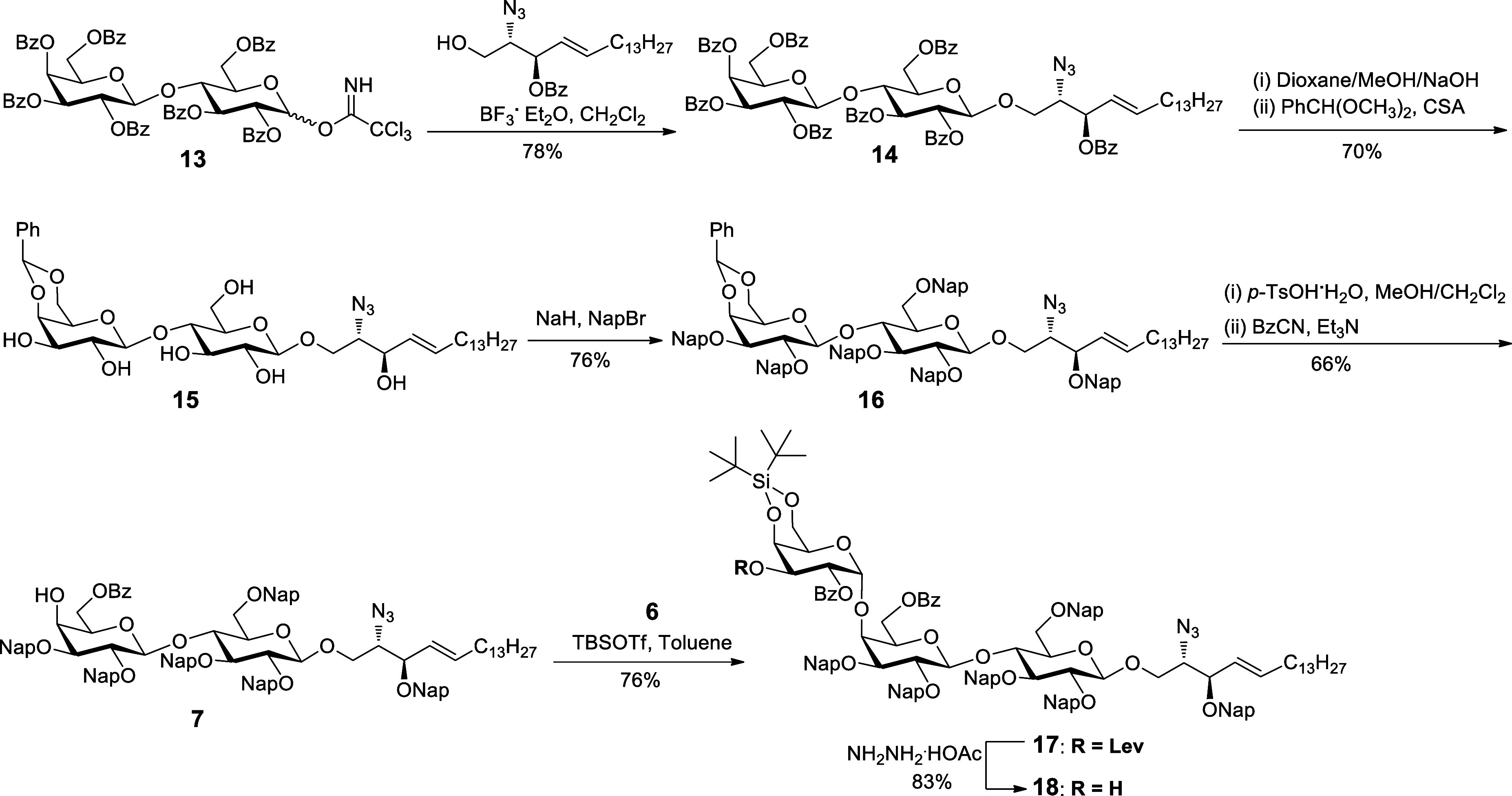
Synthesis of Trisaccharide Acceptor **18** PhCH(OCH_3_)_2_ = benzaldehyde dimethyl acetal, CSA = camphorsulfonic acid, NapBr = 2-naphthylmethyl bromide, *p*-TsOH·H_2_O = *p*-toluenesulfonic acid monohydrate, BzCN = benzoyl cyanide, TBSOTf = *tert*-butyldimethylsilyl trifluoromethanesulfonate, Bz = benzoyl, Ph = phenyl, Nap = 2-naphthylmethyl, Lev = levulinoyl.

Next, we turned our attention to synthesize trisaccharide acceptor **18** (Scheme 4). The coupling of readily accessible per-*O*-benzoyl lactosyl trichloroacetimidate donor **13**(48) with an azidosphingosine acceptor^22^ using BF_3_·OEt_2_ as an activator provided compound **14**. The saponification for removal of all benzoyl (Bz) groups to release hydroxyl groups using sodium hydroxide^49^ provided lactoside intermediate, which was treated with benzaldehyde dimethyl acetal (PhCH(OCH_3_)_2_) and camphorsulfonic acid (CSA) to afford compound **15**. To be compatible with a double bond at a later stage of deprotection, Nap protecting groups, which could be selectively cleaved by DDQ without affecting the olefinic bond of the lipid moiety, were chosen to mask all of the hydroxyl groups of **15** in the presence of NaH and 2-naphthylmethyl bromide (NapBr) to afford compound **16**. The cleavage of the 4,6-benzylidene acetal of **16** with *p*-toluenesulfonic acid monohydrate (*p*-TsOH·H_2_O) in a mixture solution of dichloromethane and methanol gave the corresponding 4,6-diols,^45^ which was treated with benzoyl cyanide (BzCN) and trimethylamine (Et_3_N) for selective benzoylation of the primary alcohol^50^ to afford glycosyl acceptor **7**. The glycosylation of *N*-phenyltrifluoroacetimidate donor **6** and disaccharide acceptor **7** using *tert*-butyldimethylsilyl trifluoromethanesulfonate (TBSOTf) as a promoter in toluene constructed smoothly an α(1 → 4)-linked Gal-Gal glycosidic linkage to provide the protected trisaccharide **17** as only the α-anomer (*J*_C1–H1_ = 171 Hz, Supporting Information p S45) in 76% yield.^51,52^ The successful glycosylation reaction is mainly attributed to increasing the reactivity of axial C4-hydroxyl with a Nap-ether-protected acceptor in comparison to a disarmed benzoyl-ester-protected acceptor with a low yield (Supporting Information Table S1) and bulky α-stereodirecting group DTBS of the donor.^39−41^ The treatment of **17** with hydrazine acetate to selectively remove the Lev protecting group afforded trisaccharide acceptor **18** in 83% yield.

**Scheme 5:**
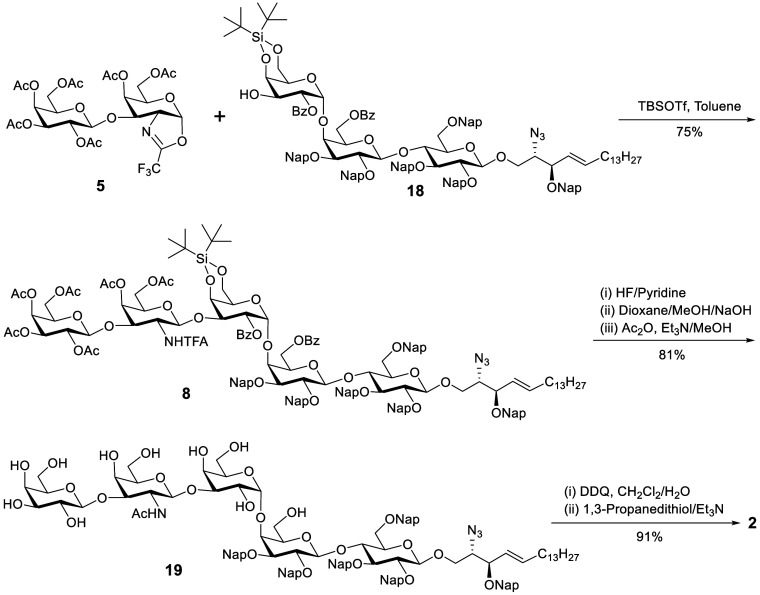
Synthesis of Gb5-Sphingosine **2** TBSOTf = *tert*-butyldimethylsilyl trifluoromethanesulfonate, Ac_2_O = acetic anhydride, DDQ = 2,3-dichloro-5,6-dicyano-1,4-benzoquinone, Ac = acetyl, Bz = benzoyl, Nap = 2-naphthylmethyl, TFA = trifluoroacetyl.

Having disaccharide oxazoline donor **5** and trisaccharide acceptor **18** in hand, attention was focused on synthesizing Gb5-sphingosine **2** (Scheme 5). The construction of the β(1 → 3)-linked GalNAc–Gal glycosidic linkage in **8** remains challenging because our previous work and Lin’s work indicated that disaccharide trichloroacetimidate and thioglycoside donors with neighboring group (2-NHTroc) participation for glycosylation irregularly afforded the pentasaccharide as a mixture of α/β anomers,^12,30^ which required the tedious separation of stereoisomers to give the desired β anomer with low yields. Gratifyingly, coupling of oxazoline donor **5** with acceptor **18** using TBSOTf as a promoter afforded pentasaccharide **8** as only the β anomer (*J*_C1–H1_ = 162 Hz, Supporting Information p S50) in 75% yield. Subsequently, the DTBS protecting group was removed in HF/pyridine, followed by the removal of Ac, Bz, and TFA protecting groups using aqueous NaOH in a mixture solution of dioxane and methanol to provide a partially deprotected intermediate.^41^ The resulting amino group of this intermediate was selectively acetylated with Ac_2_O in the presence of Et_3_N and MeOH to give compound **19**. Finally, Nap ethers of **19** were oxidatively cleaved to release hydroxyl groups by DDQ^44,45^ without affecting the double bond of the lipid moiety, followed by selectively reducing the azido group using 1,3-propanedithiol with Et_3_N^53^ to afford the desired Gb5-sphingosine **2**.

### Chemoenzymatic Synthesis of DSGb5 Glycolipid **1**

**Scheme 6:**
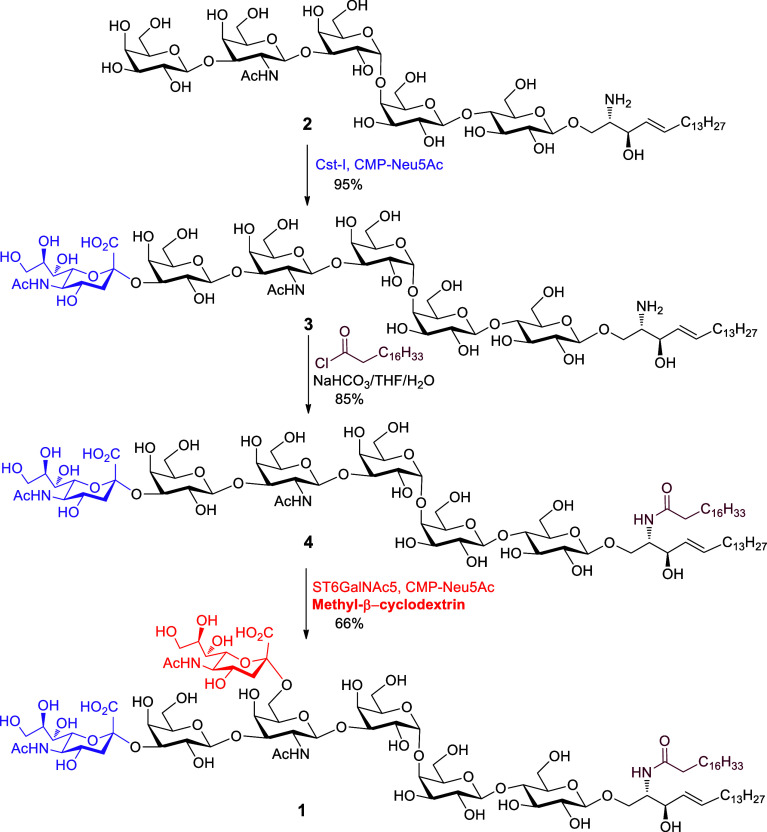
Synthesis of DSGb5 Glycosphingolipid **1** Cst-I = *Campylobacter jejuni* α2,3-sialyltransferase I, CMP-Neu5Ac = cytidine-5′-monophospho-*N*-acetylneuraminic acid, ST6GalNAc5 = ST6 *N*-acetylgalactosaminide α2,6-sialyltransferase 5.

Despite Gb5-sphingosine **2** having good solubility in aqueous buffer solution for enzymatic reaction, the regioselective enzymatic introduction of two sialic acids into **2** is challenging due to the narrow substrate specificity of sialyltransferases for glycolipids. A mammalian α2,3-sialyltransferase ST3Gal1 expressed from HEK293 cells can well recognize the Gb5 oligosaccharide moiety as a substrate to give monosialylated Gb5 hexasaccharide (SSEA-4 glycan, Supporting Information Scheme S5).^12^ However, using the same reaction conditions, the treatment of **2** with ST3Gal1 and CMP-Neu5Ac did not generate any product by electrospray ionization-mass spectrometry (ESI-MS) analysis. To overcome the problem, we tested two bacterial α2,3-sialyltransferases PmST1M144D^54^ and Cst-I^55^ expressed from *E. coli* for α2,3-sialylation of **2**. The results indicated that PmST1M144D-catalyzed α2,3-sialylation of **2** was slow, during which hydrolysis of a large amount of donor (CMP-Neu5Ac) was observed by ESI-MS analysis, thereby resulting in a low yield (Supporting Information Table S2). Gratifyingly, compound **2** was efficiently sialylated by Cst-I in the presence of CMP-Neu5Ac to afford SSEA-4 sphingosine **3** in a high yield of 95%. Furthermore, Cst-I-catalyzed α2,3-sialylation of **2** was very fast, which could be completed in 40 min (detailed procedure in the Supporting Information, p S23). Next, our attention was turned to the installment of an α2,6-linked sialic acid to the internal GalNAc moiety. A mammalian α2,6-sialyltransferase ST6GalNAc5 can readily transform the hexasaccharide moiety of SSEA-4 into disialosyl Gb5 heptasaccharide (DSGb5 glycan, Supporting Information Scheme S5).^12^ However, we found this enzyme cannot recognize compound **3** as the substrate for α2,6-sialylation. The previous acceptor substrate specificity showed that ST6GalNAc5 preferred sialylglycolipids containing the Neu5Acα2,3Galβ1,3GalNAc epitope as substrates such as ganglioside GM1b ceramide.^56^ Therefore, the sphingosine moiety of **3** was smoothly acetylated by stearoyl chloride in the presence of aqueous NaHCO_3_ and THF to afford monosialyl Gb5 ceramide **4** (MSGb5 glycolipid, Scheme 6). Subsequently, α2,6-sialylation of **4** with ST6GalNAc5 did not provide the desired target DSGb5. Probably the low solubility of **4** with ceramide in aqueous buffer solution resulted in the failure of sialylation. To solve this issue, it was envisaged that the usage of methyl-β-cyclodextrin for forming water-soluble inclusion complexes with an amphipathic compound would facilitate solubility enhancement.^57,58^ As expected, the treatment of **4** with ST6GalNAc5 and CMP-Neu5Ac in the presence of methyl-β-cyclodextrin successfully installed α2,6-sialoside at an internal GalNAc residue to afford the target **1** in 66% yield (detailed procedure in the Supporting Information, p S25). Consequently, the addition of methyl-β-cyclodextrin was essential for the α2,6-sialylation of **4**, which could improve the solubility of glycolipid in aqueous buffer solution for enzymatic reaction.^57,58^ Additionally, it should be noted that each enzymatic transformation can be easily analyzed by ESI-MS. If the starting glycolipid substrate remains in the enzyme-catalyzed reaction, additional enzymes and sugar nucleotides will be added until the homogeneous product is formed.

### Surface Plasmon Resonance Binding Assays

**Figure 2:**
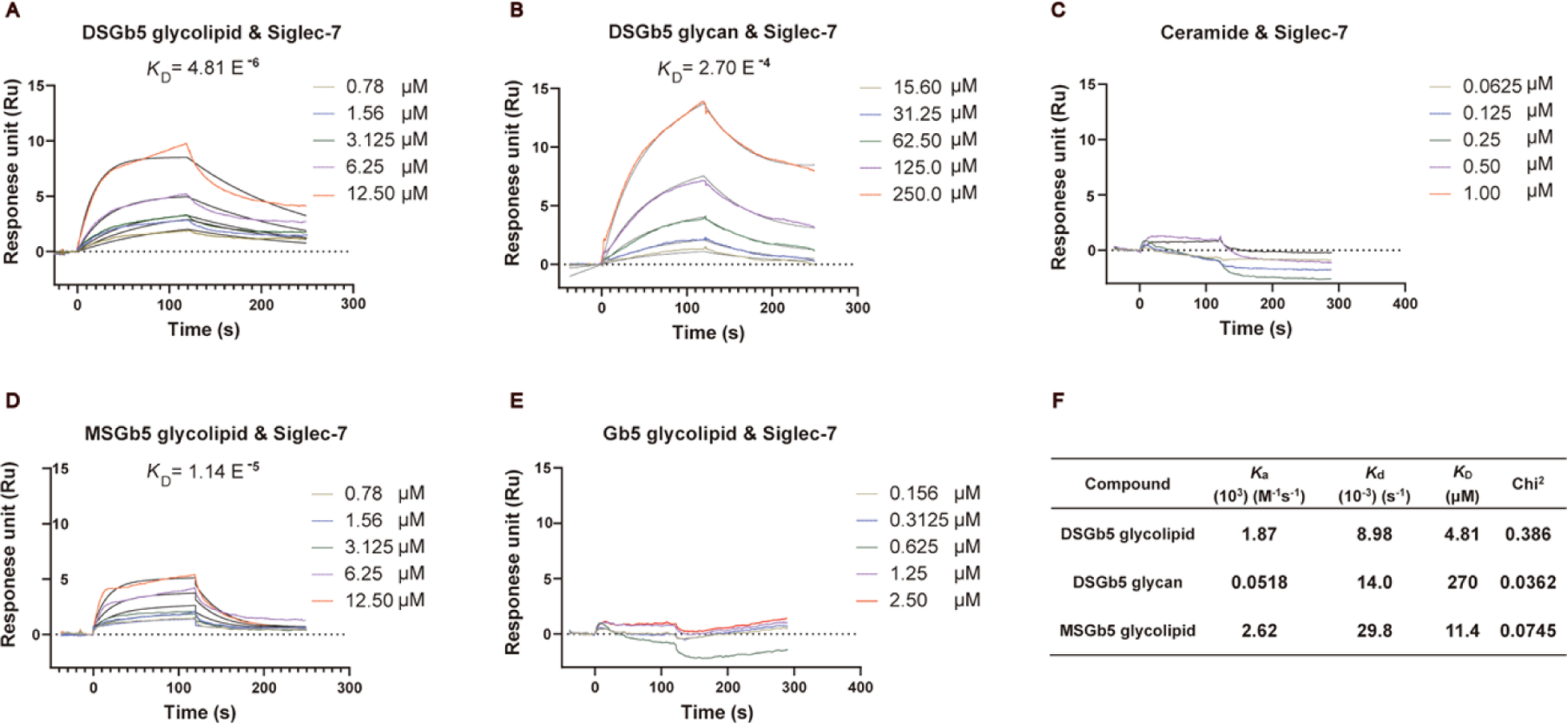
SPR analysis of the binding affinities of glycolipids, glycan, and ceramide with Siglec-7. Equilibrium dissociation constants (*K*_D_) were determined by global fitting of the binding data to a 1:1 Langmuir binding model. The black line is the fitting curve. (A) DSGb5 glycolipid, (B) DSGb5 glycan, (C) ceramide, (D) MSGb5 glycolipid, (E) Gb5 glycolipid. (F) Table of association rate constants (*K*_a_), dissociation rate constants (*K*_d_), *K*_D_, and chi-square (Chi^2^) goodness-of-fit values. *K*_D_ can be calculated as the ratio of *K*_d_ to *K*_a_.

Previous studies indicated that DSGb5 expressed on renal cancer cells could bind to Siglec-7 transfected on COS-7 cells.^10,59^ However, recent glycan microarray analysis demonstrated that the oligosaccharide moiety of DSGb5 showed low or no binding affinity for Siglec-7.^11,12^ We wonder whether the ceramide structure of DSGb5 can modulate its binding to Siglec-7. To address this issue, surface plasmon resonance (SPR) experiments were carried out to investigate the binding of Siglec-7 with DSGb5 glycolipid and DSGb5 glycan. Biotinylated Siglec-7 was immobilized on a streptavidin-coated sensor chip, and different concentrations of DSGb5 glycolipid and DSGb5 glycan as analytes were run to probe the binding affinities.^43,60^ As illustrated in Figure 2A–C, DSGb5 glycolipid exhibits better binding affinity (*K*_D_ = 4.81 E^–6^) for Siglec-7 than DSGb5 glycan (*K*_D_ = 2.70 E^–4^), whereas no obvious binding was observed for the ceramide with Siglec-7. Furthermore, DSGb5 glycolipid intermediates such as nonsialylated Gb5 glycolipid and monosialyl Gb5 (MSGb5) glycolipid were also prepared for exploration into the influence of the sialic acid residue for the binding of Siglec-7 by SPR assays (Figure 2D and 2E). The disialylated compound (DSGb5 glycolipid) displayed better binding affinity for Siglec-7 (*K*_D_ = 4.81 E^–6^) than monosialylated compound MSGb5 glycolipid (*K*_D_ = 1.14 E^–5^), whereas no obvious binding was observed for nonsialylated compound Gb5 glycolipid. This evidence indicates that the carbohydrate moieties, especially the degree of sialylation, are a key element for Siglec-7 recognition. Compared with DSGb5 glycan, the stronger binding generated by DSGb5 glycolipid is speculated to result from the ceramide-mediated cluster effect for presenting multivalent interactions of glycan epitope with Siglec-7,^13,61^ in a similar manner with multivalent glycan polymer Neu5Ac-α2,6GalNAc-α-PAA-biotin (a positive control ligand of Siglec-7, Supporting Information Figure S4A).^62,63^ Meanwhile, the effect of the ceramide structure has also been explored by Furukawa’s recent work that the introduction of a hydrophilic hydroxyl group to the ceramide moiety of disialoganglioside GD3 can lead to an eliminated binding affinity for its recognition by Siglec-7 on the cell surface, demonstrating that the existence and alteration of the ceramide structure may influence the carbohydrate epitope conformation and presentation for binding Siglec-7.^13,64^ Additionally, the quality of our SPR assays was validated by the commercially available positive control (Neu5Ac-α2,6GalNAc-α-PAA-biotin) and reported positive glycolipid control GD3 glycolipid for Siglec-7 (Supporting Information Figure S4A and 4B).^13,63^ The binding specificity of DSGb5 glycolipid was also demonstrated by its SPR assay with biotinylated Siglec-10, which showed no obvious binding for DSGb5 glycolipid (Supporting Information Figure S4C). The SPR binding results of our chemoenzymatically synthesized DSGb5 glycolipid demonstrate that the ceramide moiety of DSGb5 facilitates its binding with Siglec-7, suggesting that the ceramide chain may organize the glycolipid into microdomains to create cluster effects and multivalent interactions of the glycan epitope for the recognition of Siglec-7.^12,13,63−65^ Overall, all of the evidence suggests that the binding of DSGb5 and Siglec-7 is realized by the synergistic effects of both the glycan structure and the ceramide chain.

## Conclusion

In summary, the first total synthesis of complex DSGb5 glycosphingolipid has been accomplished via an efficient chemoenzymatic approach. A convergent strategy made it possible to streamline the assembly of Gb5 pentasaccharide sphingosine by the stereocontrolled chemical glycosylations using an oxazoline disaccharide donor and a bulky 4,6-di-*O*-*tert*-butylsilenyl (DTBS)-protected glycosyl donor. In particular, we employed 2-naphthylmethyl (Nap) ethers as permanent protecting groups, which could be selectively cleaved by DDQ without affecting the double bond of the lipid moiety, thus facilitating the final deprotection steps. Additionally, the stereo- and regioselective introduction of two sialic acids was achieved by appropriate sialyltransferases on the basis of our detailed investigation into substrate specificities of enzymes. Addition of methyl-β-cyclodextrin improved the low solubility of the glycolipid substrate for enzymatic reaction, thereby successfully installing the internal α2,6-linked sialoside to synthesize the target DSGb5 glycosphingolipid. Besides, DSGb5 glycolipid exhibited better binding affinity for Siglec-7 than the oligosaccharide moiety of DSGb5 by SPR analysis, indicating that the ceramide moiety of DSGb5 facilitated its binding by presenting multivalent interactions of the glycan epitope for the recognition of Siglec-7.^13,63^ Furthermore, SPR analysis also demonstrated that variations in the sialylation of our synthetic glycolipids showed different binding affinities with Siglec-7. These results suggested that the synergistic effects of both the glycan structure and the ceramide chain in DSGb5 glycolipid mediated effective binding with Siglec-7. This work expands the chemoenzymatic toolbox of glycolipid synthesis, which could be suitable to efficiently synthesize other complex glycosphingolipids for the development of glycolipid-based vaccines and therapeutic agents.

## Supporting Information Available

Experimental procedures, methods and compound characterization: glycosyltransferase expression, enzymatic reactions, chemical syntheses, SPR assays, additional schemes, figures, and tables, NMR and HRMS spectra (PDF)
