# Supplementary material for: Stereoconvergent and Chemoenzymatic Synthesis of Tumor-Associated Glycolipid Disialosyl Globopentaosylceramide for Probing the Binding Affinity of Siglec-7
Source: ACS Cent Sci. 2024 Jan 24;10(2):417–25. doi: 10.1021/acscentsci.3c01170 (PMC10906248; doi:10.1021/acscentsci.3c01170)
Supplement: Supplementary file 1 — oc3c01170_si_001.pdf [file oc3c01170_si_001.pdf]

Supporting Information

Stereoconvergent and Chemoenzymatic Synthesis of Tumor-Associated Glycolipid Disialosyl Globopentaosylceramide for Probing the Binding Affinity of Siglec-7

Yating Liu[a,b], Mengkun Yan[a,c], Minghui Wang[a], Shiwei Luo[a,b], Shasha Wang[a,b], Yawen Luo[a,c], Zhuojia Xu[a,c], Wenjing Ma[a,c], Liuqing Wen[a,b,c], and Tiehai Li[a,b,c]*

[a]*State Key Laboratory of Chemical Biology, Shanghai Institute of Materia Medica, Chinese Academy of Sciences, Shanghai 201203, China*

[b]*School of Chinese Materia Medica, Nanjing University of Chinese Medicine, Nanjing 210023, China*

[c]*University of Chinese Academy of Sciences, Beijing 100049, China*

*E-mail: tiehaili@simm.ac.cn

Table of Contents

1. General materials and methods ········ S1

2. Glycosyltransferase expression ········ S2

3. The labels of monosaccharide residues for NMR nomenclature ········ S4

4. Chemoenzymatic synthesis of disaccharide oxazoline donor **5** ········ S5

5. Synthesis of trisaccharide acceptor **18** ········ S8

6. Synthesis of Gb5-sphingosine **2** ········ S17

7. Synthesis of Gb5 glycolipid **20** ········ S21

8. Chemoenzymatic synthesis of DSGb5 glycolipid **1** ········ S22

9. Enzymatic synthesis of DSGb5 glycan ········ S26

10. Experimental procedure of surface plasmon resonance ········ S28

11. References ········ S29

12. NMR and HRMS spectra ········ S31

1. General materials and methods

Organic reactions were performed under an atmosphere of argon using anhydrous solvents unless otherwise noted. Proton nuclear magnetic resonance (^{1}H NMR) spectra were recorded on a Bruker Avance 400 (at 400 MHz), a Bruker Avance 500 (at 500 MHz), a Bruker Avance 600 (at 600 MHz) and a Bruker Avance 800 (at 800 MHz). Multiplicities were given as singlet (s), doublet (d), doublet of doublets (dd), triplet (t), triplet of doublets (dt), or multiplet (m). Carbon nuclear magnetic resonance (^{13}C NMR) spectra were recorded on a Bruker Avance 400 (at 100 MHz), a Bruker Avance 500 (at 125 MHz), a Bruker Avance 600 (at 150 MHz) and a Bruker Avance 800 (at 200 MHz). Spectra were assigned using COSY, HSQC, TOCSY, and HMBC experiments. The stereochemistry of glycosidic linkage was confirmed by coupling constant between the anomeric proton and C2-proton ($J_{H1\text{-}H2}$) or coupling constant between the anomeric carbon and proton (J_{C1-H1}). ESI-MS data were recorded on a Shimadzu LC-MS2020. High-resolution mass spectrometry (HRMS) was measured on an ESI apparatus using an Agilent 1290 G6460A Q-TOF. Thin-layer chromatography (TLC) was carried out on Merck silica gel 60 F_{254}-coated aluminum sheets. TLC plates were detected with UV absorption (254 nm) and sprayed with 10% sulfuric acid in ethanol (1:9, v/v), followed by heating for visualization. Flash column chromatography was performed on a normal-phase silica column or C18 reversed-phase silica column. Size-exclusion chromatography was performed on a Sephadex LH-20, HW40F, Bio-Gel P-2 or P-4 (45-90 μm) column. Molecular sieves were activated prior to use. Chemical reagents were purchased from *J&K* Scientific Ltd. and TCI Shanghai, China. Cytidine-5′-monophospho-*N*-acetylneuraminic acid (CMP-Neu5Ac) was purchased from BioChemSyn. Neu5Ac-α2,6GalNAc-α-PAA-biotin was purchased from Sigma-Aldrich. C18 ceramide (d18:1/18:0) was purchased from Avanti Polar Lipids, Inc. Biotinylated human Siglec-7 and Siglec-10 were purchased from ACROBiosystems. Calf intestine alkaline phosphatase (CIAP) was purchased from BioLabs® Inc.

2. Glycosyltransferase expression

2.1 Bacterial glycosyltransferase expression

Bacterial glycosyltransferases *Pasteurella multocida* α2,3-sialyltransferase 1 M144D mutant (PmST1 M144D),[1] *Campylobacter jejuni* α2,3-sialyltransferase I (Cst-I),[2] *Bifidobacterium*

infantis D-galactosyl-β1,3-*N*-acetyl-D-hexosamine phosphorylase (BiGalHexNAcP)[3] and *Escherichia coli* K-12 galactokinase (GalK)[4-5] were prepared as described below. PmST1 M144D, BiHexNAcP and GalK were cloned into the pET-28a vector with six histidines at N-terminal. Cst-I was cloned in a vector with MBP tag and six histidines tag. The confirmed constructs were subsequently transformed into *E.coli* BL21 (DE3) for protein expression. *E. coli* BL21 (DE3) cells harboring recombinant vector were cultured in two liters of LB medium containing kanamycin (50 μg/mL) in a rotary shaker (200 rpm) at 37°C. IPTG (0.2 mM) was added until OD was 0.8, and then protein expression was allowed to proceed at 16 °C for overnight. The cells were harvested by centrifugation at 7000 rpm for 10 min. The cell precipitation was re-suspended in lysis buffer (50 mM Tris-HCl buffer, 300 mM NaCl, 10 mM imidazole, pH 7.5). Cells were disrupted by a microfluidizer and the lysate was centrifuged at 12,000 g for 10 minutes to remove the cell debris. His-tagged proteins were purified by using Ni-NTA agarose column. Before purification, the column was equilibrated with the lysis buffer (50 mM Tris-HCl, 300 mM NaCl, 10 mM imidazole, pH 7.5). The column was washed with 2 column volumes of the lysis buffer and eluted with elution buffer (50 mM Tris-HCl, 300 mM NaCl, 300 mM imidazole, pH 7.5). The enzyme was desalted by filtration (Amicon Ultra-5, 10 kDa) for further use. Protein concentration was determined by Bicinchoninic acid (BCA) Protein Assay Kit. In addition, SDS protein gels of these four enzymes were shown in Figure S1.

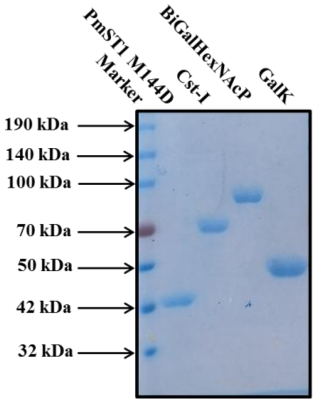

Figure S1. SDS protein gels of PmST1 M144D, Cst-I, BiHexNAcP, and GalK

2.2 Human glycosyltransferase expression

Human glycosyltransferases ST3Gal1 and ST6GalNAc5 were expressed as soluble, secreted

fusion proteins by transient transfection of HEK293 suspension cultures.[6-7] The coding regions were amplified from Mammalian Gene Collection clones, human tissue cDNAs, or generated by gene synthesis by a process that appended a tobacco etch virus (TEV) protease cleavage site to the NH_2-terminal end of the coding region and attL1 and attL2 Gateway adaptor sites were extended on the 5′ and 3′ terminal ends of the coding region during transfer to pDONR221 vector backbone. The pDONR221 clones were then recombined via LR clonase reaction into a custom Gateway adapted version of the pGEn2 mammalian expression vector to assemble a recombinant coding region comprised of a 25 amino acid NH_2-terminal signal sequence from the *T. cruzi* lysosomal *α*-mannosidase followed by an 8xHis tag, 17 amino acid AviTag, "superfolder" GFP, the nine amino acid sequence encoded by attB1 recombination site, followed by the TEV protease cleavage site and the respective glycosyltransferase catalytic domain coding region.

Suspension culture HEK293 cells (Freestyle 293-F cells, Life Technologies, Grand Island, NY) were transfected as previously described[7] and the culture supernatant was subjected to Ni^{2+}-NTA superflow chromatography (Qiagen, Valencia, CA). Enzyme preparations were eluted with 300 mM imidazole, concentrated by ultrafiltration, and subjected to gel filtration on a Superdex 75 column (GE Healthcare) preconditioned with a buffer containing 20 mM HEPES, pH 7.0, 100 mM NaCl, 10% glycerol, and 0.05% sodium azide. Peak fractions were pooled and concentrated to ~1 mg/mL using an ultrafiltration pressure cell membrane (Millipore, Billerica, MA) with a 10 kDa molecular weight cutoff. In addition, SDS protein gels of ST3Gal1 and ST6GalNAc5 were shown in Figure S2.

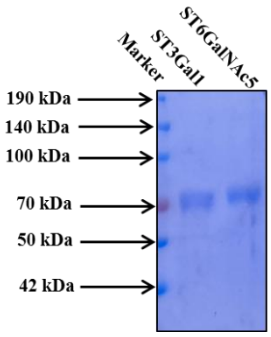

Figure S2. SDS protein gels of ST3Gal1 and ST6GalNAc5

3. The labels of monosaccharide residues for NMR nomenclature

The monosaccharide residues of DSGb5 glycolipid were labeled as shown in Figure S3. Starting from the reducing end, neutral sugar residues were labeled as Glc-I, Gal-II, Gal-III, GalNAc-IV, and Gal-V. The α2,3-linked sialic acid residue was labeled as Neu5Ac-VI and the α2,6-linked sialic acid residue was labeled as Neu5Ac-VII.

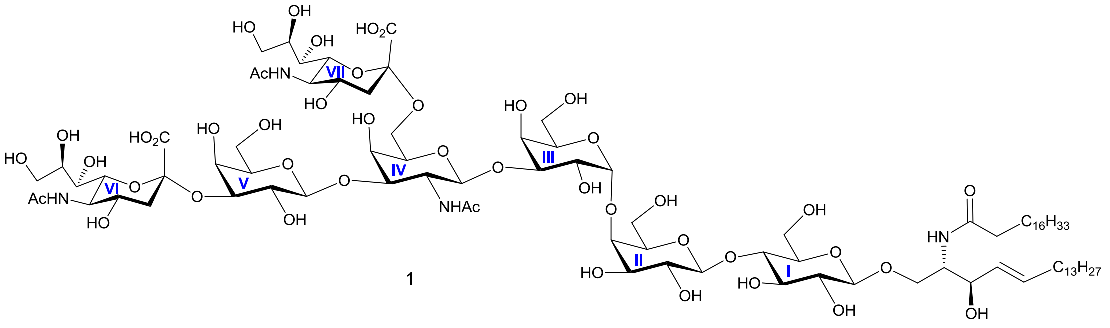

Figure S3. The labels of monosaccharide residues of DSGb5 glycolipid

4. Chemoenzymatic synthesis of disaccharide oxazoline donor 5

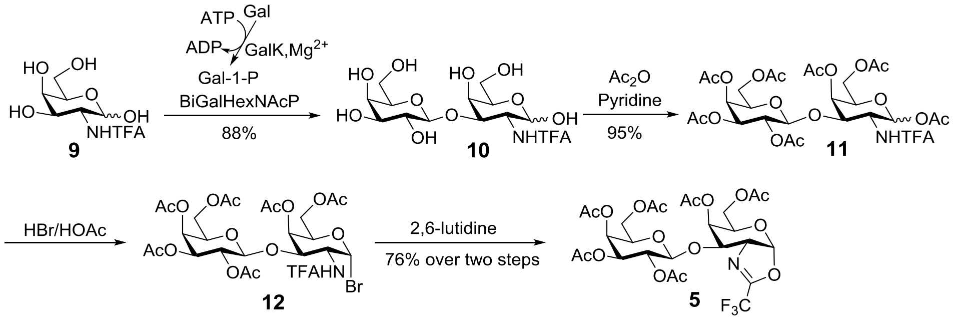

Scheme S1. Synthesis of oxazoline donor **5**

β-D-Galactopyranosyl-(1→3)-2-deoxy-2-trifluoroacetamido-D-galactopyranose **(10)**

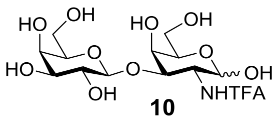

To a 10 mL solution of GalNHTFA (110.0 mg, 0.400 mmol), galactose (86.4 mg, 0.48 mmol,), ATP (330.7 mg, 0.58 mmol) and $MgCl_2$ (10 mM) in Tris-HCl buffer (100 mM, pH 6.5) was added GalK (4.0 mg) and BiGalHexNAcP (3.0 mg), and the reaction mixture was incubated for 24 h at 37 °C.[3] Subsequently, TLC analysis was performed (CH_3CN/H_2O 4:1) to monitor the reaction every 12 h and the additional enzymes GalK (2.0 mg) and BiGalHexNAcP (1.5 mg) were added until the starting material GalNHTFA disappeared. The reaction was quenched by adding the same volume of ice-cold EtOH (10 mL) and incubating

at 4 °C for 30 min. The mixture was then centrifuged to remove precipitates. The resulting supernatant was concentrated and purified by silica gel chromatography (EtOAc/MeOH/H_2O 4:1:0.1). Product containing fractions were combined and concentrated for further purification by a BioGel P-2 gel filtration column (eluent: H_2O) to afford **10** as a white amorphous solid (154 mg, 88%). ^{1}H NMR (600 MHz, D_2O): δ 5.28 (d, J = 3.7 Hz, 0.4 H, H1α-GalNHTFA), 4.78 (d, J = 8.6 Hz, 0.6 H, H1β-GalNHTFA'), 4.49 (d, J = 7.8 Hz, 0.4 H, H1-Gal), 4.42 (d, J = 7.8 Hz, 0.6 H, H1-Gal'), 4.38 (dd, J = 11.1, 3.7 Hz, 0.4 H, H2-GalNHTFA), 4.29 – 4.26 (m, 0.4 H), 4.22 (d, J = 3.2 Hz, 0.6 H, H4-GalNHTFA'), 4.19 – 4.14 (m, 1H), 4.07 (dd, J = 11.0, 8.4 Hz, 0.6 H, H2-GalNHTFA'), 3.95 (dd, J = 10.9, 3.2 Hz, 0.6 H, H3-GalNHTFA'), 3.90 – 3.88 (m, 1H), 3.80 – 3.70 (m, 4.4 H), 3.67 – 3.62 (m, 1H), 3.61 – 3.57 (m, 1H, H3-Gal, H3-Gal'), 3.52 – 3.47 (m, 1H, H2-Gal, H2-Gal'); ^{13}C NMR (150 MHz, D_2O): δ 159.5 – 158.4 (COCF$_3$, COCF$_3$'), 116.3 – 114.3 (COCF$_3$, COCF$_3$'), 104.4 (C1-Gal), 104.2 (C1-Gal'), 94.0 (C1-GalNHTFA), 90.2 (C1-GalNHTFA'), 79.1, 76.0, 74.5, 74.4, 72.1, 72.0, 70.1, 70.0, 69.7, 68.2, 68.1, 67.4, 60.6, 60.5, 60.4, 60.3, 52.6, 49.4; HRMS (ESI) calcd for $C_{14}H_{26}F_3N_2O_{11}$ $[M+NH_4]^+$ 455.1483, found 455.1482.

(2,3,4,6-Tri-O-acetyl-β-D-galactopyranosyl)-(1→3)-1,4,6-tri-O-acetyl-2-deoxy-2-trifluoro-acetamido-D-galactopyranoside **(11)**

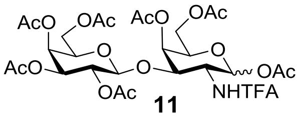

11

To a solution of compound **10** (109.5 mg, 0.25 mmol) in pyridine (2.0 mL) and Ac_2O (1.0 mL) was added DMAP (cat.). The mixture was stirred overnight at room temperature. TLC analysis showed complete conversion of the starting material to a major product **11** (petroleum ether/ethyl acetate 1:2, v/v, R_f = 0.57). The reaction was quenched by the addition of dry MeOH and stirred for 10 min. The reaction mixture was concentrated under reduced pressure to afford yellow oil, which was diluted with ethyl acetate and washed with 1 M hydrochloric acid, saturated aqueous solution of $NaHCO_3$, and water in sequence. The organic layer was dried over anhydrous Na_2SO_4 and filtered. The filtrate was concentrated under reduced pressure. The resulting residue was purified by silica gel column chromatography (petroleum ether/ethyl acetate 2:1) to afford compound **11** as colorless oil (173.8 mg, 95%). ^{1}H NMR (400 MHz, $CDCl_3$, **11β**): δ 6.63 (d, J = 7.5 Hz, 1H, NH), 6.34 (d, J = 3.5 Hz, 1H,

H1-GalNHTFA), 5.42 (d, J = 2.7 Hz, 1H, H4-GalNHTFA), 5.37 (d, J = 3.3 Hz, 1H, H4-Gal), 5.19 (dd, J = 10.3, 7.9 Hz, 1H, H2-Gal), 4.95 (dd, J = 10.3, 3.4 Hz, 1H, H3-Gal), 4.70 (d, J = 8.0 Hz, 1H, H1-Gal), 4.54 (ddd, J = 11.0, 7.5, 3.6 Hz, 1H, H2-GalNTFA), 4.22 (t, J = 6.6 Hz, 1H), 4.18 – 4.07 (m, 4H), 4.04 – 3.92 (m, 2H), 2.17 (s, 3H, CH_3CO), 2.16 (s, 3H, CH_3CO), 2.14 (s, 3H, CH_3CO), 2.06 (s, 6H, 2 × CH_3CO), 2.05 (s, 3H, CH_3CO), 1.97 (s, 3H, CH_3CO); ^{13}C NMR (100 MHz, $CDCl_3$): δ 170.7 (C=O), 170.6 (C=O), 170.2 (C=O), 170.1 (2 × C=O), 169.7 (C=O), 168.6 (C=O), 158.6 – 156.9 (COCF$_3$), 118.1 – 116.1 (COCF$_3$), 100.0 (C1-Gal), 90.6 (C1-GalNTFA), 71.4, 71.1, 70.9, 69.3, 68.1, 67.8, 66.7, 61.7, 60.9, 48.9, 20.9 (CH_3CO), 20.8 (3 × CH_3CO), 20.7 (2 × CH_3CO), 20.6 (CH_3CO); HRMS (ESI) calcd for $C_{28}H_{36}F_3NNaO_{18}$ $[M+Na]^+$ 754.1777, found 754.1762.

***(2,3,4,6-Tri-O-acetyl-β-D-galactopyranosyl)-(1→3)-2-trifluoromethyl-(4,6-di-O-acetyl-2-deoxy-α-D-galactopyrano)-oxazoline* (5)**

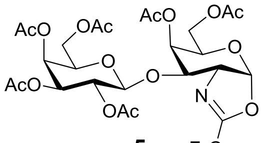

5

The compound **11** (117.0 mg, 0.16 mmol) was co-evaporated three times with toluene (3 × 3 mL) *in vacuo*. The resulting oil was dissolved in anhydrous CH_2Cl_2 (6 mL), followed by the addition of a solution of 33% hydrogen bromide in acetic acid (3 mL) under an atmosphere of argon. The reaction mixture was stirred at room temperature for 3 h. The mixture was concentrated under reduced pressure. The resulting oil was co-evaporated two times with toluene (2 × 3 mL) *in vacuo* to give yellow oil which was immediately used for next step.

The resulting bromide was dissolved in anhydrous CH_3CN (3 mL), followed by the addition of 2,6-lutidine (28 µL, 0.24 mmol) under an atmosphere of argon. The reaction mixture was stirred at room temperature for 1.5 h. The mixture was concentrated under reduced pressure. The resulting residue was purified by silica gel column chromatography (toluene/acetone 3:1) to afford oxazoline **5** as a white amorphous solid (81.5 mg, 76% over two steps). ^{1}H NMR (400 MHz, $CDCl_3$): δ 6.24 (d, J = 7.1 Hz, 1H), 5.43 (d, J = 2.2 Hz, 1H), 5.37 (d, J = 3.0 Hz, 1H, H4-Gal), 5.17 (dd, J = 10.5, 7.8 Hz, 1H, H2-Gal), 5.02 (dd, J = 10.5, 3.4 Hz, 1H, H3-Gal), 4.77 (d, J = 7.9 Hz, 1H, H1-Gal), 4.19 – 4.03 (m, 7H), 3.90 (t, J = 6.6 Hz, 1H), 3.78 (dd,

J = 7.4, 3.6 Hz, 1H), 2.14 (s, 3H, CH_3CO), 2.10 (s, 3H, CH_3CO), 2.07 (s, 3H, CH_3CO), 2.07 (s, 3H, CH_3CO), 2.03 (s, 3H, CH_3CO), 1.97 (s, 3H, CH_3CO); ^{13}C NMR (100 MHz, $CDCl_3$): δ 170.6 (*C*=O), 170.5 (*C*=O), 170.4 (*C*=O), 170.2 (*C*=O), 169.6 (*C*=O), 169.5 (*C*=O), 156.7 – 154.7 (*C*OCF_3), 117.9 – 113.9 (CO*C*F_3), 105.6, 100.3 (C1-Gal), 76.8, 71.1, 71.0, 70.6, 68.8, 67.0, 65.7, 64.6, 62.2, 61.3, 60.5, 20.9 (2 × CH_3CO), 20.8 (CH_3CO), 20.7 (2 × CH_3CO), 20.6 (CH_3CO); HRMS (ESI) calcd for $C_{26}H_{33}F_3NO_{16}$ $[M+H]^+$ 672.1746, found 672.1747.

5. Synthesis of trisaccharide acceptor 18

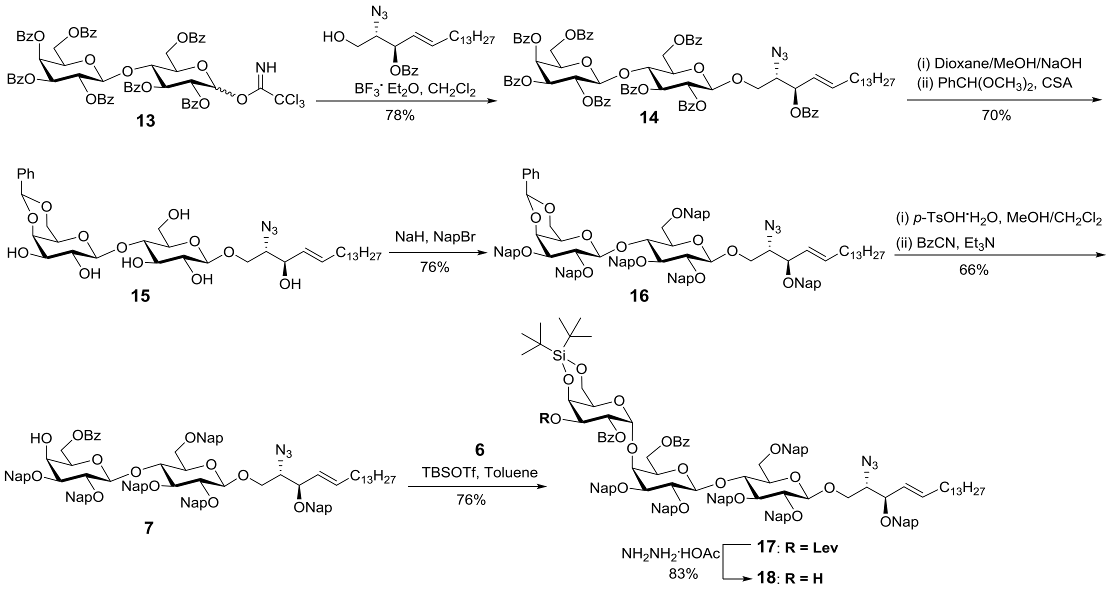

Scheme S2. Synthesis of trisaccharide acceptor **18**

***O*-(2,3,4,6-Tetra-*O*-benzoyl-*β*-D-galactopyranosyl)-(1→4)-(2,3,6-tri-*O*-benzoyl-*β*-D-gluco-pyranosyl)-(1→1)-(2S, 3R, 4E)-2-azido-3-*O*-benzoyloxy-octadec-4-ene** (**14**)

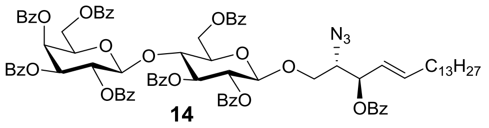

To a solution of perbenzoylated lactosyl trichloroacetimidate **13**[8] (270 mg, 0.22 mmol) and acceptor (2S,3R,4E)-2-azido-3-*O*-benzoyloxy-octadec-4-ene-1-ol[9] (90 mg, 0.21 mmol) in anhydrous CH_2Cl_2 (4 mL) was added freshly activated molecular sieves (4 Å, 250 mg). The mixture was stirred at room temperature for 30 min under an atmosphere of argon. The reaction mixture was cooled to -18 °C, followed by the addition of $BF_3·Et_2O$ (18.7 μL, 0.21 mmol). The reaction mixture was stirred at -18 °C for 30 min and the temperature was slowly warmed to 0 °C. The mixture continued to be stirred at 0 °C until TLC analysis (petroleum

ether/ethyl acetate 3:1) showed complete consumption of the acceptor. Subsequently, the reaction was quenched with Et_3N and the mixture was filtered through Celite. The filtrate was concentrated under reduced pressure to give a residue, which was purified by silica gel chromatography (petroleum ether/EtOAc 10:1→3:1) to afford compound **14** as colorless oil (291.8 mg, 78%). ^{1}H NMR (600 MHz, $CDCl_3$): δ 8.03 – 7.94 (m, 12H, H-Ar), 7.92 – 7.88 (m, 2H, H-Ar), 7.74 – 7.71 (m, 2H, H-Ar), 7.64 – 7.57 (m, 2H, H-Ar), 7.56 – 7.46 (m, 7H, H-Ar), 7.42 – 7.33 (m, 9H, H-Ar), 7.32 –7.28 (m, 2H, H-Ar), 7.23 – 7.19 (m, 2H, H-Ar), 7.18 – 7.13 (m, 2H, H-Ar), 5.81 (t, *J* = 9.3 Hz, 1H, H3-Gal-II), 5.75 – 5.65 (m, 3H, H2-Glc-I, H4-Glc-I, -CH=*CH*-), 5.54 – 5.47 (m, 2H), 5.42 (dd, *J* = 15.4, 7.5 Hz, 1H, -*CH*=CH-), 5.38 (dd, *J* = 10.3, 3.5 Hz, 1H, H3-Glc-I), 4.88 (d, *J* = 7.9 Hz, 1H, H1-Glc-I), 4.74 (d, *J* = 7.7 Hz, 1H, H1-Gal-II), 4.58 (dd, *J* = 12.1, 1.9 Hz, 1H, H6a-Gal-II), 4.47 (dd, *J* = 12.1, 4.4 Hz, 1H, H6b-Gal-II), 4.29 (t, *J* = 9.5 Hz, 1H), 3.92 – 3.88 (m, 2H, H5-Glc-I, *CH*H-octadec), 3.87 – 3.83 (m, 2H, H5-Gal-II, H6a-Glc-I), 3.73 (m, 2H, CH_2-octadec), 3.55 (dd, *J* = 10.2, 5.8 Hz, 1H, H6b-Glc-I), 1.92 – 1.85 (m, 2H, CH_2-octadec), 1.31 – 1.14 (m, 22H, CH_2-octadec), 0.88 (t, *J* = 7.0 Hz, 3H, CH_3-octadec); ^{13}C NMR (150 MHz, $CDCl_3$): δ 165.81(*C*=O), 165.57 (*C*=O), 165.42 (*C*=O), 165.42 (*C*=O), 165.24 (*C*=O), 165.03 (*C*=O), 164.94 (*C*=O), 164.83 (*C*=O), 138.96 (-CH=*CH*-), 133.55-128.26 (*C*-Ar), 122.42 (-*CH*=CH-), 101.03 (C1-Glc-I), 100.83 (C1-Gal-II), 75.87, 74.78 (*C*H-octadec), 73.13, 72.87, 71.78, 71.65, 71.43, 69.89, 68.28 (C6-Glc-I), 67.54, 63.42 (C5-Glc-I), 62.27 (C6-Gal-II), 61.07 (CH_2-octadec), 32.27 (CH_2-octadec), 31.94 (CH_2-octadec), 29.71-22.71 (CH_2-octadec), 14.21 (CH_2-octadec), 14.15 (CH_3-octadec); HRMS (ESI) calcd for $C_{86}H_{87}N_3NaO_{20}$ $[M+Na]^+$ 1504.5775, found 1504.5761.

***O*-(4,6-Benzylidene-β-D-galactopyranosyl)-(1→4)-(β-D-glucopyranosyl)-(1→1)-(2S,3R,4E)-2-azido-3-*O*-benzoyloxy-octadec-4-ene** (**15**)

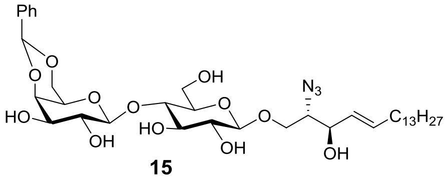

15

A solution of compound **14** (295 mg, 0.2 mmol) in dioxane (6 mL), MeOH (2 mL) and NaOH (1 M, 2 mL) was stirred at room temperature for 8 h. TLC analysis showed complete conversion of the starting material to a major product (EA/MeOH/H_2O/AcOH 5:1:1:0.5, R_f =

0.51). Subsequently, the pH of the mixture was neutralized to ~7 by Amberlite IR120 hydrogen form resin. The reaction mixture was filtered, and the filtrate was concentrated *in vacuo* to afford a residue for next step. The resulting residue was dissolved in anhydrous DMF (2 mL) under an atmosphere of argon, followed by the addition of benzaldehyde dimethylacetal (35 μL, 0.24 mmol) and camphorsulfonic acid (CSA, 13.6 mg, 0.06 mmol). The reaction mixture was stirred overnight at room temperature. TLC analysis showed complete conversion of starting material to a major product (EA/MeOH/H_2O/AcOH 10:1:1:0.5, R_f = 0.5). The reaction was quenched by the addition of triethylamine and the mixture was concentrated under reduced pressure to give a residue. The resulting residue was purified by silica gel column chromatography (MeOH in dichloromethane, 10%) to afford compound **15** as a white amorphous solid (103 mg, 70%). ^{1}H NMR (500 MHz, $CDCl_3$: CD_3OD 1:1): δ 7.52 – 7.48 (m, 2H, H-Ar), 7.35 – 7.31 (m, 3H, H-Ar), 5.75 (dt, J = 15.4, 6.7 Hz, 1H, -CH=*CH*-), 5.58 (s, 1H, *CH*-benzylidene), 5.49 (dd, J = 15.4, 7.5 Hz, 1H, -*CH*=CH-), 4.45 (d, J = 7.7 Hz, 1H, H1-Glc-I), 4.30 (d, J = 7.8 Hz, 1H, H1-Gal-II), 4.24 – 4.09 (m, 4H), 3.94 – 3.85 (m, 3H, including H3-Glc-I, CH_2-octadec), 3.73 – 3.68 (m, 2H), 3.64 – 3.56 (m, 5H), 3.39 (d, J = 9.4 Hz, 1H), 3.31 (dd, J = 3.2, 1.6 Hz, 1H), 2.08-2.02 (m, 2H, CH_2-octadec), 1.41-1.35 (m, 2H, CH_2-octadec), 1.28-1.23 (m, 20H, CH_2-octadec), 0.86 (t, J = 6.9 Hz, 3H, CH_3-octadec); ^{13}C NMR (125 MHz, $CDCl_3$: CD_3OD 1:1): δ 134.33 (-CH=*CH*-), 128.21 (*C*-Ar), 127.46 (-*CH*=CH-), 127.28 (*C*-Ar), 125.57 (*C*-Ar), 102.87 (C1-Glc-I), 102.33 (C1-Gal-II), 100.49 (*CH*-benzylidene), 78.04, 75.23, 74.50, 74.23, 72.67, 71.55, 71.49, 69.66, 68.34, 68.26, 66.33, 65.13, 59.94, 31.60 (CH_2-octadec), 31.16 (CH_2-octadec), 28.89-28.30 (CH_2-octadec), 21.87 (CH_2-octadec), 12.92 (CH_3-octadec); HRMS (ESI) calcd for $C_{37}H_{63}N_4O_{12}$ $[M+NH_4]^+$ 755.4437, found 755.4434.

O-(2,3-Di-O-2-naphthyl)methyl-4,6-benzylidene-β-D-galactopyranosyl)-(1→4)-(2,3,6-tri-O-2-naphthyl)methyl-β-D-glucopyranosyl)-(1→1)-(2S,3R,4E)-2-azido-3-O-2-naphthyl)methyl -octadec-4-ene **(16)**

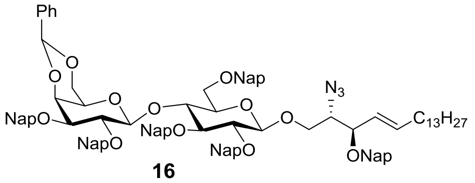

16

To a solution of compound **15** (230 mg, 0.26 mmol) and 2-(bromomethyl)naphthalene (476 mg, 2.2 mmol) in anhydrous DMF (5 mL) was added NaH (60% dispersion in oil, 52.8 mg, 2.2 mmol) at 0 °C, and then the mixture was warmed to room temperature in 30 min and stirred overnight. TLC analysis showed complete conversion of starting material to a major product (petroleum ether/EtOAc, 3:1, R_f = 0.52). The reaction was quenched with MeOH (0.5 mL) and the mixture was diluted with EtOAc (15 mL), washed with saturated aqueous NH_4Cl (5 mL), and brine (5 mL) in sequence. The organic layer was dried over anhydrous Na_2SO_4 and filtered. The filtrate was concentrated under reduced pressure to give a residue. The resulting residue was purified by silica gel column chromatography (petroleum ether/EtOAc, 6:1) to afford compound **16** (310 mg, 76%) as colorless oil. ^{1}H NMR (500 MHz, $CDCl_3$): δ 7.84 – 7.65 (m, 20H, H-Ar), 7.63 – 7.53 (m, 7H, H-Ar), 7.49 – 7.37 (m, 16H, H-Ar), 7.32 – 7.27 (m, 4H, H-Ar), 5.67 (dt, *J* = 15.4, 6.7 Hz, 1H, -CH=*CH*-), 5.43 (s, 1H, *CH*-benzylidene), 5.46-5.37 (m, 2H, -*CH*=CH-, *CH*H-Nap), 5.02-4.95 (m, 2H), 4.95 – 4.86 (m, 3H), 4.83 – 4.73 (m, 2H), 4.71-4.65 (m, 2H), 4.50 – 4.39 (m, 4H, H1-Gal-II, 2 x *CH*H-Nap, H1-Glc-I), 4.24 – 4.18 (m, 1H), 4.11 – 3.95 (m, 5H), 3.86 (dd, *J* = 9.6, 7.8 Hz, 1H, H2-Gal-II), 3.81 – 3.69 (m, 4H), 3.65 (dd, *J* = 10.3, 5.3 Hz, 1H), 3.53 (dd, *J* = 9.2, 7.8 Hz, 1H, H2-Glc-I), 3.41 – 3.36 (m, 1H, H5-Glc-I), 3.29 (dd, *J* = 9.7, 3.6 Hz, 1H, H3-Gal-II), 2.88-2.84 (m, 1H), 2.06-2.00 (m, 2H, CH_2-octadec), 1.28-1.22 (m, 22H, CH_2-octadec), 0.88 (t, *J* = 6.9 Hz, 3H, CH_3-octadec); ^{13}C NMR (125 MHz, $CDCl_3$): δ 138.46 (-CH=*CH*-), 138.12-125.82 (*C*-Ar), 125.76 (-*CH*=CH-), 125.72-125.34 (*C*-Ar), 103.61 (C1-Glc-I), 102.83 (C1-Gal-II), 101.39 (*CH*-benzylidene), 82.96 (C3-Glc-I), 81.73 (C2-Glc-I), 79.80 (C3-Gal-II), 79.65, 79.02 (C2-Gal-II), 77.61 (C5-Glc-I), 75.76 (C4-Glc-I), 75.44, 75.26, 74.97, 73.54 (C4-Gal-II), 73.16, 71.58, 70.01, 68.97, 68.55, 68.15, 66.33 (C5-Gal-II), 64.49, 32.43 (CH_2-octadec), 31.94 (CH_2-octadec), 29.72-29.05 (CH_2-octadec), 22.71 (CH_2-octadec), 14.14 (CH_3-octadec); HRMS (ESI) calcd for $C_{103}H_{107}N_3NaO_{12}$ $[M+Na]^+$ 1600.7747, found 1600.7755.

***O-(2,3-Di-O-2-naphthyl)methyl-6-benzoyl-β-D-galactopyranosyl)-(1→4)-(2,3,6-tri-O-2-naphthyl)methyl-β-D-glucopyranosyl)-(1→1)-(2S, 3R, 4E)-2-azido-3-O-2-naphthyl)methyl-octadec-4-ene* (7)**

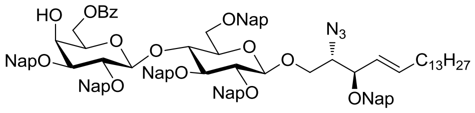

7

To a solution of compound **16** (158 mg, 0.10 mmol) was dissolved in MeOH (4 mL) and DCM (1 mL) was cooled to 0 °C, followed by the addition of TsOH·H_2O (9.5 mg, 0.05 mmol). The reaction was stirred until TLC showed conversion of starting material to a major product (toluene/acetone 4:1, R_f = 0.6). The reaction was quenched by saturated aqueous solution of $NaHCO_3$. The reaction mixture was concentrated under reduced pressure to a residue, which was re-dissolved in EtOAc (15 mL) and washed with water (7 mL). The organic layer was dried over anhydrous Na_2SO_4 and filtered. The filtration was concentrated under reduced pressure to give a residue. The resulting residue was co-evaporated with toluene twice (2 × 3 mL) *in vacuo*, which was used for next step without any further purification.

To a solution of the resulting residue in anhydrous DMF (3 mL) was added triethylamine (16.6 µL, 0.12 mmol). After cooling this mixture to -20 °C, benzoyl cyanide (11.8 µL, 0.1 mmol) was added. The reaction temperature was slowly warmed to 0 °C in 15 min and stirred for another 30 min at 0 °C. TLC analysis showed complete conversion of the starting material to a major product (toluene/acetone 8:1, R_f = 0.7). The reaction mixture was diluted with ethyl acetate (12 mL) and washed with water (6 mL). The organic layer was dried over anhydrous Na_2SO_4 and filtered. The filtrate was concentrated under reduced pressure to give a residue. The resulting residue was purified by silica gel column chromatography (toluene/acetone 16:1) to afford compound **7** as colorless oil (105 mg, 66% over two steps). ^{1}H NMR (600 MHz, $CDCl_3$): δ 8.01-7.95 (m, 2H, H-Ar), 7.86 – 7.61 (m, 24H, H-Ar), 7.58 – 7.30 (m, 21H, H-Ar), 5.69 (dt, J = 15.4, 6.7 Hz, 1H, -CH=*CH*-), 5.45 (dd, J = 15.6, 7.5 Hz, 1H, -*CH*=CH-), 5.18 (d, J = 11.1 Hz, 1H, *CH*H-Nap), 4.96-4.85 (m, 4H), 4.83 (d, J = 11.5 Hz, 1H, *CH*H-Nap), 4.77 – 4.67 (m, 3H, 3 x *CH*H-Nap), 4.64 (d, J = 11.9 Hz, 1H, *CH*H-Nap), 4.56 – 4.50 (m, 1H), 4.49-4.43 (m, 2H), 4.41-4.36 (m, 2H, H1-Glc-I, H1-Gal-II), 4.32 (dd, J = 11.2, 6.6 Hz, 1H, H6-Gal-II), 4.11 (t, J = 9.4 Hz, 1H, H4-Glc-I), 4.04 (dd, J = 10.1, 6.6 Hz, 1H, *CH*H-octadec), 4.01-3.93 (m, 2H), 3.80-3.77 (m, 2H, H4-Gal-II, *CH*H-octadec), 3.74 (d, J = 10.8 Hz, 1H), 3.69-3.61 (m, 3H, H3-Glc-I, *CH*H-octadec, H2-Gal-II), 3.49 (t, J = 8.6 Hz, 1H, H2-Glc-I),

3.41-3.34 (m, 2H, H5-Glc-I, H5-Gal-II), 3.08 – 3.04 (m, 1H), 2.09-2.02 (m, 2H, CH_2-octadec), 1.33-1.23 (m, 22H, CH_2-octadec), 0.88 (t, J = 7.1 Hz, 3H, CH_3-octadec); ^{13}C NMR (150 MHz, $CDCl_3$): δ 166.27 (*C*=O), 138.47 (-CH=*CH*-), 136.54-125.48 (*C*-Ar, -*CH*=CH-), 103.64 (C1-Gal-II), 102.33 (C1-Glc-I), 82.53, 81.57 (C2-Glc-I), 80.90 (C3-Gal-II), 79.63, 79.34, 76.43 (C4-Glc-I), 75.28, 75.21, 75.16, 74.91, 73.38, 72.37, 71.84, 70.04, 68.59, 67.87, 66.23, 64.48, 62.87, 32.45 (CH_2-octadec), 31.95 (CH_2-octadec), 29.74-29.07 (CH_2-octadec), 22.72 (CH_2-octadec), 14.15 (CH_3-octadec); HRMS (ESI) calcd for $C_{103}H_{107}N_3NaO_{13}$ $[M+Na]^+$ 1616.7696, found 1616.7695.

***2-O-benzoyl-3-O-(2-naphthyl)methyl-4,6-O-di-tert-butylsilylene-β-D-galactopyranosyl (1→4)-2,3-di-O-(2-naphthyl)methyl-6-O-benzoyl-β-D-galactopyranosyl-(1→4)-2,3,6-tri-O-(2-naphthyl)methyl-β-D-glucopyranoside-(1→1)-(2S,3R,4E)-2-azido-3-O-(2-naphthyl)methyl-octadec-4-ene* (17)**

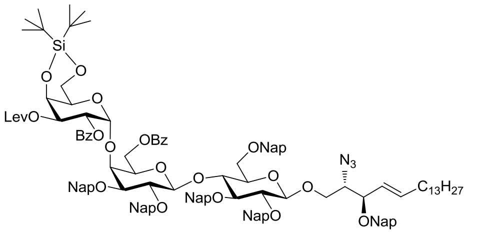

17

To a solution of *p*-tolyl 2-*O*-benzoyl-4,6-di-*O*-di-*tert*-butylsilylidene-3-*O*-levulinoyl-1-thio-β-D-galactopyranoside[10] (252 mg, 0.4 mmol) in acetone (4.5 mL) and PBS buffer (100 mM, pH 7.4, 0.5 mL) was added *N*-bromosuccinimide (214 mg, 1.2 mmol) and stirred at room temperature in the dark. After TLC analysis showed the disappearance of the starting material, the reaction mixture was diluted with EtOAc (20 mL) and washed with a saturated aqueous solution of $Na_2S_2O_4$ (8 mL). The aqueous layer was further extracted with EtOAc (2 × 15 mL). The combined organic phases were dried over anhydrous Na_2SO_4 and filtered. The filtration was concentrated under reduced pressure to give a residue, which was purified by silica gel column chromatography (petroleum ether/EtOAc, 3:1) to afford an intermediate. The resulting intermediate was dissolved in anhydrous DCM (10 mL), followed by the addition of 2,2,2-trifluoro-*N*-phenylacetimidoyl chloride (130 μL, 0.8 mmol) and Cs_2CO_3 (260 mg, 0.8 mmol) at room temperature. After the reaction mixture was stirred at room temperature overnight, the mixture was filtered and the filtrate was concentrated *in vacuo* to

give a residue. The resulting residue was purified by silica gel column chromatography (Petroleum ether/EtOAc, 6:1→2:1) to afford glycosyl donor **6** as a white amorphous solid (202 mg, 73%), which was immediately used for next coupling reaction.

To a solution of the resulting glycosyl donor **6** (55 mg, 0.08 mmol) and acceptor **7** (63 mg, 0.04 mmol) in anhydrous toluene (2 mL) was added freshly activated molecular sieves (4 Å, 120 mg). The mixture was stirred at room temperature for 30 min under an atmosphere of argon. After the reaction mixture was cooled to -20 °C, a catalytic amount of TBSOTf (1 μL, 0.004 mmol) was added and stirred at -20 °C for 30 min. Subsequently, the reaction temperature was slowly warmed to 0 °C. After TLC analysis showed complete consumption of the acceptor to a major product (petroleum ether/EtOAc 4:1, R_f = 0.5), the reaction was quenched with Et_3N and filtered through Celite. The filtrate was concentrated *in vacuum* to give a residue, which was purified by silica gel column chromatography (toluene/acetone 16:1) to afford compound **17** as colorless oil (64 mg, 76%). ^{1}H NMR (500 MHz, $CDCl_3$): δ 7.91 – 7.88 (m, 2H, H-Ar), 7.84 – 7.72 (m, 15H, H-Ar), 7.72 – 7.65 (m, 6H, H-Ar), 7.60 – 7.55 (m, 2H, H-Ar), 7.52 – 7.33 (m, 20H, H-Ar), 7.30 – 7.21 (m, 5H, H-Ar), 7.02-6.99 (m, 2H, H-Ar), 5.68 – 5.60 (m, 2H, -CH=*CH*-, H2-Gal-III), 5.46 – 5.37 (m, 3H, H1-Gal-III, -*CH*=CH-, H3-Gal-III), 5.06 (d, *J* = 12.3 Hz, 1H, *CH*H-Nap), 4.96 – 4.87 (m, 4H), 4.80-4.72 (m, 2H, 2 x *CH*H-Nap), 4.70-4.66 (m, 2H), 4.54 (d, *J* = 12.4 Hz, 1H, *CH*H-Nap), 4.49-4.45 (m, 3H), 4.44-4.41 (m, 2H, *CH*H-Nap, H1-Gal-II), 4.35 (d, *J* = 7.7 Hz, 1H, H1-Glc-I), 4.12 – 4.05 (m, 2H), 4.05 – 3.98 (m, 3H), 3.95 (dd, *J* = 8.6, 5.5 Hz, 1H, *CH*H-octadec), 3.84 (d, *J* = 3.0 Hz, 1H, H4-Gal-II), 3.78-3.72 (m, 3H), 3.71 – 3.65 (m, 2H, H3-Glc-I, H2-Gal-II), 3.65 – 3.59 (m, 1H), 3.50 – 3.45 (m, 2H), 3.37-3.33 (m, 1H, H5-Glc-I), 3.29 (m, 1H, H5-Gal-II), 2.94 (dd, *J* = 9.9, 3.0 Hz, 1H), 2.51 – 2.36 (m, 4H, CH_2-Lev), 2.05-1.98 (m, 2H, CH_2-octadec), 1.92 (s, 3H, CH_3-Lev), 1.29-1.23 (m, 22H, CH_2-octadec), 1.09 (s, 9H, 3 x CH_3-*t*-Bu), 0.92 (s, 9H, 3 x CH_3-*t*-Bu), 0.90 (t, *J* = 6.9 Hz, 3H, CH_3-octadec); ^{13}C NMR (125 MHz, $CDCl_3$): δ 205.81 (*C*=O-Lev), 172.00 (*C*=O), 166.32 (*C*=O), 165.47 (*C*=O), 138.46 (-CH=*CH*-), 136.69-125.47 (*C*-Ar, -*CH*=CH-), 103.53 (C1-Glc-I), 102.88 (C1-Gal-II), 98.58 (C1-Gal-III), 81.67 (C2-Glc-I), 81.41 (C2-Gal-II), 80.47 (C3-Gal-II), 79.61 (CH_2-octadec), 78.83 (C3-Glc-I), 77.31, 75.08, 74.74, 74.55, 73.36, 73.04, 71.49, 71.18, 70.47, 70.00, 69.04, 68.50 (C6-Glc-I), 67.95, 67.46, 66.46 (C6-Gal-III), 64.47 (CH_2-octadec), 61.22 (C6-Gal-II), 37.78 (CH_2-Lev), 32.42 (CH_2-

octadec), 31.95-29.52 (*CH_2*-octadec, *CH_3*-Lev), 28.14 (*CH_2*-Lev), 27.53-20.76 (*CH_2*-octadec, *$(CH_3)_3C$*-*t*-Bu), 14.16 (*CH_3*-octadec); HRMS (ESI) calcd for $C_{129}H_{143}N_3NaO_{21}Si$ $[M+Na]^+$ 2120.9876, found 2120.9875.

Table S1. Glycosylation of donor **6** with disaccharide acceptor **7a** or **7** to afford the protected Gb3 derivatives **17a** or **17**.

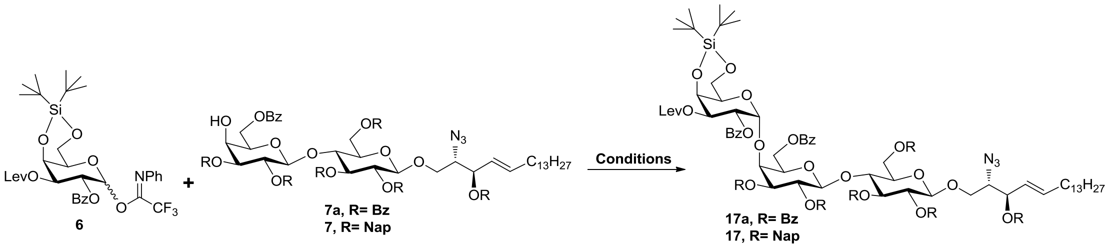

Entry	Acceptor	Product	Conditions	Yield(%)
1	**7a**	**17a**	TfOH, DCM, -20°C	30%
2	**7a**	**17a**	TBSOTf, DCM, -20°C	< 10%
3	**7a**	**17a**	TBSOTf, Toluene, -20°C	< 10%
4	**7**	**17**	TfOH, DCM, -20°C	< 10%
5	**7**	**17**	TBSOTf, Toluene, -20°C	76%

Glycosylation of donor **6** with disarmed acceptor (**7a**, R=Bz) did not give satisfactory results. Gratifyingly, the replacement of Bz with Nap (**7**, R=Nap) improved the activity of acceptor, TBSOTf-promoted glycosylation of donor **6** with armed acceptor **7** in toluene proceeded smoothly to afford the desired trisaccharide **17** in 76% yield. Additionally, 2-naphthylmethyl (Nap) ethers as permanent protecting groups could be selectively cleaved by 2,3-dichloro-5,6-dicyano-1,4-benzoquinone (DDQ)[11] to release hydroxyl groups without affecting the double bond of lipid moiety at the later stage of the synthesis.

***2-O-Benzoyl-4,6-O-di-tert-butylsilylene-β-D-galactopyranosyl-(1→4)-2,3-di-O-(2-naphthyl)methyl-6-O-benzoyl-β-D-galactopyranosyl-(1→4)-2,3,6-tri-O-(2-naphthyl)methyl-β-D-glucopyranoside-(1→1)-(2S,3R,4E)-2-azido-3-O-(2-naphthyl)methyl-octadec-4-ene* (18)**

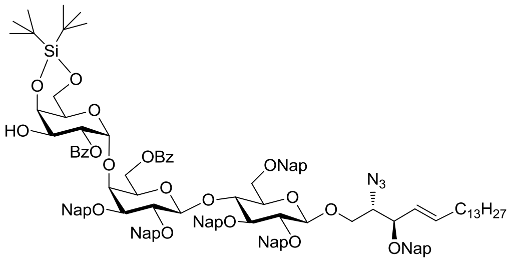

18

To a solution of **17** (63 mg, 0.03 mmol) in CH_2Cl_2 (0.6 mL) and CH_3OH (0.2 mL) was added $H_2NNH_2{\cdot}AcOH$ (11.9 mg, 0.15 mmol). The reaction mixture was stirred for 20 min at room temperature. TLC analysis showed complete conversion of starting material to a major product (petroleum ether/EtOAc 6:1, R_f = 0.5). The reaction mixture was diluted with DCM (5 mL) and washed with saturated brine (2 mL). The organic layer was dried over anhydrous Na_2SO_4 and filtered. The filtrate was concentrated *in vacuo* to give a residue. The resulting residue was purified by silica gel column chromatography (petroleum ether/EtOAc 8:1) to afford compound **18** as colorless oil (50 mg, 83%). ^1H NMR (400 MHz, $CDCl_3$): δ 7.90-7.83 (m, 4H, H-Ar), 7.82-7.73 (m, 8H, H-Ar), 7.73 – 7.57 (m, 16H, H-Ar), 7.57 – 7.51 (m, 2H, H-Ar), 7.49 – 7.32 (m, 17H, H-Ar), 7.32 – 7.27 (m, 1H, H-Ar), 7.25 – 7.16 (m, 2H, H-Ar), 7.03-6.92 (m, 2H, H-Ar), 5.68 (dt, J = 15.5, 6.7 Hz, 1H, -CH=*CH*-), 5.48 – 5.39 (m, 2H, -*CH*=CH-, H2-Gal-III), 5.29 (d, J = 3.5 Hz, 1H, H1-Gal-III), 5.17 (d, J = 12.1 Hz, 1H, *CH*H-Nap), 5.00-4.91 (m, 2H), 4.89 – 4.81 (m, 3H), 4.77 – 4.66 (m, 3H), 4.51 (d, J = 11.9 Hz, 1H, *CH*H-Nap), 4.49 – 4.43 (m, 4H, H1-Gal-II, 2 x *CH*H-Nap, H6a-Gal-II), 4.41 (d, J = 7.7 Hz, 1H, H1-Glc-I), 4.21-4.10 (m, 4H, H6b-Gal-II, H4-Gal-III, H4-Glc-I, H3-Gal-III), 4.06 (dd, J = 10.2, 6.4 Hz, 1H, *CH*H-octadec), 4.02-3.93 (m, 3H), 3.81 – 3.51 (m, 9H), 3.44 – 3.36 (m, 1H, H5-Glc-I), 3.28 (m, 1H, H5-Gal-II), 2.92 (dd, J = 9.9, 2.9 Hz, 1H, H3-Gal-II), 2.07-2.01 (m, 2H, CH_2-octadec), 1.31-1.23 (m, 22H, CH_2-octadec), 1.09 (s, 9H, 3 x CH_3-*t*-Bu), 0.95 (s, 9H, 3 x CH_3-*t*-Bu), 0.89 (t, J = 6.9 Hz, 3H, CH_3-octadec); ^{13}C NMR (100 MHz, $CDCl_3$): δ 166.93 (*C*=O), 165.45 (*C*=O), 138.48 (-CH=*CH*-), 136.55-125.44 (C-Ar, -*CH*=CH-), 103.57 (C1-Glc-I), 102.47 (C1-Gal-II), 98.95 (C1-Gal-III), 82.00, 81.52, 80.55 (C3-Gal-II), 79.51, 78.52, 76.49, 75.07, 74.85, 74.82, 74.55, 73.76, 73.37, 72.81, 72.22, 71.55, 69.95, 68.50, 68.12, 67.91, 67.64, 66.38, 64.40, 61.29 (C6-Gal-II), 32.40 (CH_2-octadec), 31.90-20.75 (CH_2-octadec, $(CH_3)_3C$-*t*-Bu), 14.12 (CH_3-octadec); HRMS (ESI) calcd for $C_{124}H_{137}N_3NaO_{19}Si$ $[M+Na]^+$ 2022.9508, found 2022.9510.

6. Synthesis of Gb5-sphingosine 2

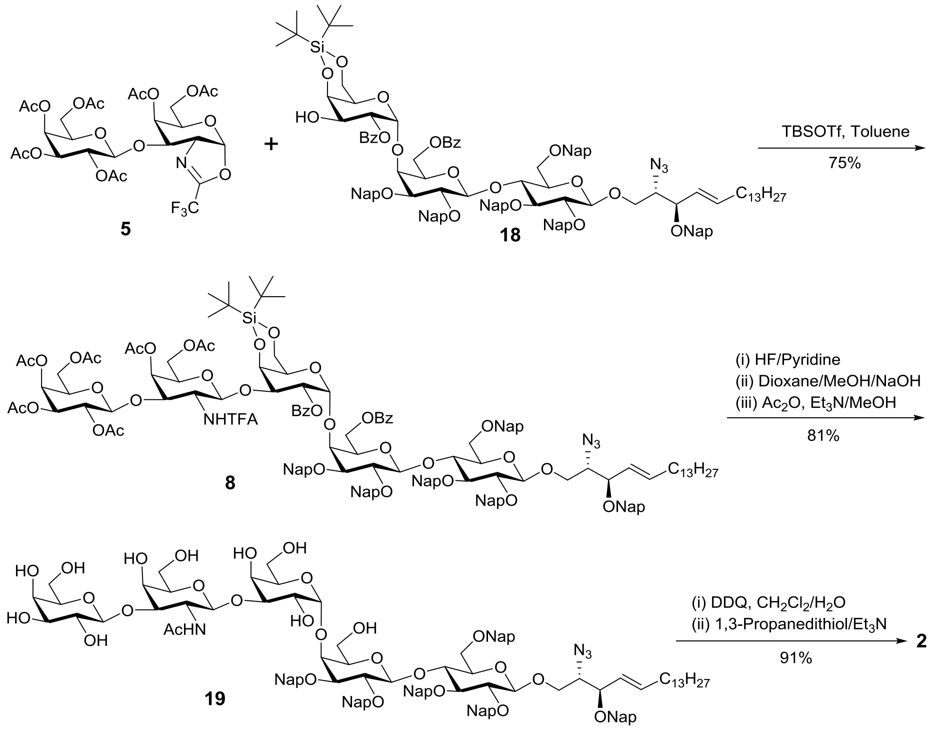

Scheme S3. Synthesis of Gb5-sphingosine **2**

(2,3,4,6-Tri-O-acetyl-β-D-galactopyranosyl)-(1→3)-(4,6-di-O-acetyl-2-deoxy-2-trifluoro-acetamido-α-D-galactopyranosyl)-(1→3)-(2-O-benzoyl-4,6-O-di-tert-butylsilylidene-α-D-galactopyranosyl)-(1→4)-(2,3-di-O-(2-naphthyl)methyl-6-benzoyl-β-D-galactopyranosyl)-(1→4)-(2,3,6-tri-O-(2-naphthyl)methyl-β-D-glucopyranosyl)-(1→1)-(2S, 3R, 4E)-2-azido-3-O-(2-naphthyl)methyl-octadec-4-ene **(8)**

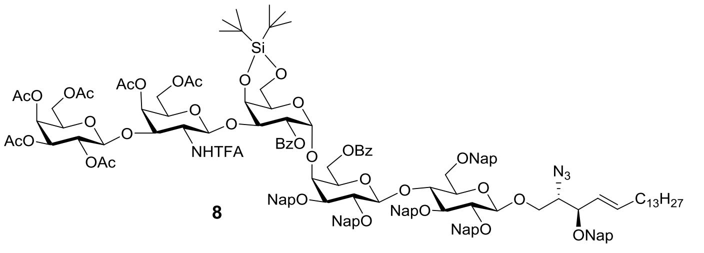

To a solution of oxazoline donor **5** (54 mg, 0.08 mmol) and acceptor **18** (80 mg, 0.04 mmol) in anhydrous toluene (2 mL) was added freshly activated molecular sieves (4 Å, 130 mg). The mixture was stirred at room temperature for 30 min under an atmosphere of argon. After the reaction mixture was cooled to -20 °C, a catalytic amount of TBSOTf (0.5 μL, 0.002 mmol) was added and stirred at -20 °C for 30 min. Subsequently, the reaction temperature was slowly warmed to 0 °C. After TLC analysis showed complete consumption of the acceptor to a major product (petroleum ether/EtOAc 2:1, $R_f = 0.3$). The reaction mixture was quenched

with Et_3N and filtered through Celite. The filtrate was concentrated *in vacuum* to give a residue, which was purified by silica gel column chromatography (petroleum ether/EtOAc 4:1→2:1) to afford compound **8** as colorless oil (80 mg, 0.03 mmol, 75%). ^{1}H NMR (600 MHz, $CDCl_3$): δ 8.02 – 7.97 (m, 2H, H-Ar), 7.86 – 7.52 (m, 30H, H-Ar), 7.50 – 7.30 (m, 18H, H-Ar), 7.25-7.19 (m, 2H, H-Ar), 6.04 (d, *J* = 7.5 Hz, 1H, *NH*), 5.72 – 5.59 (m, 2H, -CH=*CH*-, H2-Gal-III), 5.42 (dd, *J* = 15.5, 7.5 Hz, 1H, -*CH*=CH-), 5.33 – 5.30 (m, 2H, H1-Gal-III, H4-Gal-V), 5.14 (d, *J* = 11.4 Hz, 1H, *CH*H-Nap), 5.02 – 4.93 (m, 2H), 4.90-4.84 (m, 3H), 4.79 – 4.67 (m, 6H), 4.59-4.51 (m, 3H, including H1-GalNAc-IV and H6a-Gal-II), 4.49 – 4.34 (m, 5H, 3 x C*H*H-Nap , H1-Gal-II , H1-Glc-I), 4.30 – 4.18 (m, 2H, H1-Gal-V, H6b-Gal-II), 4.11-4.10 (m, 1H), 4.09 – 3.99 (m, 5H), 3.98 – 3.86 (m, 3H), 3.83 – 3.55 (m, 11H), 3.49-3.39 (m, 2H, H2-Glc-I, H2-GalNAc-IV), 3.33 – 3.23 (m, 2H, H5-Glc-I, H5-Gal-II), 2.88 (dd, *J* = 9.9, 2.9 Hz, 1H, H3-Gal-II), 2.77 (t, *J* = 6.6 Hz, 1H), 2.11 (s, 3H, CH_3-Ac), 2.06-2.04 (m, 5H, CH_3-Ac, CH_2-octadec), 2.03 (s, 3H, CH_3-Ac), 1.94 (s, 6H, 2 x CH_3-Ac), 1.91 (s, 3H, CH_3-Ac), 1.28 – 1.21 (m, 22H, CH_2-octadec), 1.10 (s, 9H, 3 x CH_3-*t*-Bu), 0.93 (s, 9H, 3 x CH_3-*t*-Bu), 0.87 (t, *J* = 6.9 Hz, 3H, CH_3-octadec); ^{13}C NMR (150 MHz, $CDCl_3$): δ 170.32 (CH_3*C*=O), 170.23 (CH_3*C*=O), 170.06 (CH_3*C*=O), 169.99 (CH_3*C*=O), 169.37 (CH_3*C*=O), 169.35 (CH_3*C*=O), 166.10 (Bz-*C*=O), 165.45 (Bz-*C*=O), 156.76 (CF_3*C*=O), 156.52 (CF_3*C*=O), 138.47 (-CH=*C*H-), 136.88-125.66 (*C*-Ar), 125.63 (-*C*H=CH-), 115.94 (*C*F_3C=O), 114.03 (*C*F_3C=O), 103.54 (C1-Glc-I), 102.20 (C1-Gal-II), 100.45 (C1-Gal-V), 99.15 (C1-GalNAc-IV), 99.10 (C1-Gal-III), 81.78, 81.60, 81.03, 79.68, 79.53, 75.81, 75.62, 75.17, 75.12, 75.07, 74.90, 74.61, 74.06, 73.72, 73.44, 72.78, 71.64, 70.85, 70.55, 70.37, 70.06, 70.02, 68.68, 68.56, 68.04, 67.83, 66.74, 66.64, 64.46, 61.77, 61.38, 60.85, 60.41, 54.72 (C2-GalNAc-IV), 32.43 (CH_2-octadec), 31.94-21.07 (CH_2-octadec, $(CH_3)_3C$-*t*-Bu), 20.73 (CH_3CO), 20.67 (CH_3CO), 20.61(CH_3CO), 20.51 (CH_3CO), 20.45 (CH_3CO), 20.40 (CH_3CO), 14.22, 14.14 (CH_3-octadec); HRMS (ESI) calcd for $C_{150}H_{170}F_3N_4NaO_{35}Si$ $[M+Na+H]^{2+}$ 1347.5627, found 1347.8843.

***β-D-Galactopyranosyl-(1→3)-2-acetamido-2-deoxy-β-D-galactopyranosyl-(1→3)-α-D-galactopyranosyl-(1→4)-(2,3-di-O-(2-naphthyl)methyl-6-benzoyl-β-D-galactopyranosyl)-(1→4)-(2,3,6-tri-O-(2-naphthyl)methyl-β-D-glucopyranosyl)-(1→1)-(2S, 3R, 4E)-2-azido-3-O-(2-naphthyl)methyl-octadec-4-ene* (19)**

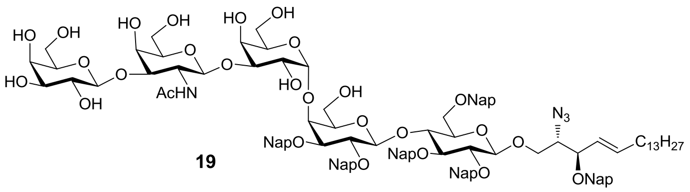

19

Compound **8** (66 mg, 0.024 mmol) was dissolved in pyridine (1 mL), and the mixture was cooled to 0 °C, followed by the addition of HF/Pyridine (65−70%, 0.1 mL). The reaction mixture was warmed to room temperature and stirred for 1 h. TLC analysis showed that the starting material disappeared. The reaction mixture was diluted with ethyl acetate (6 mL) and washed with saturated aqueous solution of $NaHCO_3$ (3 mL). The aqueous phase was extracted with EtOAc (2 × 6 mL). The combined organic phase was washed with saturated aqueous solution of $CuSO_4$ (6 mL), saturated aqueous solution of $NaHCO_3$ (6 mL), and brine (6 mL) in sequence. The organic phase was dried over anhydrous Na_2SO_4 and filtered. The filtrate was concentrated *in vacuo* to give a residue. The resulting residue was purified by silica gel column chromatography (acetone in toluene 5% →15%) to afford a desilylation intermediate for next step.

The resulting intermediate was dissolved in 1,4-dioxane (1.2 mL), MeOH (0.4 mL), and NaOH (1 M, 0.4 mL). The reaction mixture was stirred at room temperature for 8 h. ESI-MS analysis showed that the starting material disappeared. Subsequently, MeOH (1 mL) was added, and the pH of the reaction mixture was neutralized to ~7 by Amberlite IR120 hydrogen form resin. The mixture was filtered and the filtrate was concentrated under reduced pressure to give an amine intermediate for next selective acylation step.

To a solution of the resulting amine intermediate in MeOH (1 mL) and H_2O (0.25 mL) was added triethylamine (8.3 μL, 0.06 mmol) and acetic anhydride (2.4 μL, 0.026 mmol). After the reaction mixture was stirred for 2.5 h at room temperature, TLC analysis showed complete conversion of the starting material to a major product (ethyl acetate/methanol, 1:1, $R_f = 0.48$). The reaction mixture was concentrated *in vacuum* to give a residue, which was purified by Sephadex LH-20 using CH_3OH : DCM (1:1) as eluent to afford compound **19** as a white amorphous solid (39 mg, 81%). ^1H NMR (500 MHz, $CDCl_3$: CD_3OD 1:1): δ 7.82 – 7.56 (m, 23H, H-Ar), 7.50 – 7.28 (m, 17H, H-Ar), 7.25-7.18 (m, 2H, H-Ar), 5.65 – 5.53 (m, 1H, -CH=*CH*-), 5.40 – 5.33 (m, 1H, -CH=*CH*-), 5.18 (d, *J* = 11.1 Hz, 1H, *CH*H-Nap), 5.07 (d, *J* =

3.8 Hz, 1H, H1-Gal-III), 4.96 (d, *J* = 11.4 Hz, 1H, *CH*H-Nap), 4.90 – 4.80 (m, 4H), 4.67-4.63 (m, 2H), 4.57-4.53 (m, 2H), 4.46 – 4.39 (m, 2H, H1-Gal-II, *CH*H-Nap), 4.37 – 4.32 (m, 2H, *CH*H-Nap, H1-Glc-I), 4.25 (d, *J* = 8.4 Hz, 1H, H1-GalNAc-IV), 4.18–4.14 (m, 1H), 4.08 (d, *J* = 2.9 Hz, 1H, H4-Gal-II), 4.02 (d, *J* = 7.4 Hz, 1H, H1-Gal-V), 4.00 – 3.65 (m, 18H), 3.64 – 3.56 (m, 4H), 3.51 – 3.40 (m, 5H), 3.35 (dd, *J* = 9.2, 7.7 Hz, 1H, H2-Glc-I), 3.28 – 3.26 (m, 1H), 3.20 (dd, *J* = 10.0, 2.8 Hz, 1H, H3-Gal-II), 3.08 – 3.02 (m, 1H), 2.71 (t, *J* = 5.9 Hz, 1H), 2.02-1.94 (m, 2H, CH_2-octadec), 1.80 (s, 3H, CH_3CONH), 1.27 – 1.15 (m, 22H, CH_2-octadec), 0.83 (t, *J* = 7.0 Hz, 3H, CH_3-octadec); ^{13}C NMR (125 MHz, $CDCl_3$: CD_3OD 1:1): δ 173.23 (NH*CO*), 138.05 (-CH=*C*H-), 135.71–125.09 (*C*-Ar), 124.97 (-*C*H=CH-), 104.41 (C1-Gal-V), 102.83 (C1-Glc-I), 102.43 (C1-Gal-II), 101.66 (C1-GalNAc-IV), 100.55 (C1-Gal-III), 81.83, 80.70, 80.59, 79.81, 79.33, 79.08, 78.84, 76.15, 74.73, 74.58, 74.49, 74.39, 74.36, 74.15, 73.42, 72.69, 72.57, 71.64 (C2-Gal-V), 70.53, 70.05, 69.37, 68.85, 68.29, 67.90, 67.88, 67.39, 67.20, 63.82, 61.28, 60.79, 60.62, 59.85, 51.51, 48.61, 31.71 (CH_2-octadec), 31.30 (CH_2-octadec), 29.05, 29.02, 29.00, 28.78, 28.73, 28.45, 28.31, 22.03, 21.93, 13.19 (CH_3-octadec); HRMS (ESI) calcd for $C_{116}H_{136}N_4NaO_{27}$ $[M+Na]^+$ 2039.9284 , found 2039.9284.

***β*-D-Galactopyranosyl-(1→3)-2-acetamido-2-deoxy-*β*-D-galactopyranosyl-(1→3)-*α*-D-galactopyranosyl-(1→4)-*β*-D-galactopyranosyl-(1→4)-*β*-D-glucopyranoside-(1→1)-(2S, 3R, 4E)-2- aminooctadec-3-O-benzoyloxy-octadec-4-ene-1,3-diol** (**2**)

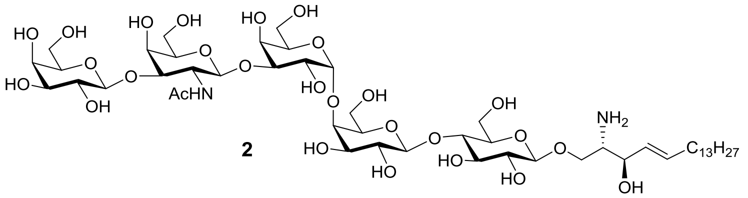

2

To a solution of compound **19** (30 mg, 0.015 mmol) in CH_2Cl_2 (1.2 mL) and H_2O (0.1 mL) was added DDQ (23 mg, 0.1 mmol) and stirred at room temperature for 3 h in the dark. Then the reaction mixture was quenched with saturated aqueous $NaHCO_3$ and concentrated *in vacuum* to give a residue. The resulting residue was purified by a C18 reverse-phase silica column, which was eluted with a gradient of CH_3OH and H_2O (CH_3OH in H_2O, 0→100%) to afford an intermediate. To a solution of the resulting intermediate in pyridine (1 mL) and H_2O (1 mL) was added 1,3-propanedithiol (15 μL, 0.15 mmol) and Et_3N (63 μL, 0.45 mmol).[12] The reaction mixture was stirred at room temperature for 12 h. ESI-MS analysis showed the

starting material disappeared. The reaction mixture was concentrated *in vacuo*. The resulting residue was purified by a C18 reverse-phase silica column, which was eluted with a gradient of CH_3OH and H_2O (CH_3OH in H_2O, 0→100%). The product containing fractions were lyophilized to afford compound **2** as a white amorphous solid (15.7 mg, 91% over two steps). ^1H NMR (800 MHz, CD_3OD): δ 5.76 (dt, J = 15.4, 6.8 Hz, 1H, -CH=*CH*-), 5.49 (dd, J = 15.4, 7.6 Hz, 1H, -*CH*=CH-), 4.94 (d, J = 4.0 Hz, 1H, H1-Gal-III), 4.71 (d, J = 8.4 Hz, 1H, H1-GalNAc-IV), 4.41 (d, J = 7.7 Hz, 1H, H1-Gal-II), 4.35 (d, J = 7.6 Hz, 1H, H1-Gal-V), 4.31 (d, J = 7.8 Hz, 1H, H1-Glc-I), 4.28 – 4.25 (m, 1H), 4.17 (dd, J = 3.1, 1.3 Hz, 1H), 4.11 (d, J = 3.1 Hz, 1H), 4.07 (dd, J = 10.8, 8.4 Hz, 1H, H2-GalNAc-IV), 4.01 – 3.97 (m, 2H, H4-Gal-II, CH_2-octadec), 3.94 (dd, J = 10.2, 3.9 Hz, 1H, H2-Gal-III), 3.92 – 3.86 (m, 2H), 3.86 – 3.83 (m, 4H), 3.82 – 3.80 (m, 2H), 3.79 – 3.67 (m, 8H), 3.58 – 3.50 (m, 7H), 3.45 (dd, J = 9.7, 3.3 Hz, 1H), 3.47 – 3.44 (m, 1H), 3.28 (dd, J = 9.1, 7.8 Hz, 1H, H2-Glc-I), 2.94 – 2.90 (m, 1H), 2.11 – 2.07 (m, 2H, CH_2-octadec), 1.99 (s, 3H, CH_3CONH), 1.44 – 1.40 (m, 2H, CH_2-octadec), 1.32 – 1.26 (m, 22H, CH_2-octadec), 0.90 (t, J = 7.1 Hz, 3H, CH_3-octadec); HRMS (ESI) calcd for $C_{50}H_{91}N_2O_{27}$ $[M+H]^+$ 1151.5804 , found 1151.5805.

7. Synthesis of Gb5 glycolipid 20

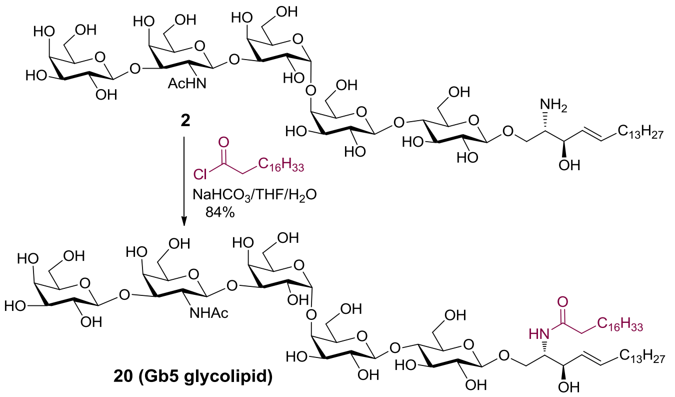

β-D-Galactopyranosyl-(1→3)-2-acetamido-2-deoxy-β-D-galactopyranosyl-(1→3)-α-D-galactopyranosyl-(1→4)-β-D-galactopyranosyl-(1→4)-β-D-glucopyranoside-(1→1)-(2S, 3R, 4E)-2-(hexadecaneacetamido)-octadec-4-ene-1, 3-diol **(20, Gb5 glycolipid)**

To a solution of compound **2** (2.1 mg, 0.002 mmol) in H_2O (300 μL), THF (100 μL) and saturated aqueous $NaHCO_3$ (100 μL) was added stearoyl chloride (1.6 mg, 0.006 mmol) at 0 °C. The reaction mixture was warmed to room temperature and stirred for 30 min. After TLC analysis showed complete conversion of **2** to a major product (EtOAc/MeOH/H_2O =

5/2/1, R_f = 0.7), the mixture was concentrated under reduced pressure to give a residue. The resulting residue was purified by Sephadex LH-20 size exclusion chromatography using CH_3OH as eluent to afford compound **20** (2.1 mg, 84%). ^{1}H NMR (600 MHz, CD_3OD): δ 5.70 (dt, J = 14.2, 6.7 Hz, 1H, -CH=*CH*-), 5.49-5.43 (m, 1H, -*CH*=CH-), 4.95 (d, J = 4.0 Hz, 1H, H1-Gal-III), 4.72 (d, J = 8.4 Hz, 1H, H1-GalNAc-IV), 4.43 – 4.41 (m, 1H, H1-Gal-II), 4.36 (d, J = 7.6 Hz, 1H, H1-Gal-V), 4.32 (d, J = 7.8 Hz, 1H, H1-Glc-I), 4.29 – 4.25 (m, 1H), 4.20 – 4.16 (m, 2H), 4.12 (d, J = 3.1 Hz, 1H), 4.10 – 4.05(m, 2H), 4.02 – 3.99 (m, 1H), 3.95 (dd, J = 10.2, 3.9 Hz, 1H, H2-Gal-III), 3.93 – 3.89 (m, 2H), 3.87 – 3.84 (m, 3H), 3.83 – 3.80 (m, 2H), 3.79 – 3.67 (m, 8H), 3.62 – 3.59 (m, 1H), 3.59 – 3.51 (m, 7H), 3.46 (dd, J = 9.7, 3.3 Hz, 1H), 3.44 – 3.41 (m, 1H), 3.29 (m, 1H, H2-Glc-I), 2.19 (t, J = 7.6 Hz, 2H), 2.06 – 2.02 (m, 2H, *CH_2*-octadec), 2.00 (s, 3H, *CH_3*CONH), 1.62 – 1.58 (m, 2H), 1.41 – 1.37 (m, 2H, *CH_2*-octadec), 1.33 – 1.29 (m, 48H, *CH_2*-octadec), 0.91 (t, J = 7.0 Hz, 6H, *CH_3*-octadec); HRMS (ESI) calcd for $C_{68}H_{125}N_2O_{28}$ $[M+H]^+$ 1417.8413 , found 1417.8377.

8. Chemoenzymatic synthesis of DSGb5 glycolipid 1

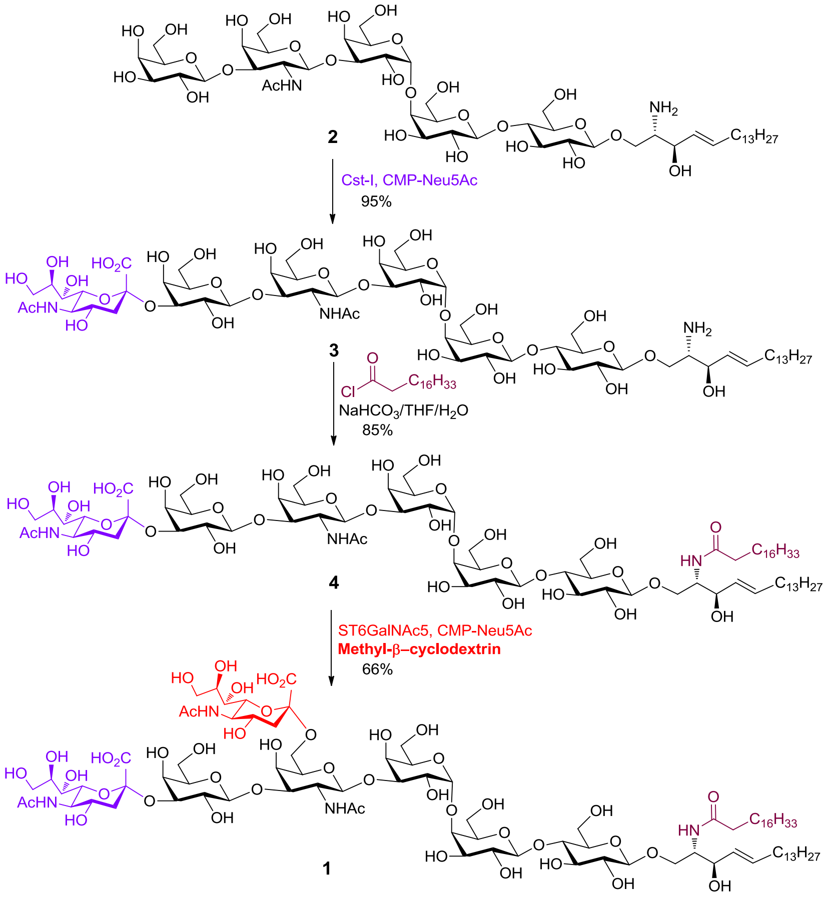

Scheme S4. Synthesis of DSGb5 glycolipid **1**

***(5-Acetamido-3,5-dideoxy-D-glycero-α-D-galacto-2-nonulopyranosylonic acid)-(2→3)-(β-D-galactopyranosyl)-(1→3)-(2-acetamido-2-deoxy-β-D-glucopyranosyl)-(1→3)-(α-D-galactopyranosyl)-(1→4)-(β-D-galactopyranosyl)-(1→4)-(β-D-glucopyranosyl)-(1→1)-(2S, 3R, 4E)-2-aminooctadec-octadec-4-ene-1, 3-diol* (3)**

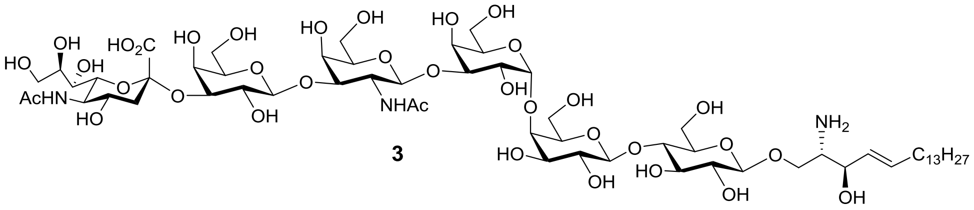

3

To a 350 μL solution of compound **2** (8 mg, 0.007 mmol), CMP-Neu5Ac (6.7 mg, 0.0105 mmol) and $MnCl_2$ (10 mM) in HEPES buffer (100 mM, pH 7.2) was added Cst-I (70 μg), and the reaction mixture was incubated for 40 min at 37 °C.[2] ESI-MS analysis showed the starting material **2** disappeared. The reaction mixture was centrifuged, and the resulting supernatant was loaded on a C18 reverse-phase silica column (eluent: H_2O containing 1% HCOOH to 100% MeOH). Product containing fractions were combined and concentrated for lyophilization to afford compound **3** as a white amorphous solid (7.6 mg, 95%). ^{1}H NMR (800 MHz, CD_3OD): δ 5.82 (dt, J = 15.4, 6.8 Hz, 1H, -CH=*CH*-), 5.49 (dd, J = 15.4, 7.1 Hz, 1H, -*CH*=CH-), 4.94 (d, J = 3.9 Hz, 1H, H1-Gal-III), 4.73 (d, J = 8.6 Hz, 1H, H1-GalNAc-IV), 4.44 – 4.41 (m, 2H, H1-Gal-II, H1-Gal-V), 4.35 (d, J = 7.9 Hz, 1H, H1-Glc-I), 4.28 – 4.24 (m, 1H), 4.18 – 4.16 (m, 2H, *CH$_2$*-octadec), 4.12 (d, J = 3.0 Hz, 1H), 4.05 – 3.98 (m, 3H), 3.95-3.91 (m, 3H), 3.90 (d, J = 3.5 Hz, 1H), 3.89 (d, J = 4.5 Hz, 1H), 3.87 (d, J = 4.4 Hz, 1H), 3.86 – 3.84 (m, 5H), 3.81 (dd, J = 11.5, 2.5 Hz, 1H), 3.79 – 3.77 (m, 1H), 3.76 – 3.66 (m, 10H), 3.64 – 3.60 (m, 2H), 3.59 – 3.55 (m, 4H), 3.53 (d, J = 8.9 Hz, 1H), 3.50 (d, J = 9.9 Hz, 1H), 3.45 (d, J = 8.8 Hz, 1H), 3.30 – 3.29 (dd, J = 9.1, 7.8 Hz, 1H), 3.23 – 3.17 (m, 1H), 2.87 (dd, J = 12.3 Hz, 3.6 Hz, 1H, $H3_{(eq)}$-Neu5Ac-VI), 2.11 – 2.09 (m, 2H, *CH$_2$*-octadec), 2.01 (s, 6H, 2 x *CH$_3$*CONH), 1.73 (t, J = 12.3 Hz, 1H, $H3_{(ax)}$-Neu5Ac-VI), 1.44 – 1.41 (m, 2H, *CH$_2$*-octadec), 1.31 – 1.28 (m, 20H, *CH$_2$*-octadec), 0.90 (t, J = 7.1 Hz, 3H, *CH$_3$*-octadec); HRMS (ESI) calcd for $C_{61}H_{108}N_3O_{35}$ $[M+H]^+$ 1442.6758, found 1442.6756.

Table S2. Different α2,3-sialyltransferases for conversion Gb5-sphingosine 2 to SSEA-4 sphingosine 3

Entry	Enzyme	Yield (%)
1	ST3Gal1	0
2	PmST1 M144D	< 30
3	Cst-I	95

Gb5-sphingosine **2** was treated with a mammalian α2,3-sialyltransferase ST3Gal1 expressed from HEK293 cells,[6] *Pasteurella multocida* α2,3-sialyltransferase 1 M144D mutant (PmST1 M144D) expressed from *E.coli*,[1] and *Campylobacter jejuni* α2,3-sialyltransferase (Cst-I)[2] expressed from *E.coli* for sialylation in the presence of CMP-Neu5Ac, respectively. The results indicated that **2** was sialylated to afford SSEA-4 sphingosine **3** by Cst-I in a high yield of 95% and PmST1 M144D in a moderate yield (<30%), but the treatment of **2** with ST3Gal1 and CMP-Neu5Ac did not generate any product by electrospray ionization-mass spectrometry (ESI-MS) analysis.

(5-Acetamido-3,5-dideoxy-D-glycero-α-D-galacto-2-nonulopyranosylonic acid)-(2→3)-(β-D-galactopyranosyl)-(1→3)-(2-acetamido-2-deoxy-β-D-glucopyranosyl)-(1→3)-(α-D-galactopyranosyl)-(1→4)-(β-D-galactopyranosyl)-(1→4)-(β-D-glucopyranosyl)-(1→1)-(2S, 3R, 4E)-2-(hexadecaneacetamido)-octadec-4-ene-1, 3-diol (**4**)

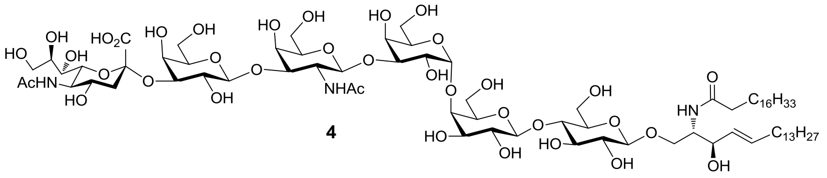

4

To a solution of compound **3** (6 mg, 0.005 mmol) in H_2O (600 μL), THF (200 μL), and saturated aqueous $NaHCO_3$ (200 μL) was added stearoyl chloride (4.5 mg, 0.015 mmol) at 0 °C. The reaction mixture was warmed to room temperature and stirred for 30 min. After TLC analysis showed complete conversion of **3** to a major product (EtOAc/MeOH/H_2O = 5/2/1, R_f = 0.5), the mixture was concentrated under reduced pressure to give a residue. The resulting residue was purified by Sephadex LH-20 size exclusion chromatography using CH_3OH as eluent to afford compound **4** (7.3 mg, 85%). ^{1}H NMR (500 MHz, CD_3OD): δ 5.68 (dt, J = 15.4, 6.7 Hz, 1H, -CH=*CH*-), 5.44 (dd, J = 15.4, 7.5 Hz, -CH=*CH*-), 4.94 (d, J = 3.9 Hz, 1H, H1-Gal-III), 4.71 (d, J = 8.4 Hz, 1H, H1-GalNAc-IV), 4.42 (d, J = 7.8 Hz, 1H, H1-

Gal-II), 4.40 (d, *J* = 7.6 Hz, 1H, H1-Gal-V), 4.31 (d, *J* = 7.8 Hz, 1H, H1-Glc-I), 4.27 – 4.23 (m, 1H), 4.19 – 4.14 (m, 2H), 4.12 – 4.10 (m, 1H), 4.09 – 4.04 (m, 2H), 4.02 (dd, *J* = 9.7, 3.2 Hz, 1H), 3.99 – 3.95 (m, 2H), 3.93 (dd, *J* = 10.2, 3.9 Hz, 1H, H2-Gal-III), 3.90 – 3.87 (m, 3H), 3.86 – 3.79 (m, 6H), 3.77 – 3.64 (m, 10H), 3.64 – 3.52 (m, 10H), 3.51 – 3.48 (m, 2H), 3.43 – 3.40 (m, 1H), 3.27 (dd, *J* = 9.1, 7.8 Hz, 1H), 2.86 (dd, *J* = 11.3, 3.1 Hz, 1H, $H3_{(eq)}$-Neu5Ac-VI), 2.19 – 2.15 (m, 2H, NHCO-CH_2-), 2.05 – 2.01 (m, 2H), 2.00 (s, 6H, 2 x CH_3CONH), 1.71 (t, *J* = 11.3 Hz, 1H, $H3_{(ax)}$-Neu5Ac-VI), 1.61 – 1.55 (m, 2H), 1.40 – 1.36 (m, 2H), 1.31 – 1.27 (m, 48H), 0.89 (t, *J* = 6.8 Hz, 6H, 2 x CH_3-); HRMS (ESI) calcd for $C_{79}H_{140}N_3O_{36}$ $[M-H]^-$ 1706.9222, found 1706.9224.

(5-Acetamido-3,5-dideoxy-D-glycero-α-D-galacto-2-nonulopyranosylonic acid)-(2→3)-(β-D-galactopyranosyl)-(1→3)-[(5-acetamido-3,5-dideoxy-D-glycero-α-D-galacto-2-nonulo-pyranosylonic acid)-(2→6)]-2-acetamido-2-deoxy-β-D-glucopyranosyl)-(1→3)-(α-D-galactopyranosyl)-(1→4)-(β-D-galactopyranosyl)-(1→4)-(β-D-glucopyranosyl)-(1→1)-(2S, 3R, 4E)-2-(hexadecaneacetamido)-octadec-4-ene-1, 3-diol **(1)**

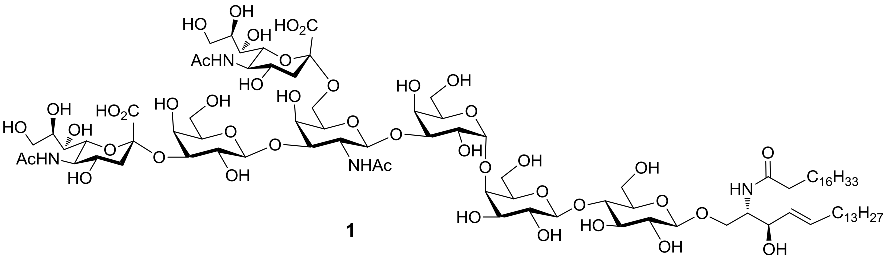

1

A mixture of compound **4** (5 mg, 0.003 mmol) and methy-β-cyclodextrin (3.3 mg, 0.003 mmol) in 200 μL Tris-HCl buffer (100 mM, pH 7.0) was sonicated for 30 min for the preparation of an inclusion complex.[13] To a solution of the resulting inclusion complex was added CMP-Neu5Ac (3.8 mg, 0.006 mmol), $MgCl_2$ (10 mM), ST6GalNAc5 (60 μg) and calf intestine alkaline phosphatase (CIAP, 2 U), and the mixture was incubated for 16 h at 37 °C.[6] Subsequently, ESI-MS analysis was performed to monitor the reaction until no starting material was detected. The reaction mixture was centrifuged, and the resulting supernatant was loaded on a C18 reverse-phase silica column eluted by a gradient from 100% H_2O containing 0.1% HCOOH to 100% mixture solution of *i*-PrOH/CH_3OH/CH_3CN (75:25:5) to give a crude, which was further purified by HW-40F size exclusion chromatography using

CH_3OH as eluent to afford compound **1** (4 mg, 66%). ^1H NMR (600 MHz, CD_3OD): δ 5.68 (dt, *J* = 15.4, 6.7 Hz, 1H, -CH=*CH*-), 5.45 (dd, *J* = 15.4, 7.5 Hz, 1H, -*CH*=CH-), 4.94 (d, *J* = 3.6 Hz, 1H, H1-Gal-III), 4.66 (d, *J* = 8.5 Hz, 1H, H1-GalNAc-IV), 4.43 (d, J = 7.8 Hz, 1H, H1-Gal-II) 4.40 (d, J = 7.4 Hz, 1H, H1-Gal-V), 4.35-4.31 (m, 2H, H1-Glc-I), 4.22 – 4.18 (m, 2H), 4.13 (d, *J* = 3.1 Hz, 1H, H4-GalNAc-IV), 4.10 – 4.05 (m, 2H), 4.04 – 3.96 (m, 4H), 3.95 – 3.92 (m, 2H), 3.91-3.88 (m, 4H), 3.87 – 3.83 (m, 5H), 3.82-3.78 (m, 2H), 3.76 – 3.66 (m, 10H), 3.65 – 3.53 (m, 10H), 3.52 – 3.42 (m, 4H), 3.29 (dd, *J* = 9.1, 7.8 Hz, 1H), 2.89 – 2.80 (m, 2H, $H3_{(eq)}$-Neu5Ac-VI, $H3_{(eq)}$-Neu5Ac-VII), 2.23 – 2.18 (m, 2H, 2-NHCO-CH_2-), 2.05 – 2.01 (m, 2H), 2.00 (s, 9H, 3 x CH_3CONH), 1.72 (t, *J* = 11.5 Hz, 1H, $H3_{(ax)}$-Neu5Ac-VI), 1.63 – 1.56 (m, 3H, $H3_{(ax)}$-Neu5Ac-VII, CH_2-lipid), 1.41 – 1.37 (m, 2H), 1.32 – 1.28 (m, 48H), 0.90 (t, *J* = 6.8 Hz, 6H, 2 x CH_3-); HRMS (ESI) calcd for $C_{90}H_{156}N_4O_{44}$ $[M-2H]^{2-}$ 998.5052 , found 998.5052.

9. Enzymatic synthesis of DSGb5 glycan

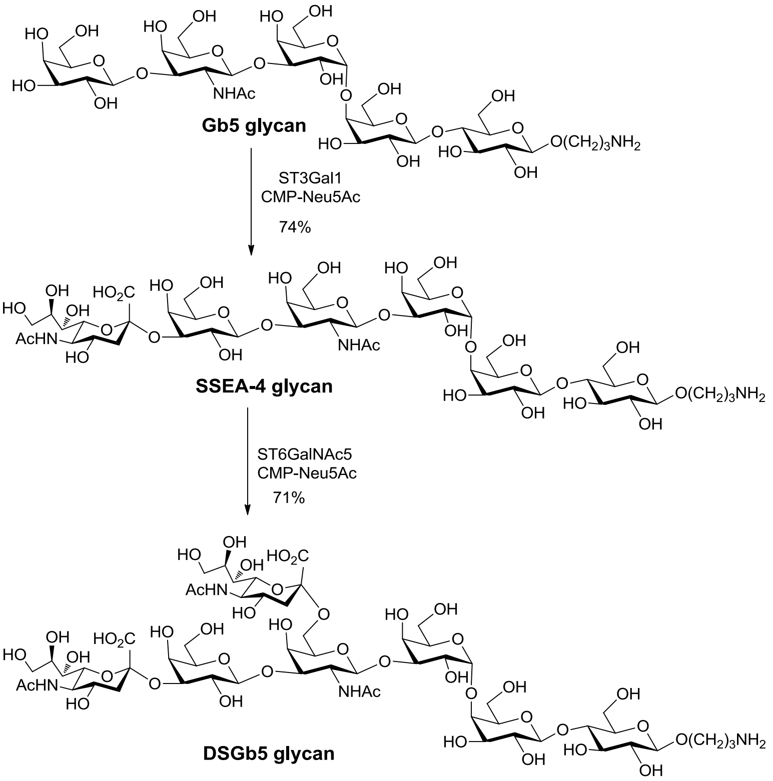

Scheme S5. Enzymatic synthesis of DSGb5 glycan

3-Aminopropyl (5-acetamido-3,5-dideoxy-D-glycero-α-D-galacto-2-nonulopyranosylonic acid)-(2→3)-(β-D-galactopyranosyl)-(1→3)-(2-acetamido-2-deoxy-β-D-glucopyranosyl)-(1

***→3)-(α-D-galactopyranosyl)-(1→4)-(β-D-galactopyranosyl)-β-D-glucopyranoside* (SSEA-4 glycan)**

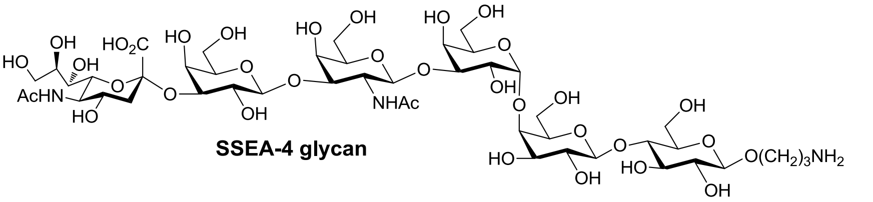

To a 300 μL solution of Gb5 glycan[14] (5.6 mg, 0.006 mmol), CMP-Neu5Ac (5.7 mg, 0.009 mmol) and $MgCl_2$ (10 mM) in Tris-HCl buffer (100 mM, pH 7.0) was added ST3Gal1 (30 μg), and the mixture was incubated for 16 h at 37 °C.[6] Subsequently, ESI-MS analysis was performed to monitor the reaction until no starting material was detected. The reaction mixture was centrifuged, and the resulting supernatant was purified by Bio-Gel P-4 size exclusion chromatography. Product-containing fractions were combined and lyophilized to give the SSEA-4 glycan as a white amorphous solid (5.4 mg, 74%). ^{1}H NMR (600 MHz, D_2O): δ 4.87 (d, J = 4.0 Hz, 1H, H1-Gal-III), 4.65 (d, J = 8.6 Hz, 1H, H1-GalNAc-IV), 4.49 – 4.45 (m, 3H, H1-Gal-II, H1-Glc-I, H1-Gal-V), 4.35 (t, J = 6.4 Hz, 1H), 4.22 – 4.20 (m, 1H), 4.13 (d, J = 3.2 Hz, 1H), 4.05 – 3.98 (m, 4H), 3.97 – 3.91 (m, 2H), 3.91 – 3.86 (m, 4H), 3.85 – 3.84 (m, 1H), 3.82 – 3.73 (m, 8H), 3.71-3.69 (m, 2H), 3.68 – 3.63 (m, 5H), 3.62 – 3.57 (m, 6H), 3.56 – 3.54 (m, 2H), 3.52 – 3.49 (m, 1H), 3.30 – 3.26 (m, 1H), 3.13 – 3.10 (m, 2H), 2.71 (dd, J = 12.5, 4.6 Hz, 1H, $H3_{(eq)}$-Neu5Ac-VI), 2.01 – 1.94 (m, 8H, 2 x CH_3CONH, -CH_2-*aminopropyl*), 1.74 (t, J = 12.5 Hz, 1H, $H3_{(ax)}$-Neu5Ac-VI); ESI HRMS (m/z): $[M + H]^+$ calcd for $C_{46}H_{80}N_3O_{34}$, 1218.4618; found 1218.4621.

***3-Aminopropyl (5-acetamido-3,5-dideoxy-D-glycero-α-D-galacto-2-nonulopyranosylonic acid)-(2→3)-(β-D-galactopyranosyl)-(1→3)-[(5-acetamido-3,5-dideoxy-D-glycero-α-D-galacto-2-nonulopyranosylonic acid)-(2→6)]-2-acetamido-2-deoxy-β-D-glucopyranosyl)-(1→3)-(α-D-galactopyranosyl)-(1→4)-(β-D-galactopyranosyl)-(1→4)-β-D-glucopyranoside* (DSGb5 glycan)**

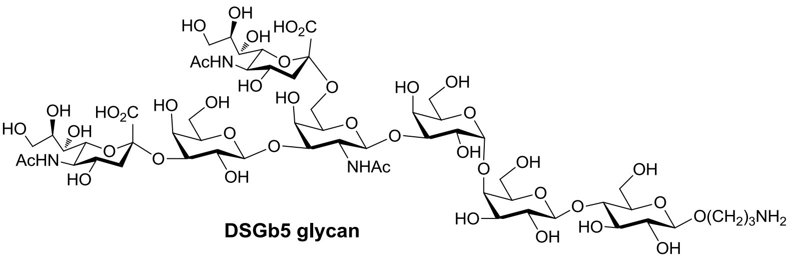

To a 200 μL solution of SSEA4 glycan (5 mg, 0.004 mmol), CMP-Neu5Ac (3.8 mg, 0.006 mmol) and $MgCl_2$ (10 mM) in Tris-HCl buffer (100 mM, pH 7.0) was added ST6GalNAc5 (40 μg) and calf intestine alkaline phosphatase (CIAP, 2 U).[6] The reaction mixture was incubated for 16 h at 37 °C. Subsequently, ESI-MS analysis was performed to monitor the reaction until no starting material was detected. The reaction mixture was centrifuged, and the resulting supernatant was purified by Bio-Gel P-4 size exclusion chromatography. Product-containing fractions were combined and lyophilized to give the DSGb5 glycan as a white amorphous solid (4.3 mg, 71%). ^{1}H NMR (600 MHz, D_2O): δ 4.86 (d, J = 4.1 Hz, 1H, H1-Gal-III), 4.63 (d, J = 8.5 Hz, 1H, H1-GalNAc-IV), 4.51 – 4.44 (m, 3H, H1-Gal-II, H1-Glc-I, H1-Gal-V), 4.36 (t, J = 6.5 Hz, 1H), 4.22 (d, J = 3.9 Hz, 1H), 4.15 (d, J = 3.3 Hz, 1H), 4.04 – 3.99 (m, 4H), 3.98 – 3.93 (m, 2H), 3.91 – 3.86 (m, 4H), 3.85 – 3.81 (m, 5H), 3.80-3.77 (m, 4H), 3.76 (d, J = 4.3 Hz, 1H), 3.74 – 3.67 (m, 5H), 3.66 –3.56 (m, 14H), 3.56 – 3.53 (m, 2H), 3.50 (dd, J = 9.8, 7.8 Hz, 1H), 3.30 – 3.26 (m, 1H), 3.14-3.10 (m, 2H), 2.75 – 2.65 (m, 2H, $H3_{(eq)}$-Neu5Ac-VI, $H3_{(eq)}$-Neu5Ac-VII), 2.00 – 1.96 (m, 11H, 3 x CH_3CONH, -CH_2-*aminopropyl*), 1.74 (t, J = 12.1 Hz, 1H, $H3_{(ax)}$-Neu5Ac-VI), 1.62 (t, J = 12.1 Hz, 1H, $H3_{(ax)}$-Neu5Ac-VII); ESI HRMS (m/z): $[M + H]^+$ calcd for $C_{57}H_{97}N_4O_{42}$, 1509.5572; found 1509.5571.

10. Experimental procedure of surface plasmon resonance

Surface plasmon resonance (SPR) measurements were performed on a Biacore 8K instrument at 25 °C.[15,16] Biotinylated human Siglec-7 (ACROBiosystems, #SG7-H82E7) or biotinylated human Siglec-10 (ACROBiosystems, #SI0-H82E3) was captured on streptavidin-coated CM5 sensor chips in the flow channels until approximately 3000 response unit (RU) was achieved. Next, the reference and modified flow cells were washed with three consecutive injections of a mixture solution of 50% isopropanol and 1 M NaCl in 50 mM NaOH. A mixture solution composed of PBS (10 mM, pH 7.4), 5% DMSO and 0.05% Tween-20 was used as the running buffer for the immobilization, and kinetic studies of the interaction of Siglec-7 with DSGb5 glycolipid, DSGb5 glycan, C18 ceramide (d18:1/18:0), MSGb5 glycolipid, Gb5 glycolipid, GD3 glycolipid, Neu5Ac-α2,6GalNAc-α-PAA-biotin, respectively. Kinetic study of the interaction of Siglec-10 with DSGb5 glycolipid was also performed. Analytes were dissolved in PBS buffer (10 mM, pH 7.4) containing 5% DMSO and 0.05% Tween-20, and a flow rate of 30 μL/min was employed for association and dissociation for 120 s at a constant

temperature of 25 °C. A 45 s injection of 0.15% sodium dodecyl sulfonate (SDS) at a flow rate of 30 µL/min was used for regeneration and achieving prior baseline status. Using Biacore™ Insight Evaluation Software 4.0, the response curves of various analyte concentrations were globally fitted by a 1:1 Langmuir binding model. The black line is the fitting curve.

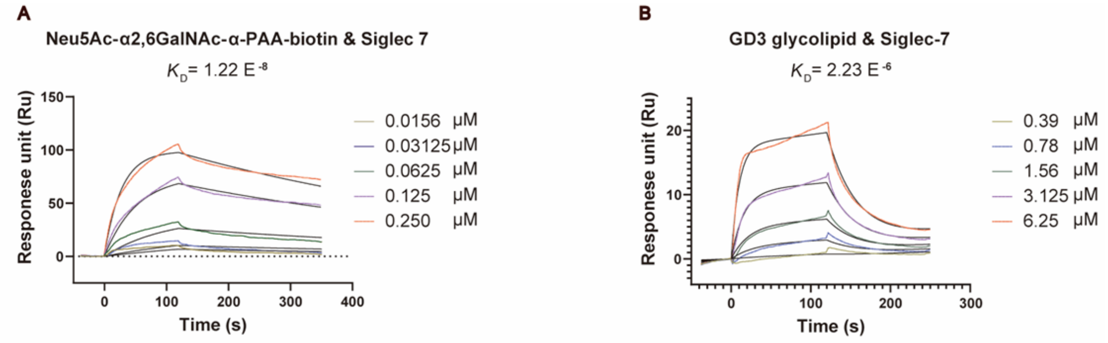

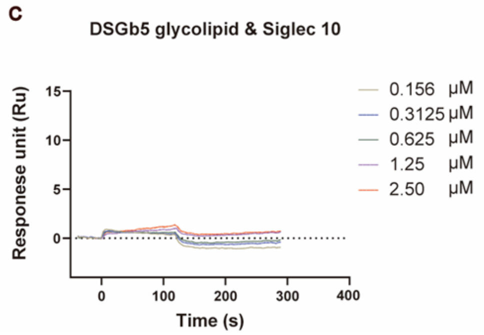

D

Compound	K_a (10^3) ($M^{-1}s^{-1}$)	K_d (10^{-3}) (s^{-1})	K_D (µM)	Chi^2
Neu5Ac-α2,6GalNAc-PAA-biotin	140	1.71	0.0122	10.6
GD3 glycolipid	12.2	27.2	2.23	0.177

Figure S4. SPR analysis of the binding affinity of Siglec-7 with Neu5Ac-α2,6GalNAc-α-PAA-biotin (A) and GD3 glycolipid (B), respectively, and the binding affinity of Siglec-10 with DSGb5 glycolipid (C) Equilibrium dissociation constants (K_D) were determined by global fitting of the binding data to a 1:1 Langmuir binding model. (D) The table of association rate constants (K_a), dissociation rate constants (K_d), K_D, and chi-square (Chi^2) goodness-of-fit values. K_D can be calculated as the ratio of K_d to K_a.

11. References

1. Sugiarto, G.; Lau, K.; Qu, J.; Li, Y.; Lim, S.; Mu, S.; Ames, J. B.; Fisher, A. J.; Chen, X. A Sialyltransferase Mutant with Decreased Donor Hydrolysis and Reduced Sialidase Activities for Directly Sialylating Lewis^x. *ACS Chem. Biol.* **2012**, *7*, 1232-1240.

2. Chiu, C. P. C.; Lairson, L. L.; Gilbert, M.; Wakarchuk, W. W.; Withers, S. G.; Strynadka, N. C. J. Structural Analysis of the α-2,3-Sialyltransferase Cst-I from *Campylobacter jejuni* in Apo and Substrate-Analogue Bound Forms. *Biochemistry* **2007**, *46*, 7196-7204.

3. Yu, H.; Thon, V.; Lau, K.; Cai, L.; Chen, Y.; Mu, S.; Li, Y.; Wang, P. G.; Chen, X. Highly efficient chemoenzymatic synthesis of β1,3-linked galactosides. *Chem. Commun.* **2010**, *46*, 7507-7509.

4. Chen, X.; Fang, J.; Zhang, J.; Liu, Z.; Shao, J.; Kowal, P.; Andreana, P.; Wang, P. G. Sugar Nucleotide Regeneration Beads (Superbeads): A Versatile Tool for the Practical Synthesis of Oligosaccharides. *J. Am. Chem. Soc.* **2001**, *123*, 2081-2082.
5. Chen, X.; Liu, Z.; Zhang, J.; Zhang, W.; Kowal, P.; Wang, P. G. Reassembled Biosynthetic Pathway for Large-Scale Carbohydrate Synthesis: α-Gal Epitope Producing "Superbug". *ChemBioChem* **2002**, *3*, 47-53.
6. 't Hart, I. M. E.; Li, T.; Wolfert, M. A.; Wang, S.; Moremen, K. W.; Boons, G.-J. Chemoenzymatic synthesis of the oligosaccharide moiety of the tumor-associated antigen disialosyl globopentaosylceramide. *Org. Biomol. Chem.* **2019**, *17*, 7304-7308.
7. Moremen, K. W.; Ramiah, A.; Stuart, M.; Steel, J.; Meng, L.; Forouhar, F.; Moniz, H. A.; Gahlay, G.; Gao, Z.; Chapla, D.; Wang, S.; Yang, J.-Y.; Prabhakar, P. K.; Johnson, R.; Rosa, M. d.; Geisler, C.; Nairn, A. V.; Seetharaman, J.; Wu, S.-C.; Tong, L.; Gilbert, H. J.; LaBaer, J.; Jarvis, D. L. Expression system for structural and functional studies of human glycosylation enzymes. *Nat. Chem. Biol.* **2018**, *14*, 156-162.
8. Liu, Y.; Ding, N.; Xiao, H.; Li, Y. Efficient Syntheses of a Series of Glycosphingolipids with 1,2-*trans*-Glycosidic Linkages. *J. Carbohydr. Chem.* **2006**, *25*, 471-489.
9. Santra, A.; Li, Y.; Yu, H.; Slack, T. J.; Wang, P. G.; Chen, X. Highly efficient chemoenzymatic synthesis and facile purification of α-Gal pentasaccharyl ceramide Galα3nLc4βCer. *Chem. Commun.* **2017**, *53*, 8280-8283.
10. Sweeney, R. P.; Lowary, T. L. A Route to Polyprenol Pyrophosphate-Based Probes of O-Polysaccharide Biosynthesis in *Klebsiella pneumoniae* O2a. *Org. Lett.* **2019**, *21*, 1050-1053.
11. Xia, J.; Abbas, S. A.; Locke, R. D.; Piskorz, C. F.; Alderfer, J. L.; Matta, K. L. Use of 1,2-dichloro 4,5-dicyanoquinone (DDQ) for cleavage of the 2-naphthylmethyl (NAP) group. *Tetrahedron Lett.* **2000**, *41*, 169-173.
12. Bayley, H.; Standring, D. N.; Knowles, J. R. Propane-1,3-dithiol: A selective reagent for the efficient reduction of alkyl and aryl azides to amines. *Tetrahedron Lett.* **1978**, *19*, 3633-3634.
13. Bungaruang, L.; Gutmann, A.; Nidetzky, B. β-Cyclodextrin Improves Solubility and Enzymatic C-Glucosylation of the Flavonoid Phloretin. *Adv. Synth. Catal.* **2016**, *358*, 486-493.
14. Wang, Z.; Gilbert, M.; Eguchi, H.; Yu, H.; Cheng, J.; Muthana, S.; Zhou, L.; Wang, P. G.; Chen, X.; Huang, X. Chemoenzymatic Syntheses of Tumor-Associated Carbohydrate Antigen Globo-H and Stage-Specific Embryonic Antigen 4. *Adv. Synth. Catal.* **2008**, *350*, 1717-1728.
15. Hu, C.; Wu, S.; He, F.; Cai, D.; Xu, Z.; Ma, W.; Liu, Y.; Wei, B.; Li, T.; Ding, K. Convergent Synthesis and Anti-Pancreatic Cancer Cell Growth Activity of a Highly Branched Heptadecasaccharide from *Carthamus tinctorius*. *Angew. Chem. Int. Ed.* **2022**, *61*, e202202554.
16. Zong, G.; Li, C.; Prabhu, S. K.; Zhang, R.; Zhang, X.; Wang, L.-X. A facile chemoenzymatic synthesis of SARS-CoV-2 glycopeptides for probing glycosylation functions. *Chem. Commun.* **2021**, *57*, 6804-6807.

12. NMR and HRMS spectra

^{1}H NMR (600 MHz, D_2O)

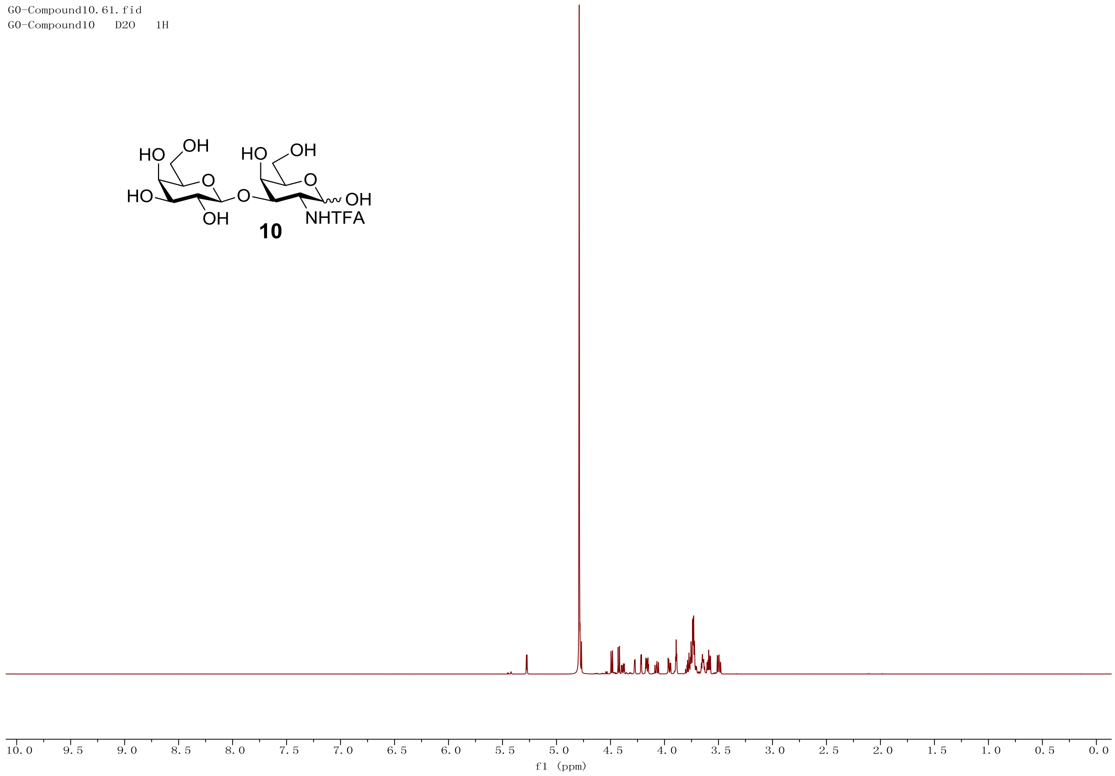

^{13}C NMR (150 MHz, D_2O)

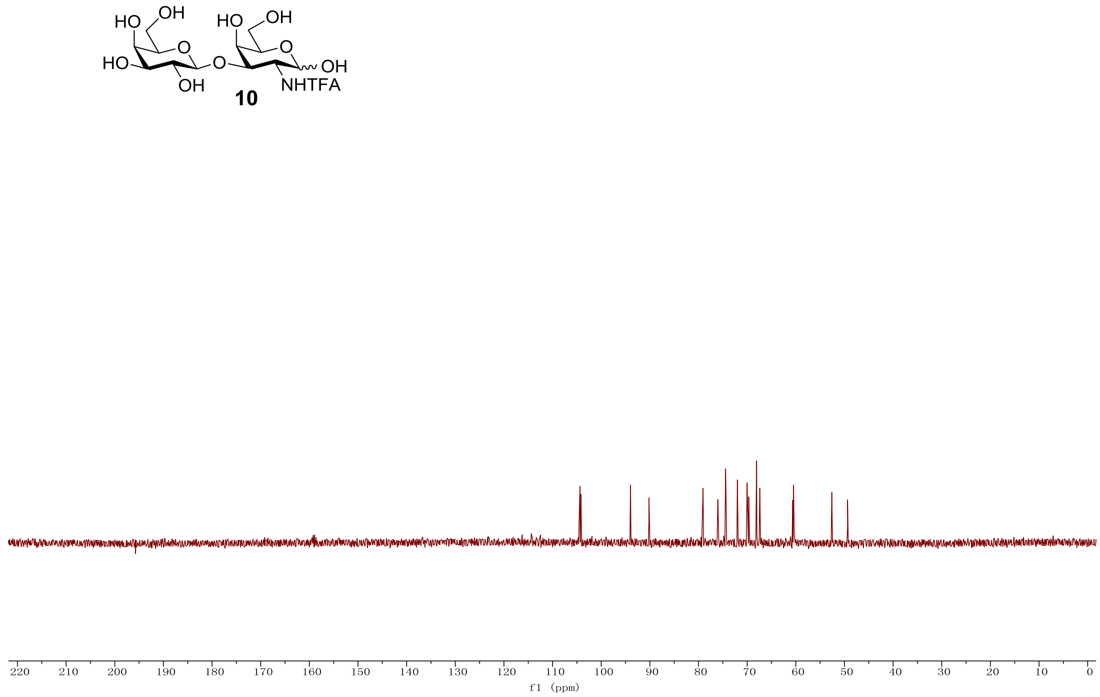

^{1}H NMR (400 MHz, $CDCl_3$)

Compound 11β

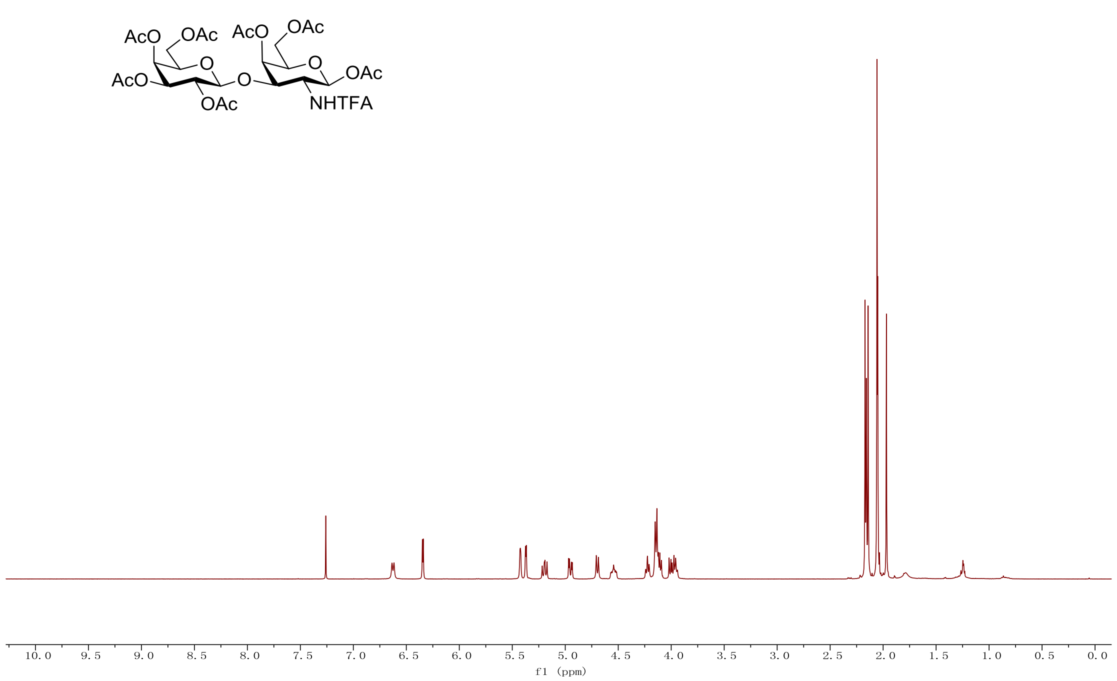

^{13}C NMR (100 MHz, $CDCl_3$)

Compound 11β

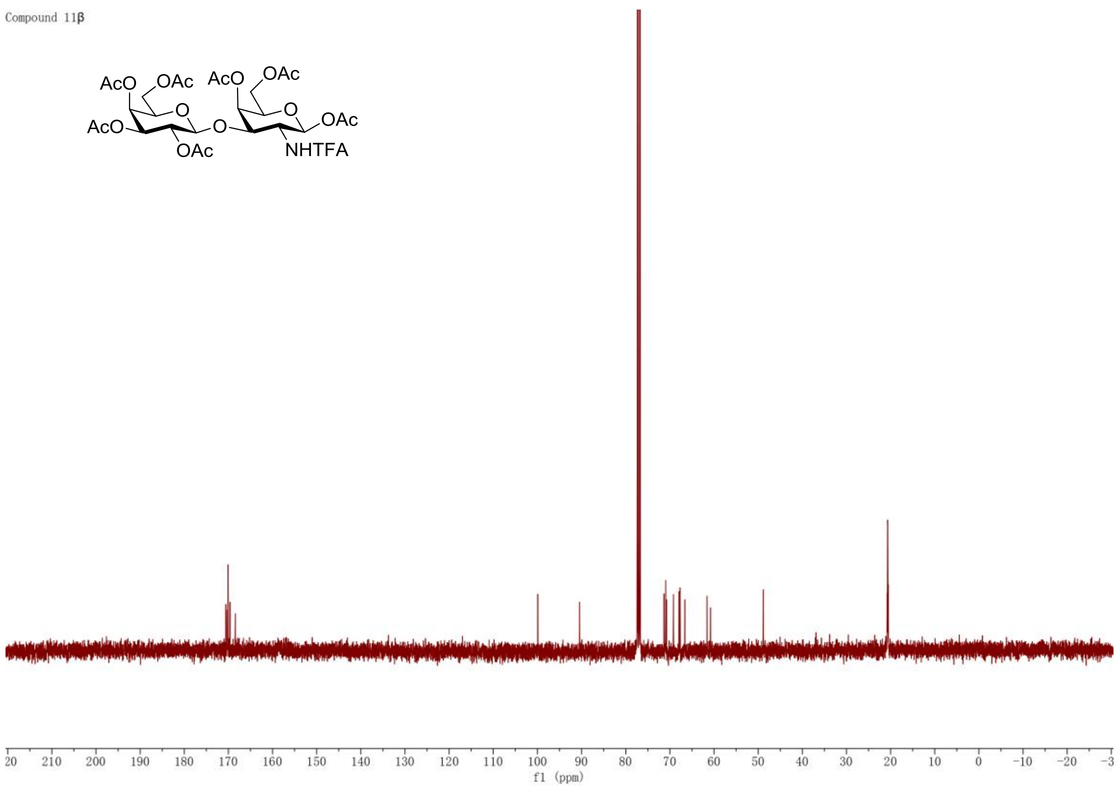

^{1}H NMR (400 MHz, $CDCl_3$)

Compound 5

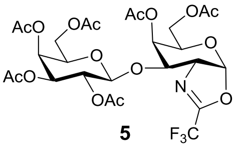

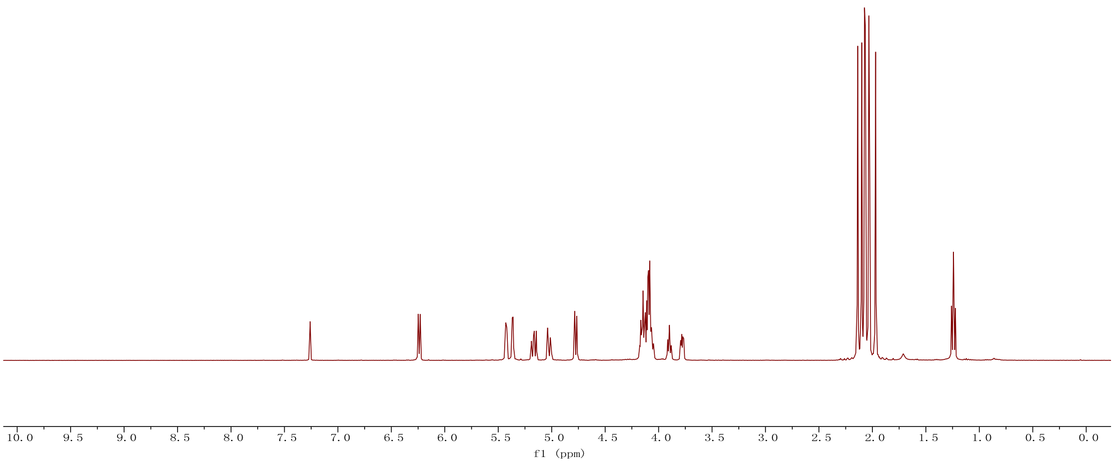

^{13}C NMR (100 MHz, $CDCl_3$)

Compound 5

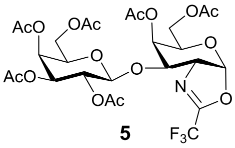

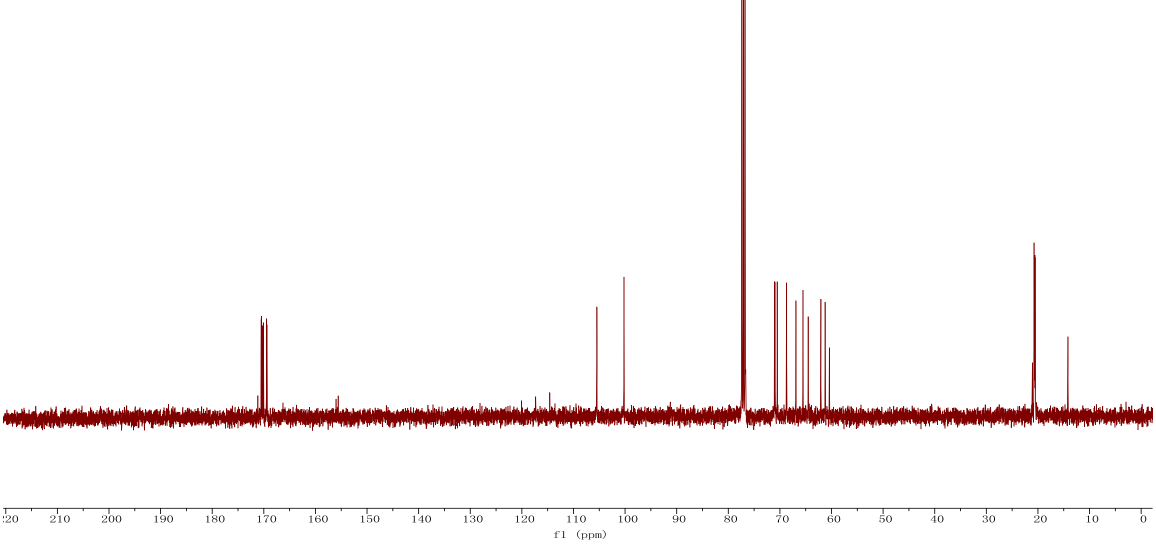

^{1}H NMR (600 MHz, $CDCl_3$)

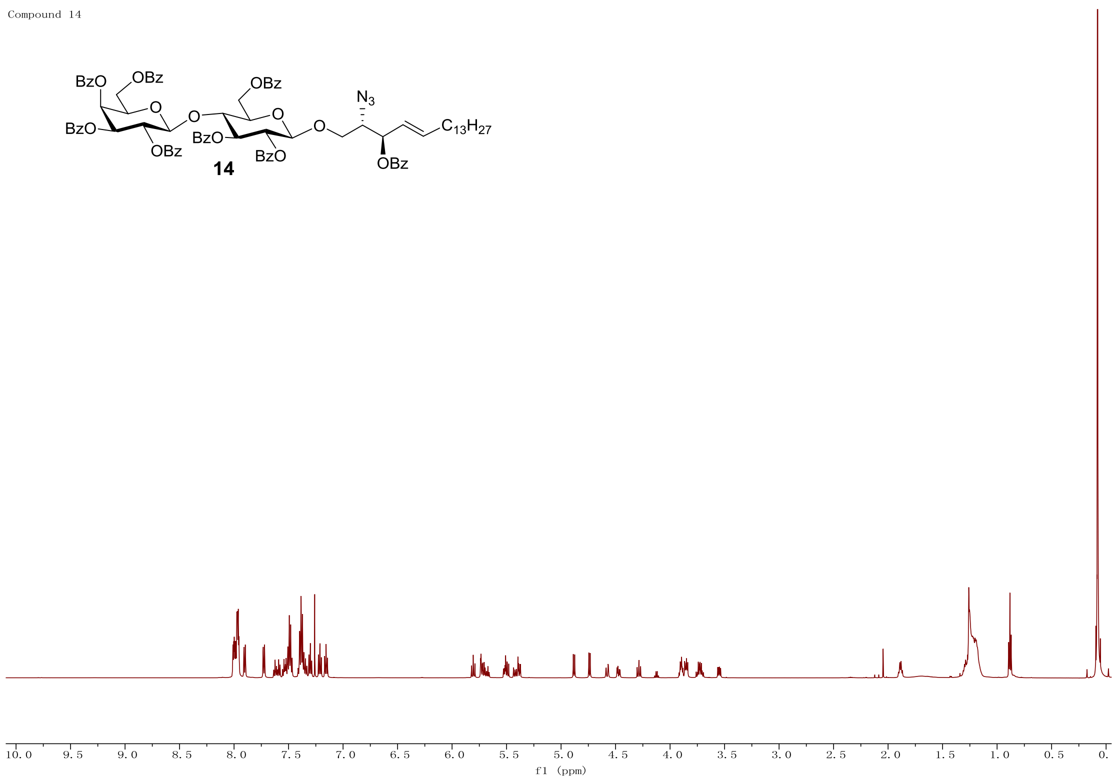

^{13}C NMR (150 MHz, $CDCl_3$)

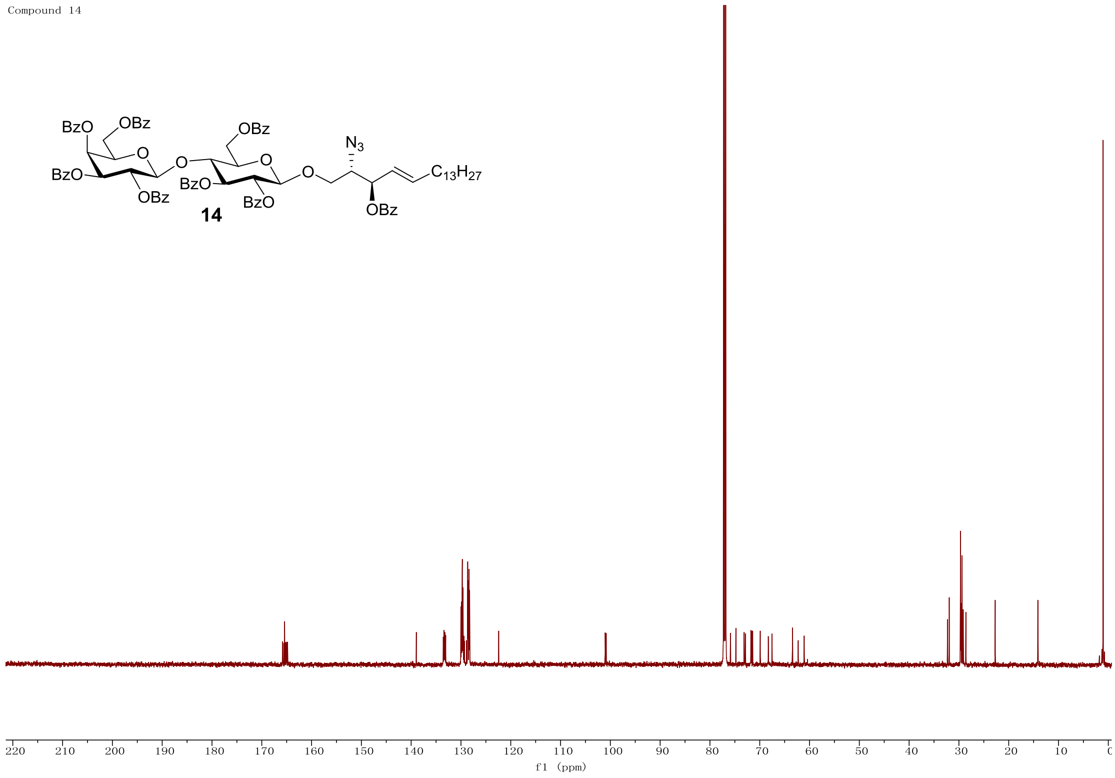

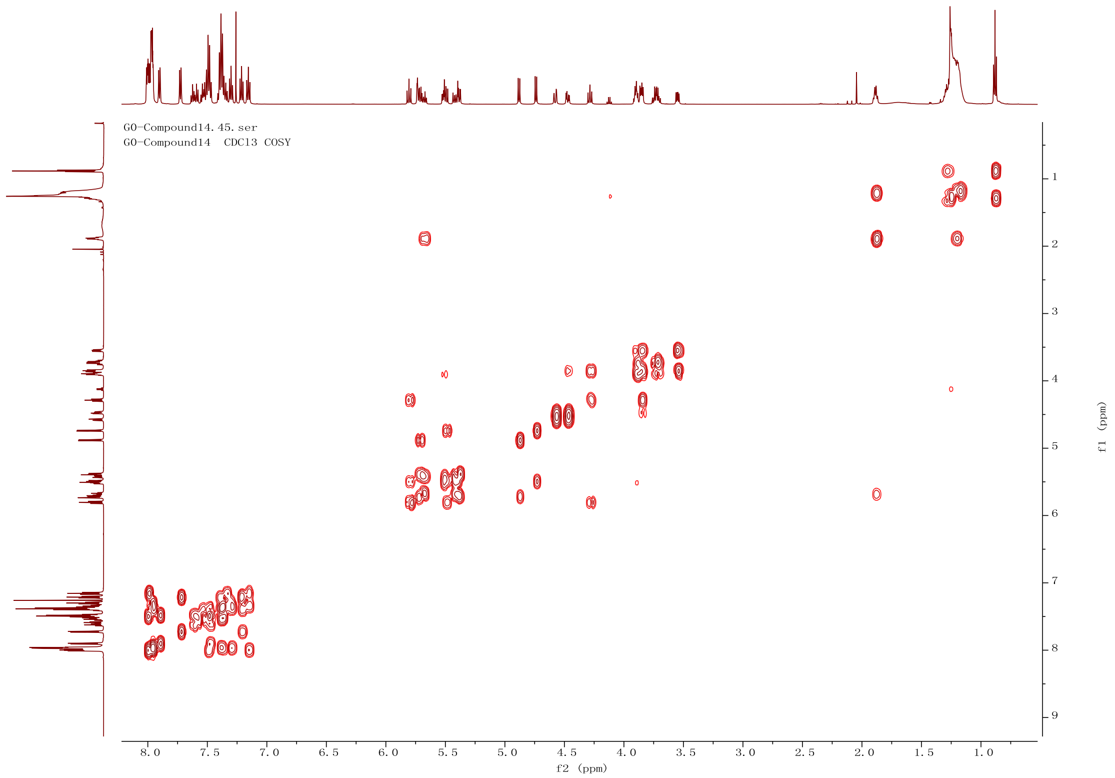
GO-Compound14.45.ser
GO-Compound14 CDCl3 COSY
f2 (ppm)
f1 (ppm)

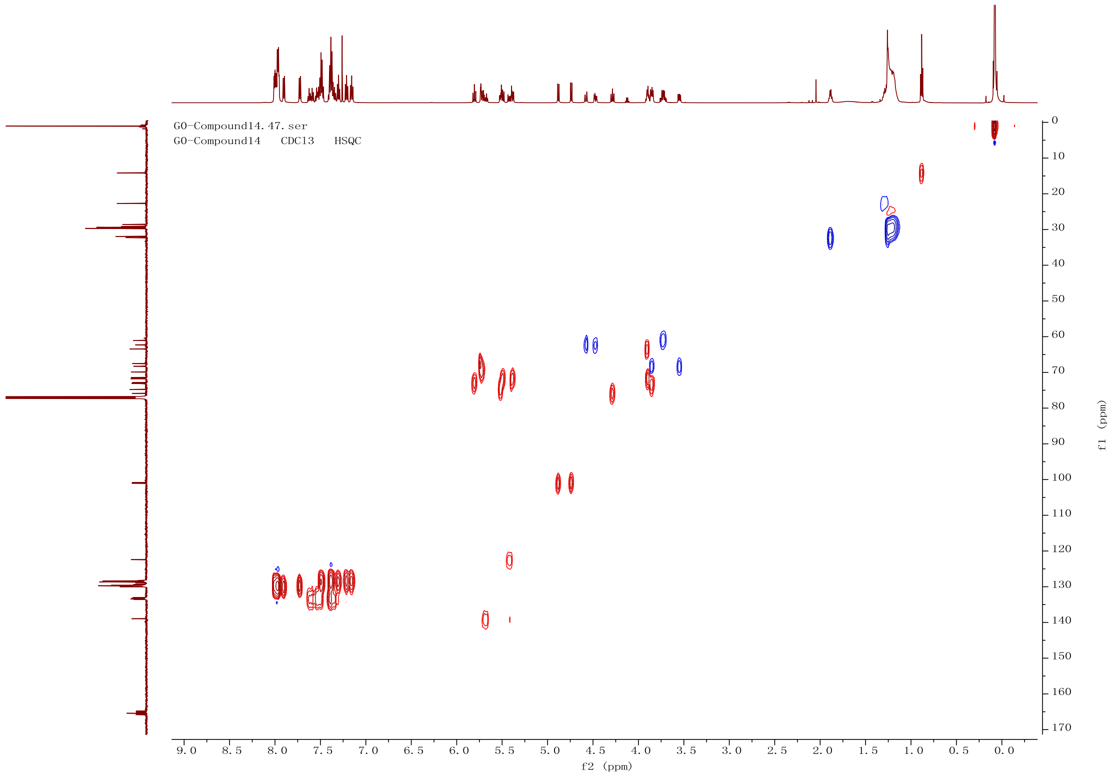
GO-Compound14.47.ser
GO-Compound14 CDCl3 HSQC
f2 (ppm)
f1 (ppm)

^{1}H NMR (500 MHz, $CDCl_3$:CD_3OD 1:1)

GO-Compound15.1.fid
GO-Compound15 CD3OD+CDCl3 1H

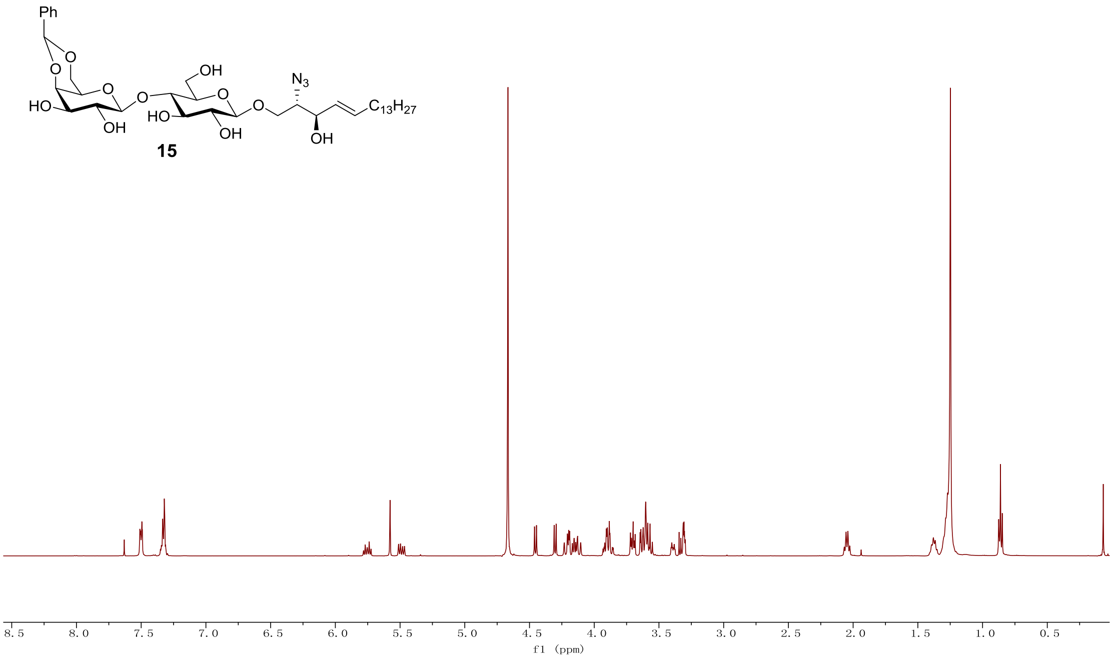

^{13}C NMR (125 MHz, $CDCl_3$:CD_3OD 1:1)

GO-Compound15.3.fid
GO-Compound15 CDCl3+CD3OD 13C-BB

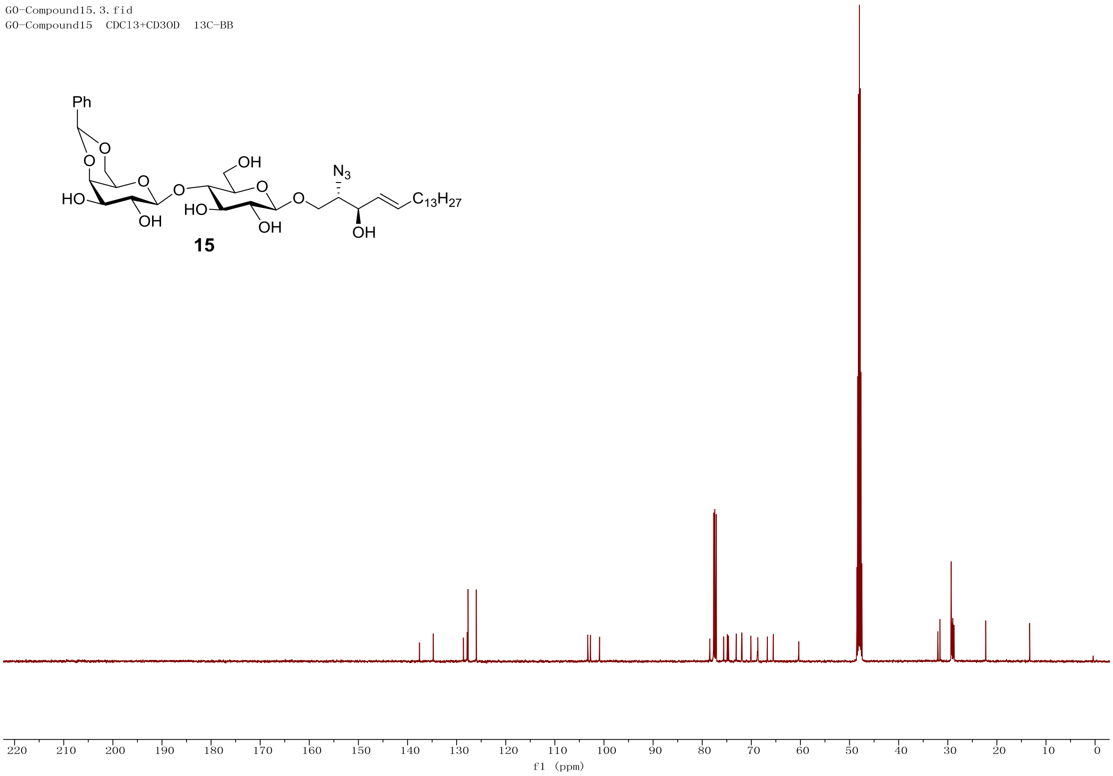

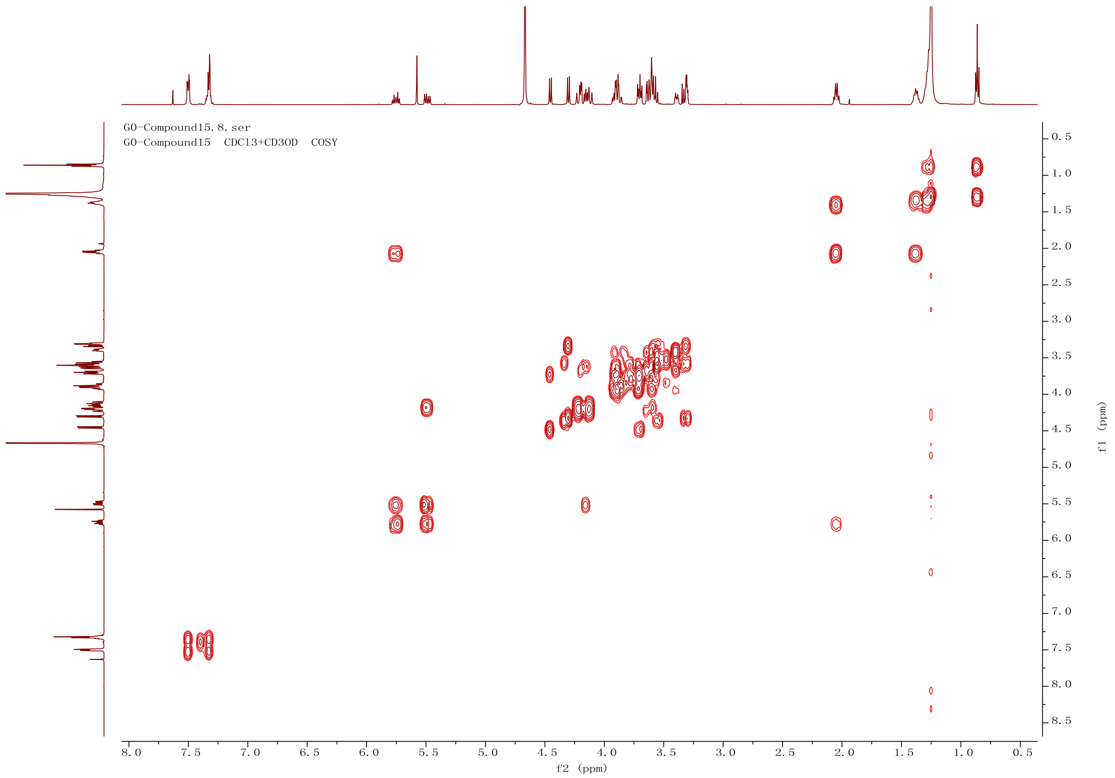
GO-Compound15.8.ser
GO-Compound15 CDCl3+CD3OD COSY
f2 (ppm)
f1 (ppm)

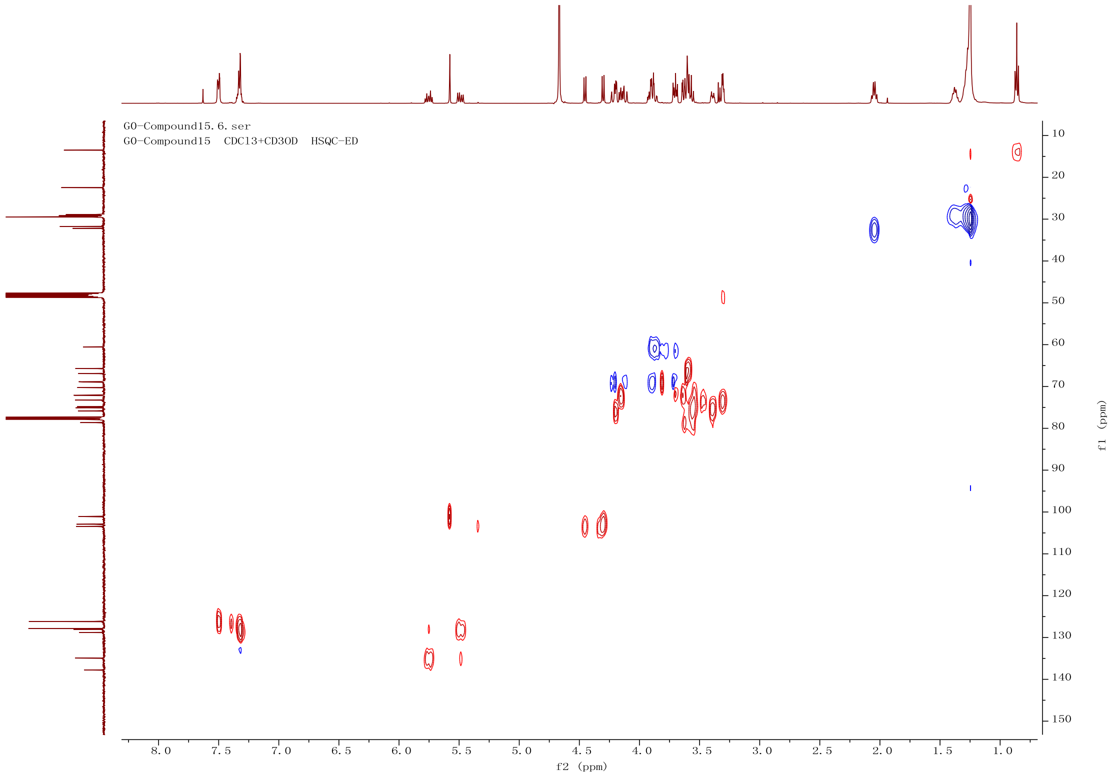
GO-Compound15.6.ser
GO-Compound15 CDCl3+CD3OD HSQC-ED
f2 (ppm)
f1 (ppm)

^{1}H NMR (500 MHz, $CDCl_3$)

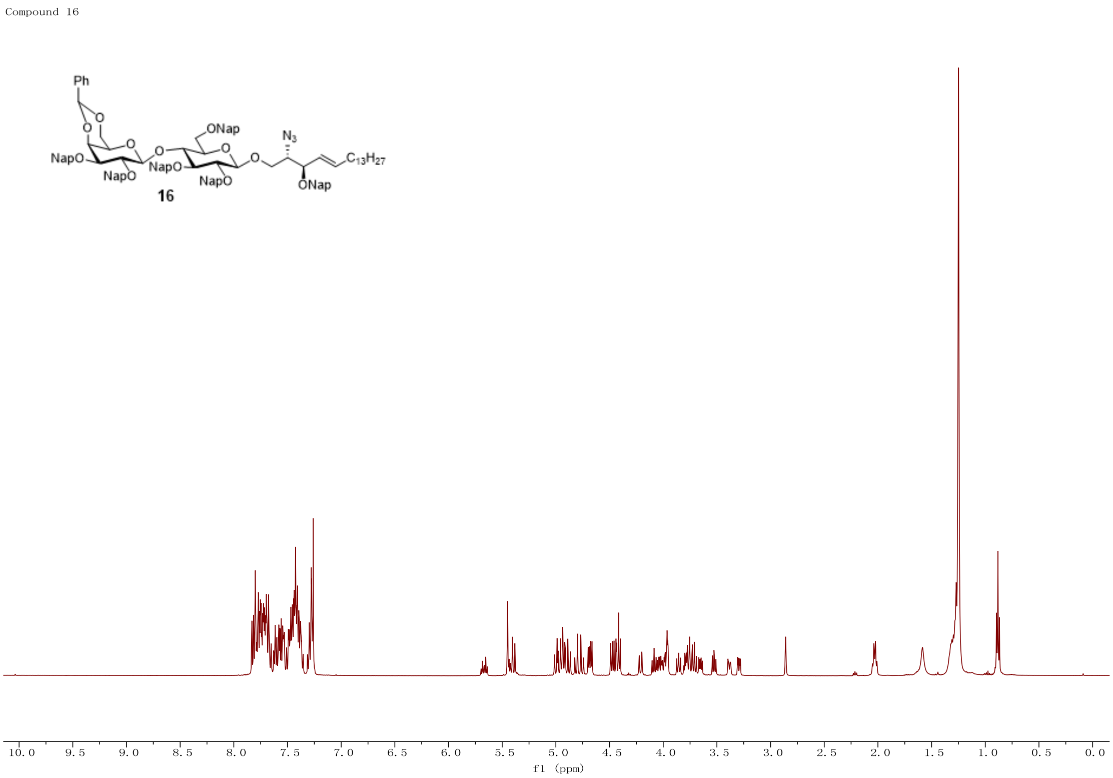

^{13}C NMR (125 MHz, $CDCl_3$)

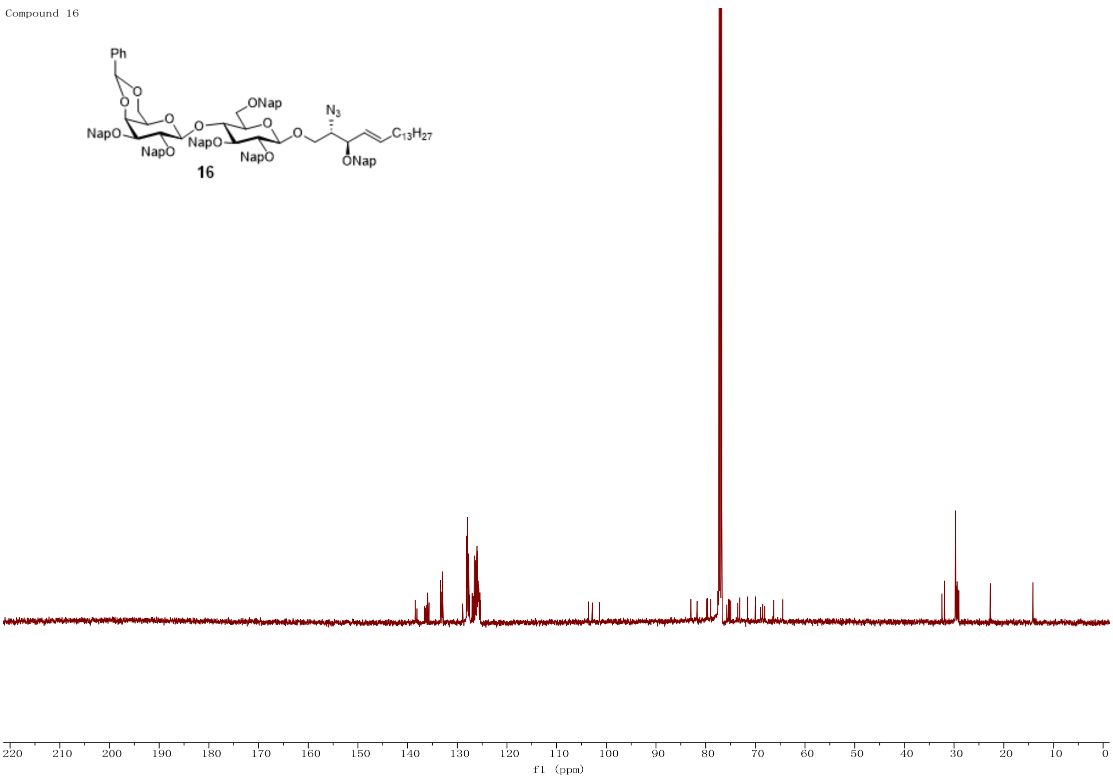

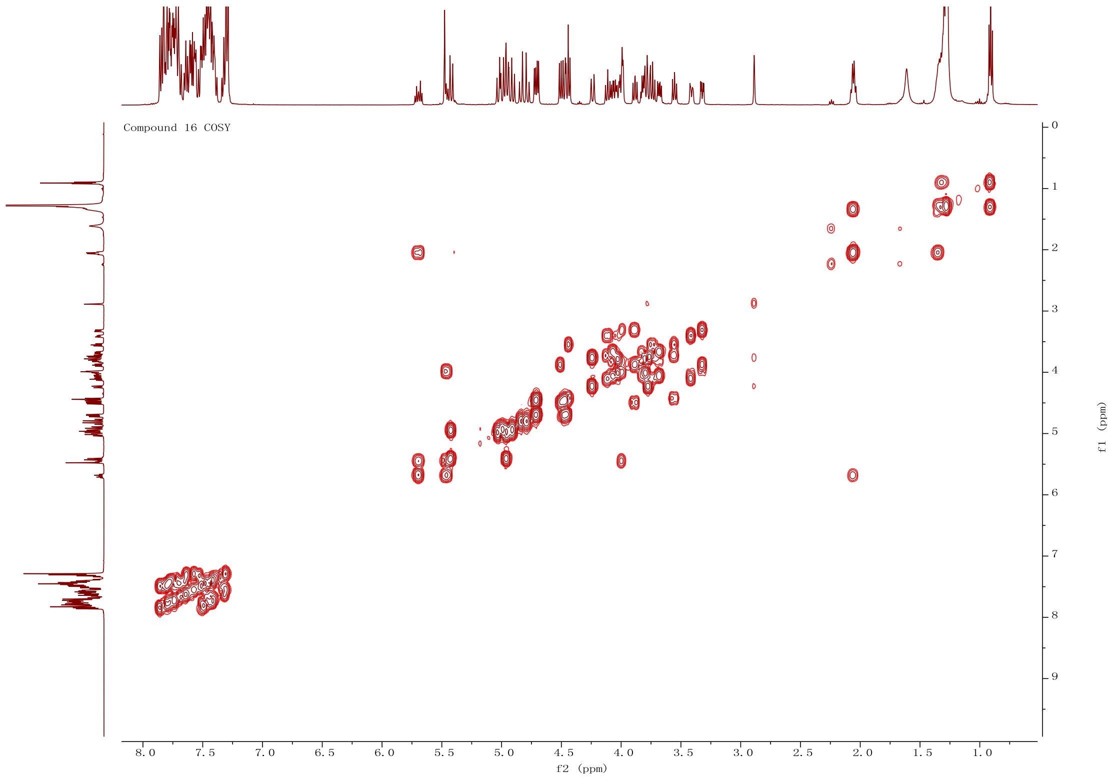
Compound 16 COSY
f2 (ppm)
f1 (ppm)

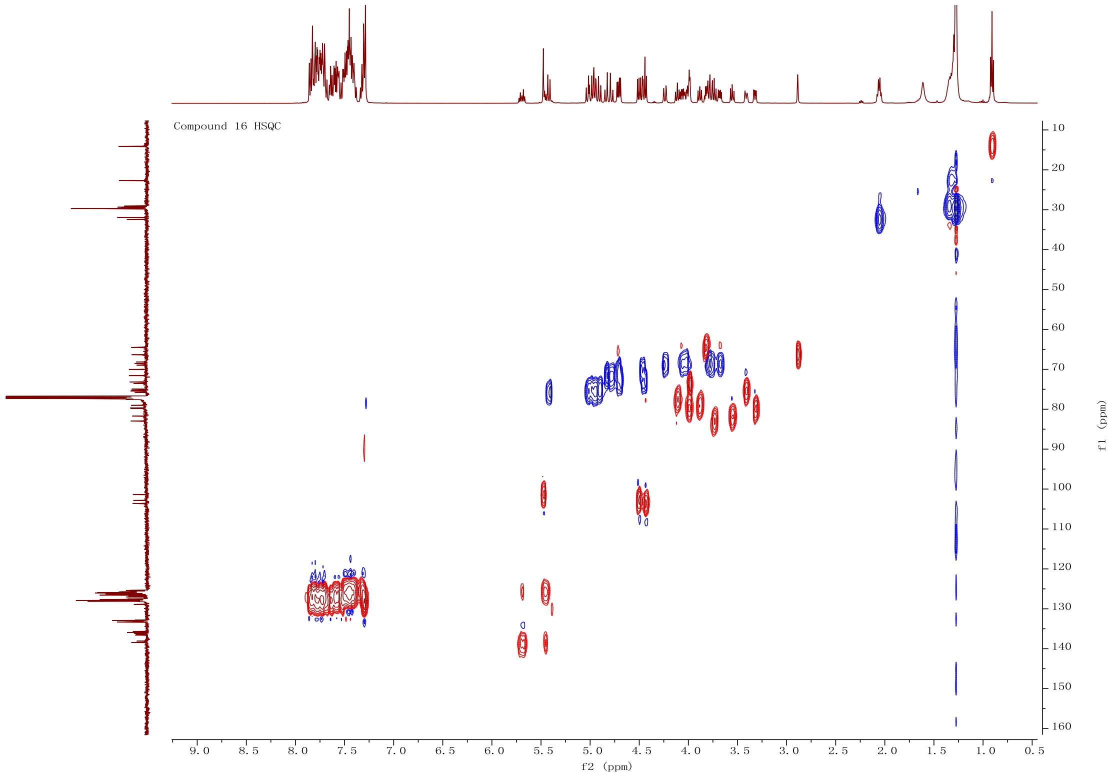
Compound 16 HSQC
f2 (ppm)
f1 (ppm)

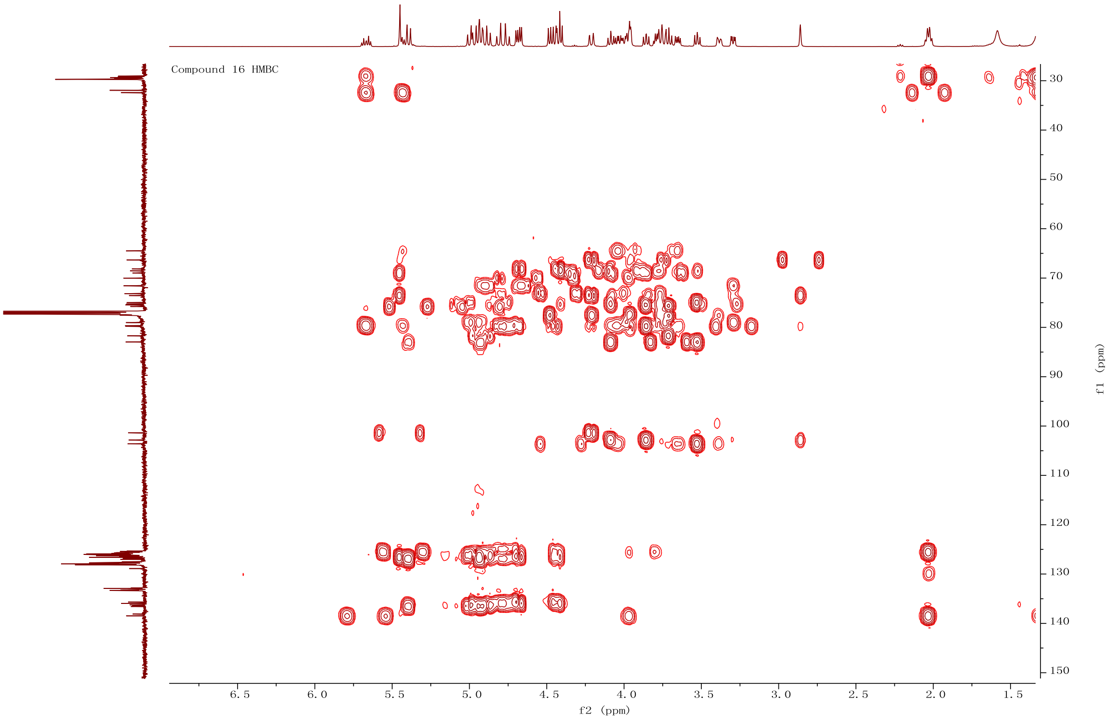

^{1}H NMR (600 MHz, $CDCl_3$)

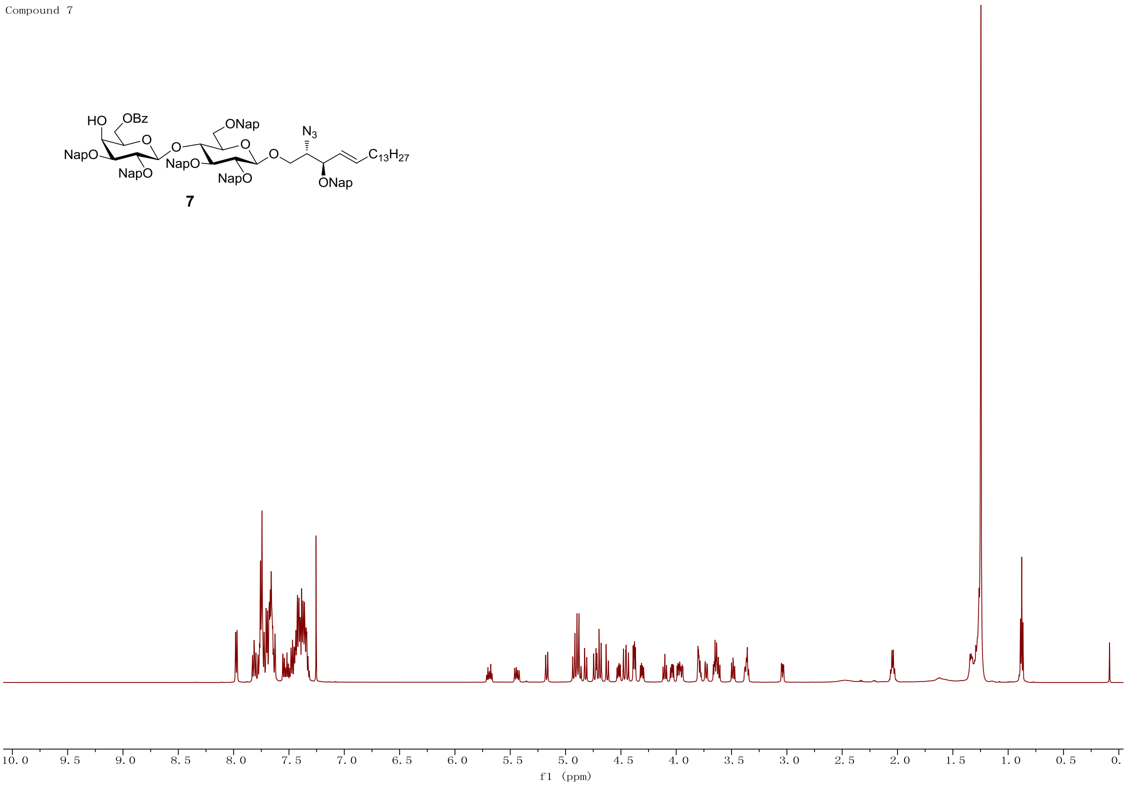

^{13}C NMR (150 MHz, CDCl$_3$)

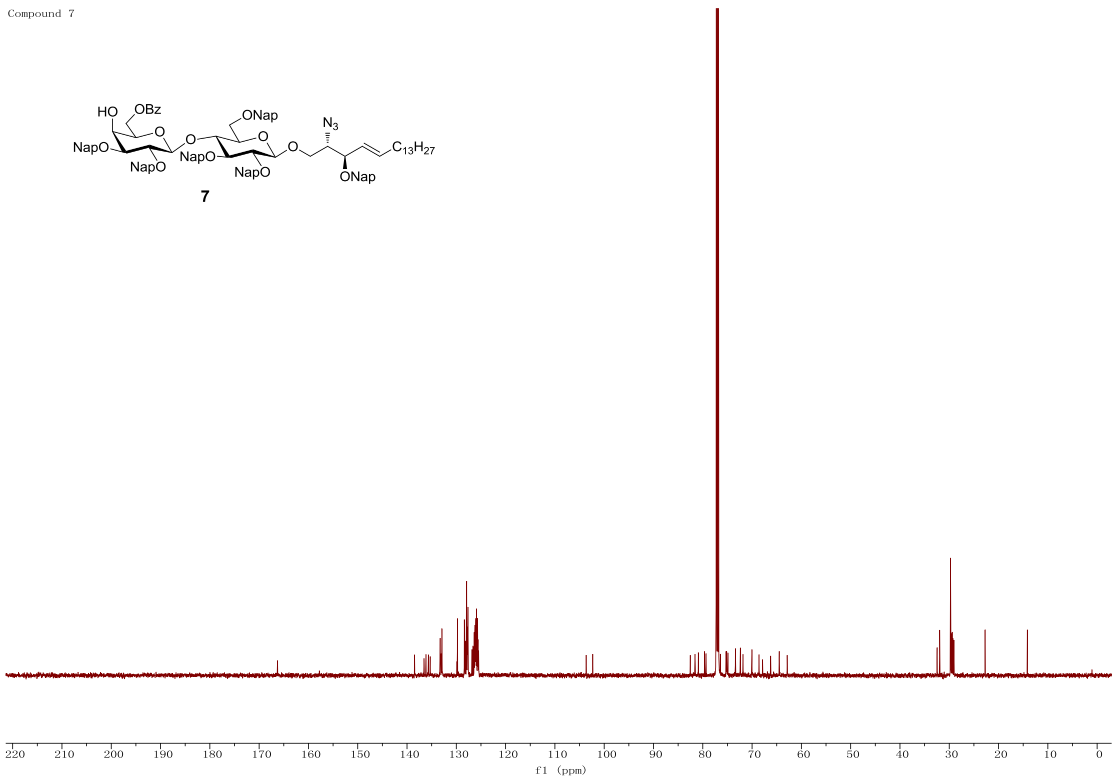

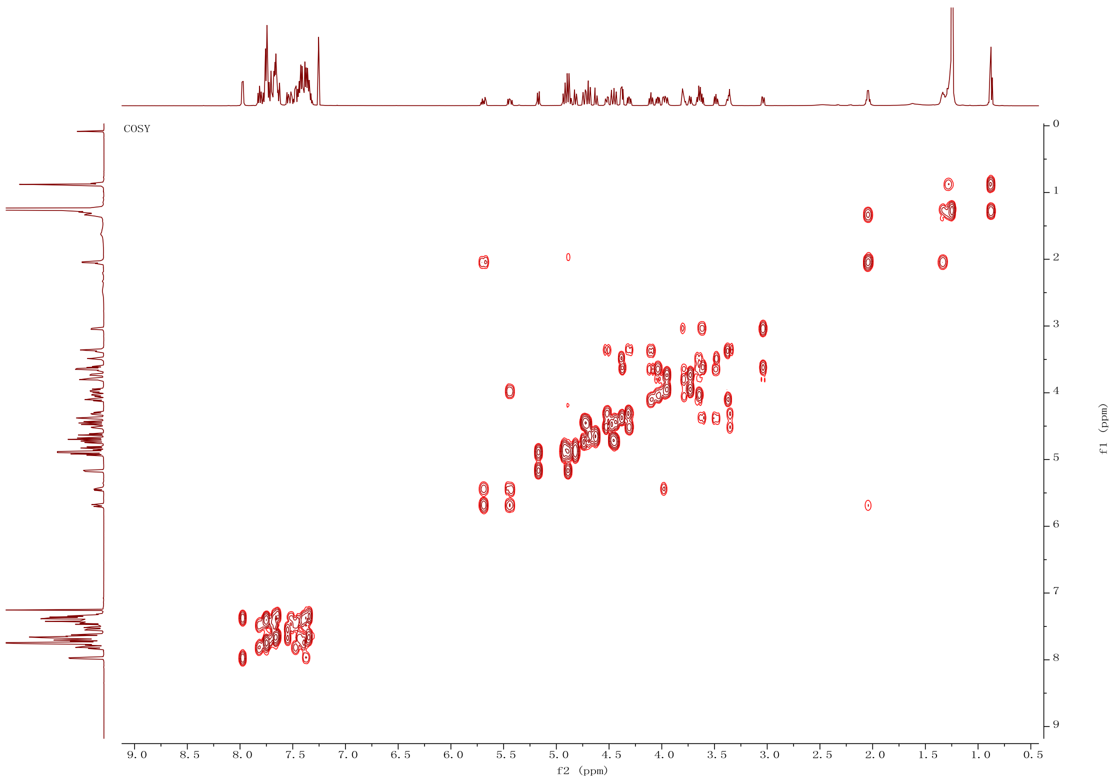

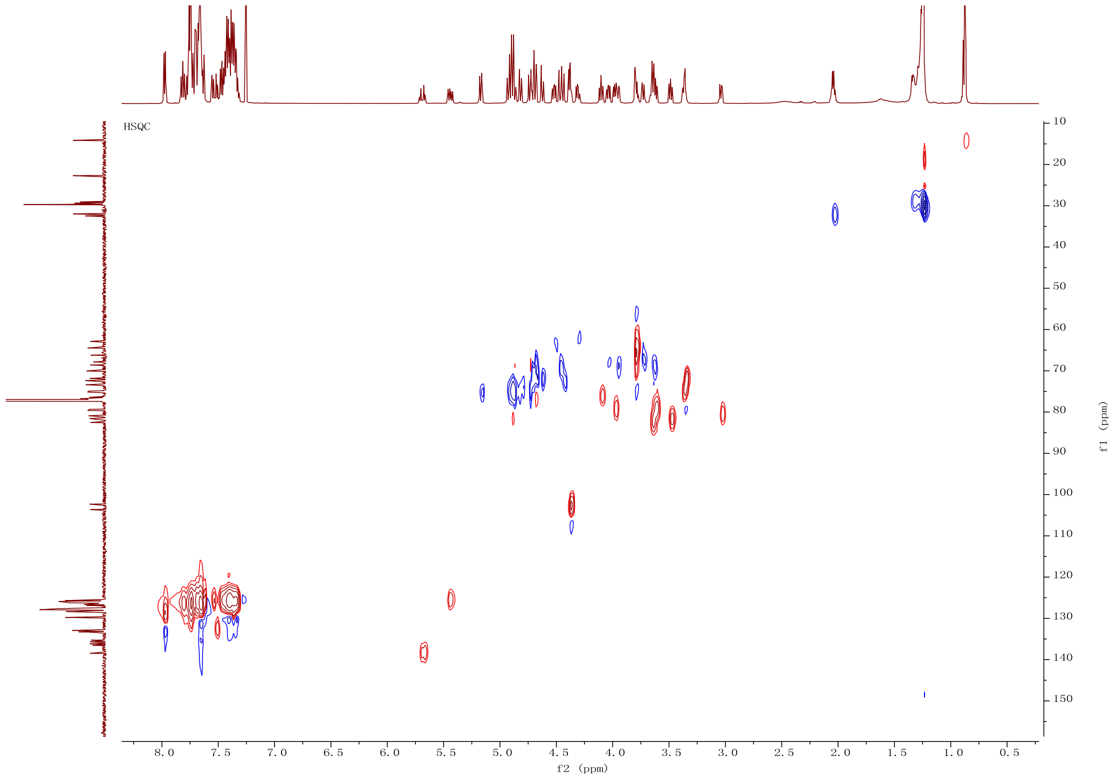

^{1}H NMR (500 MHz, CDCl$_3$)

Compound 17

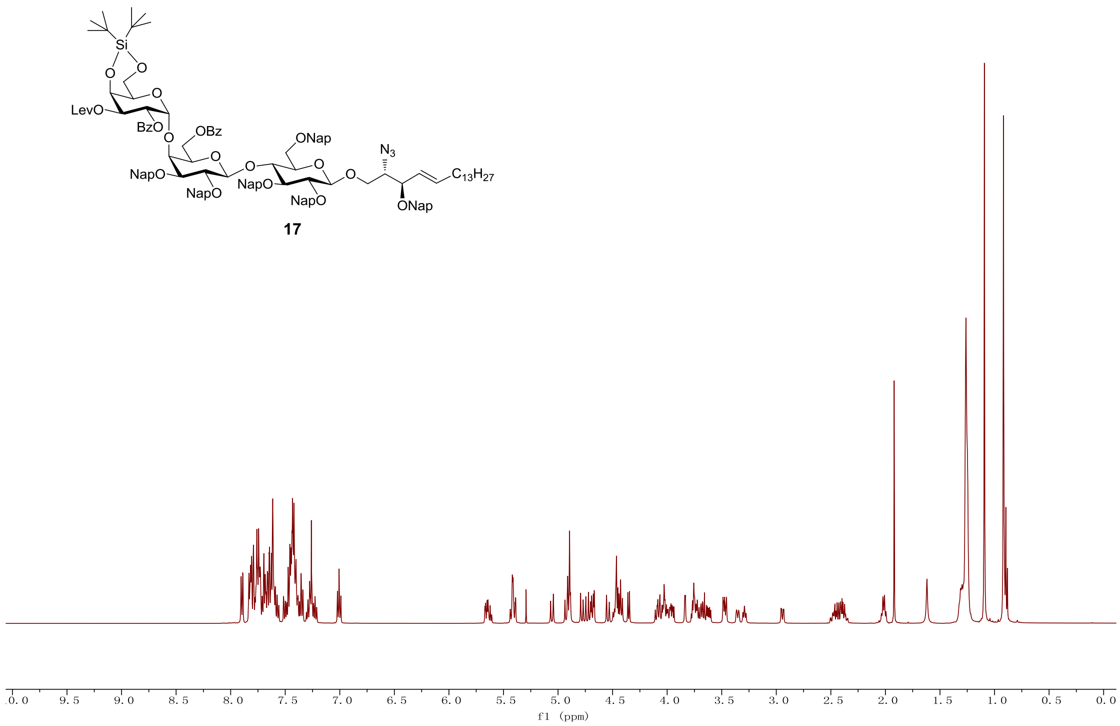

^{13}C NMR (125 MHz, $CDCl_3$)

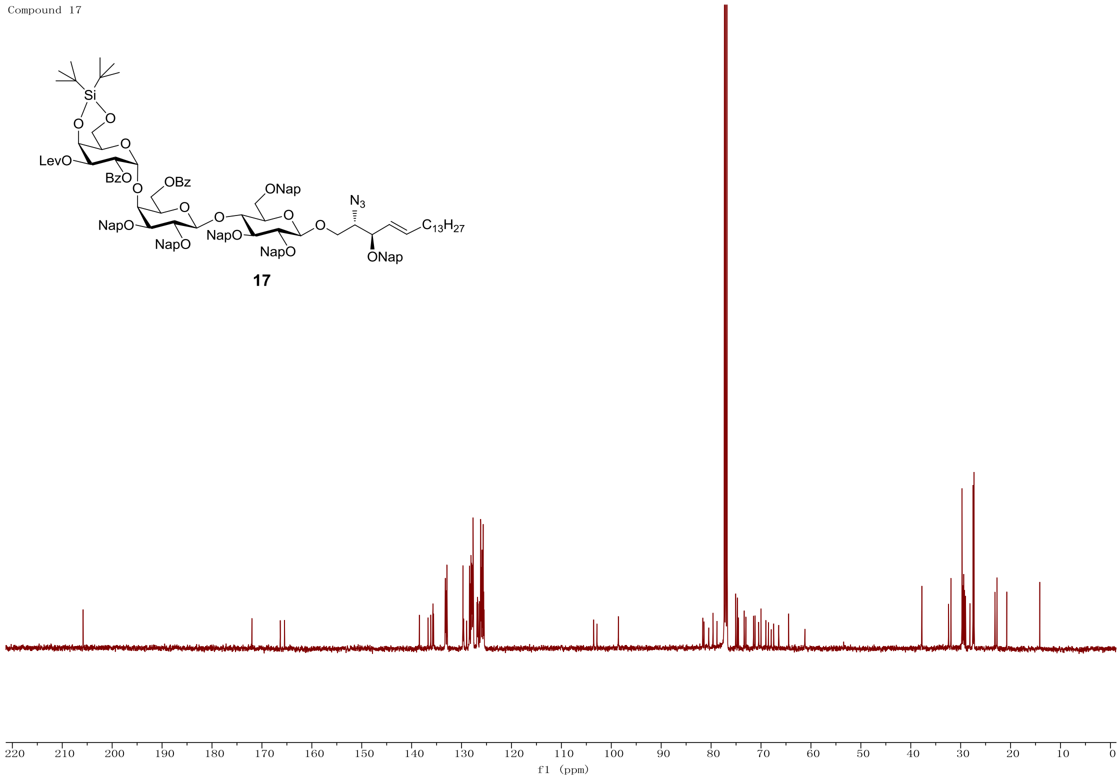

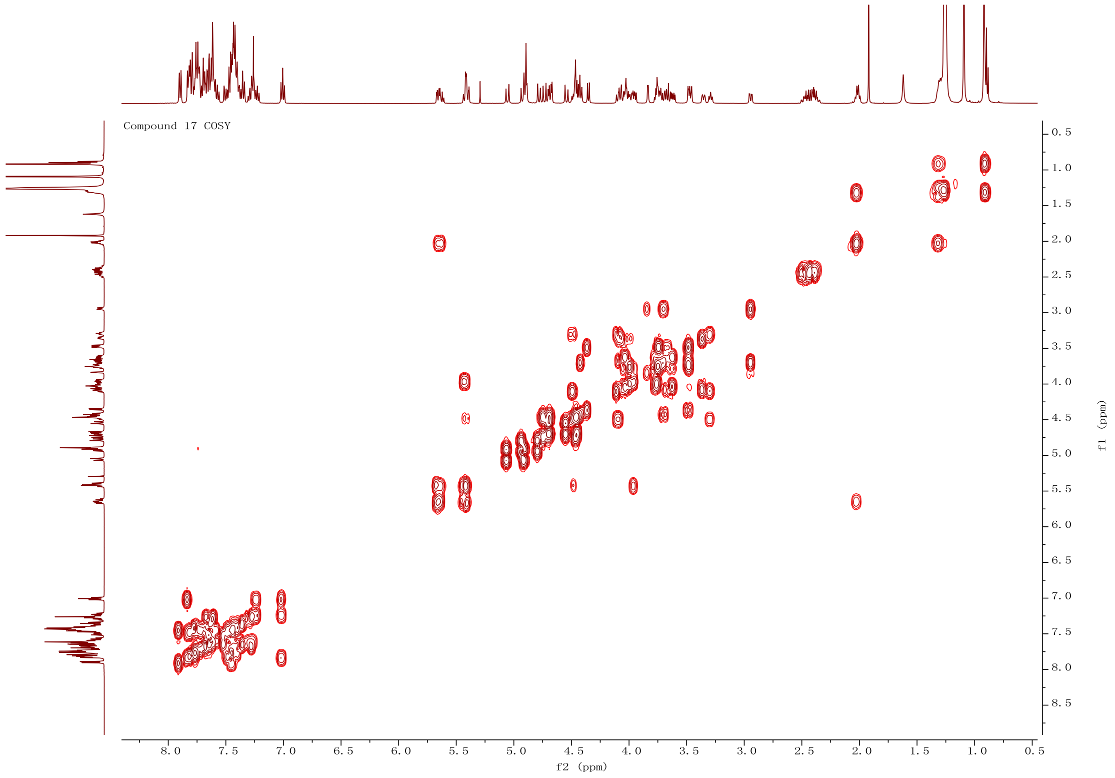

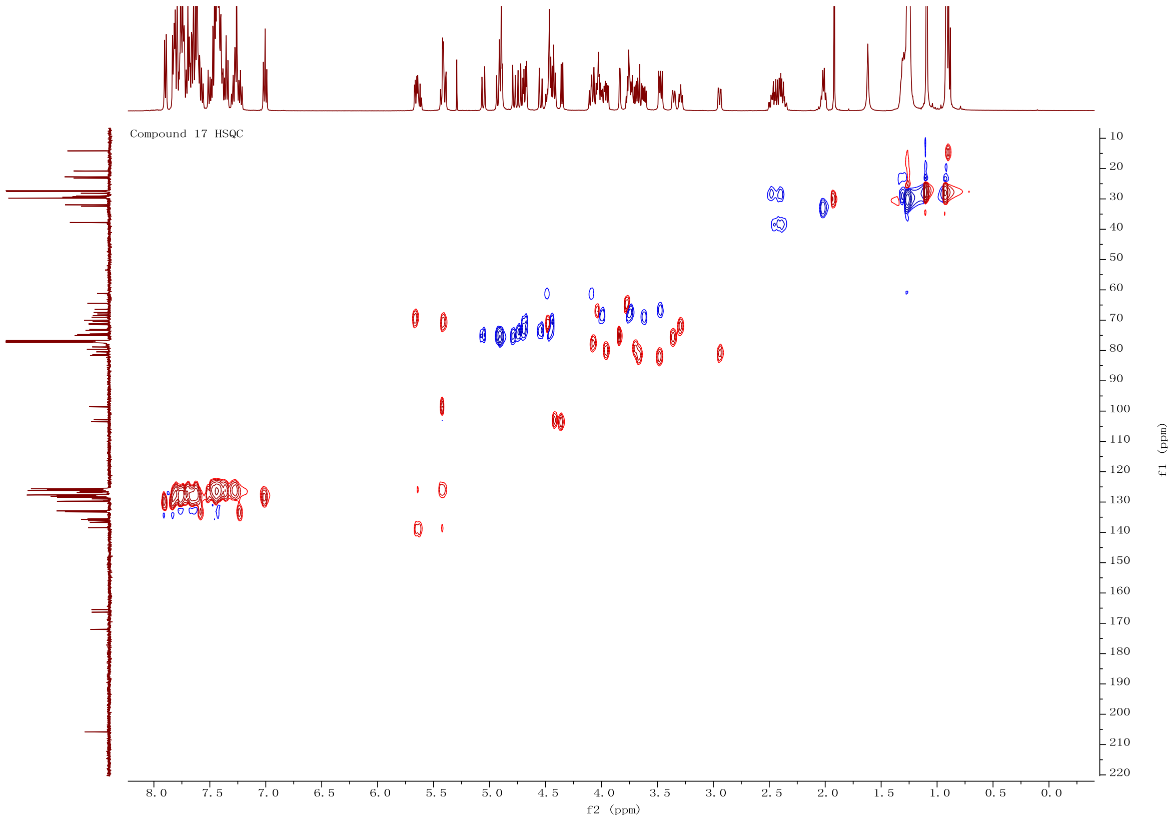
Compound 17 HSQC
f2 (ppm)
f1 (ppm)

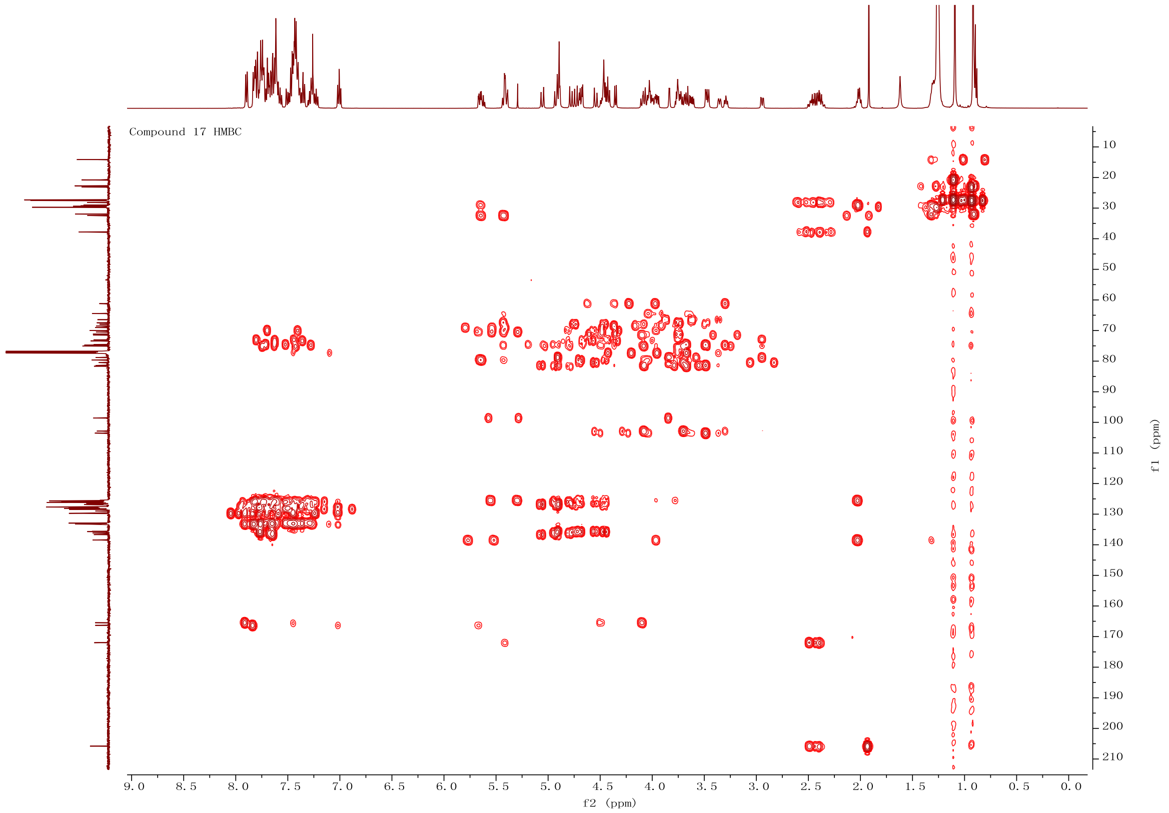
Compound 17 HMBC
f2 (ppm)
f1 (ppm)

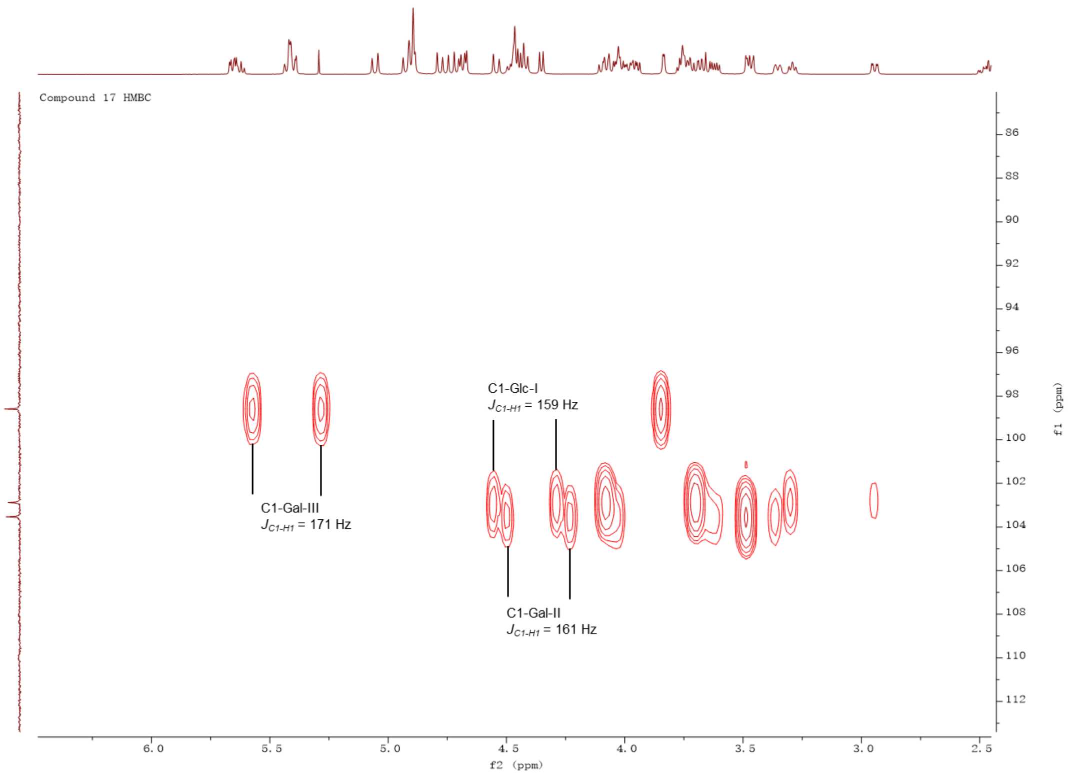

^{1}H NMR (400 MHz, CDCl$_3$)

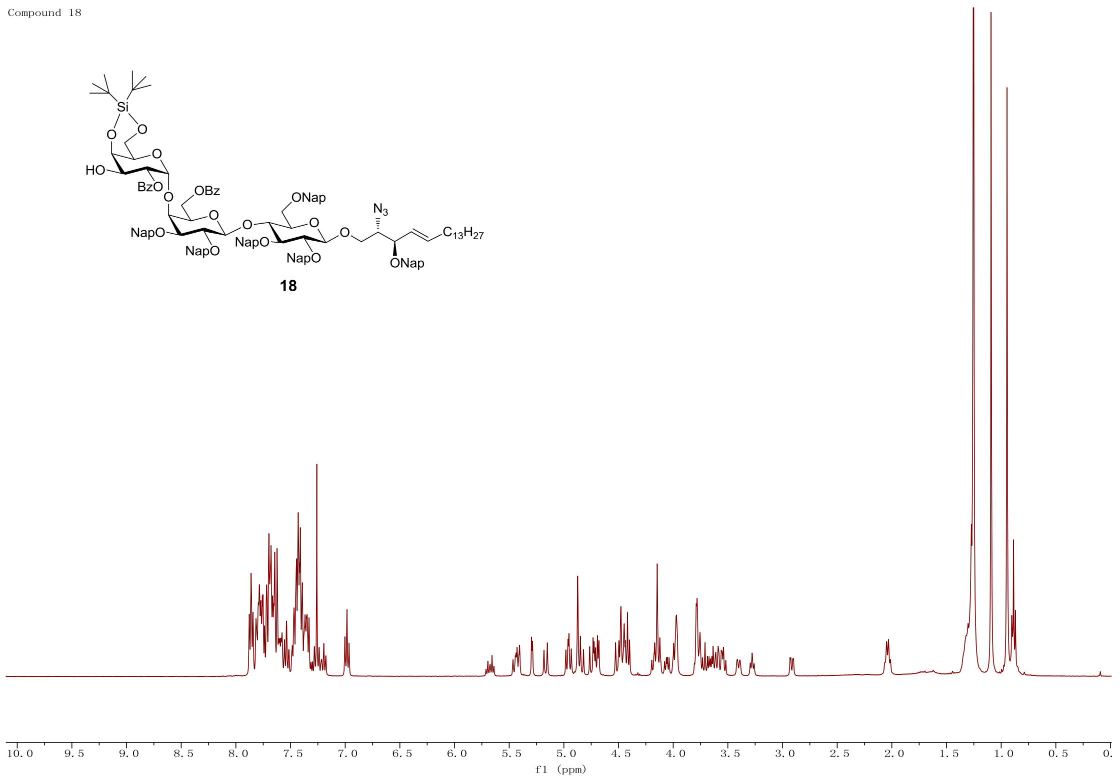

^{13}C NMR (100 MHz, CDCl$_3$)

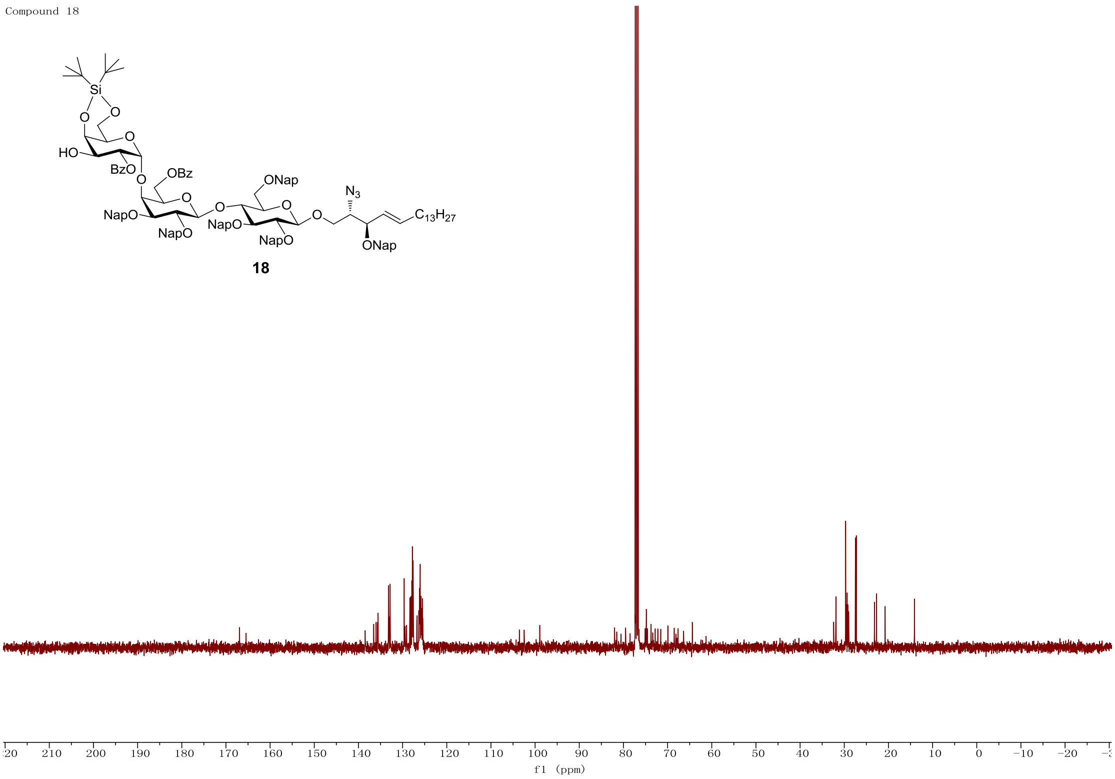

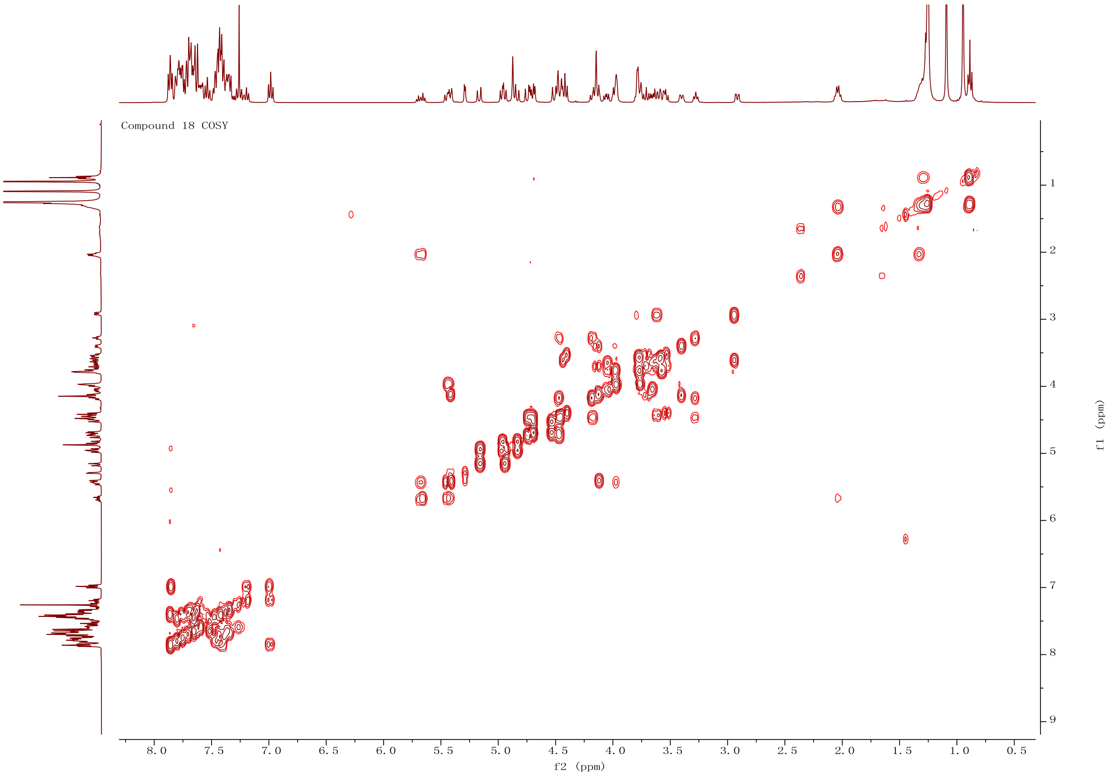

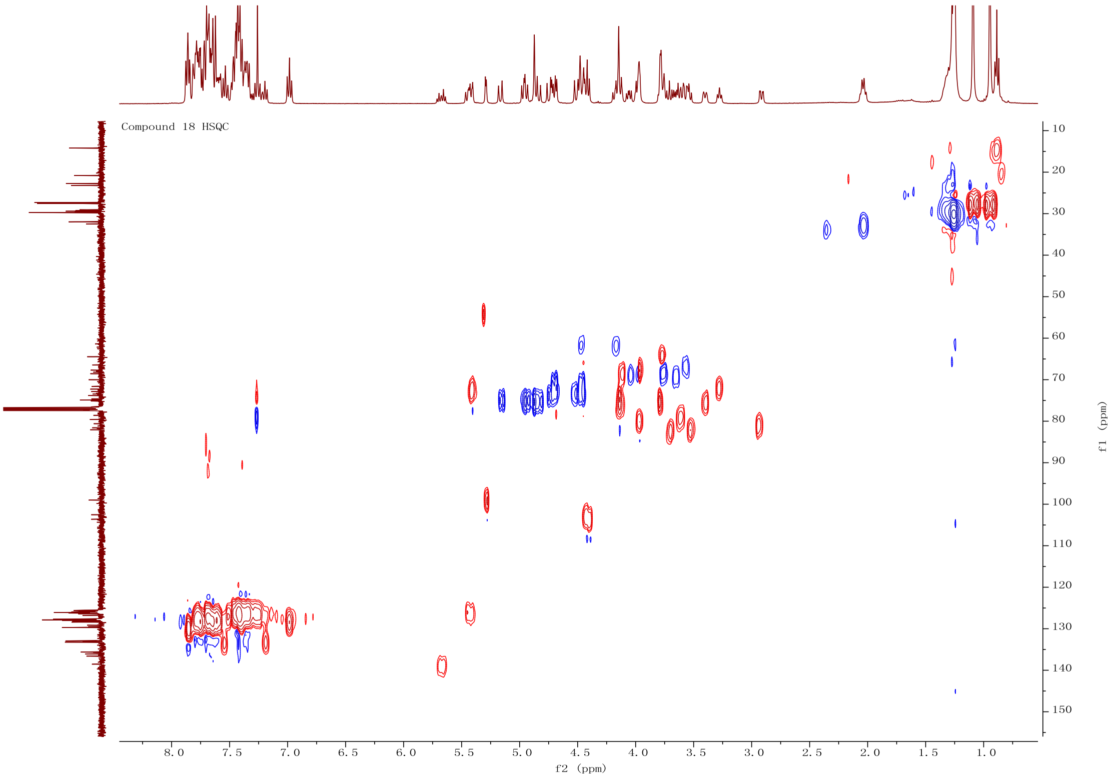
Compound 18 HSQC
f2 (ppm)
f1 (ppm)

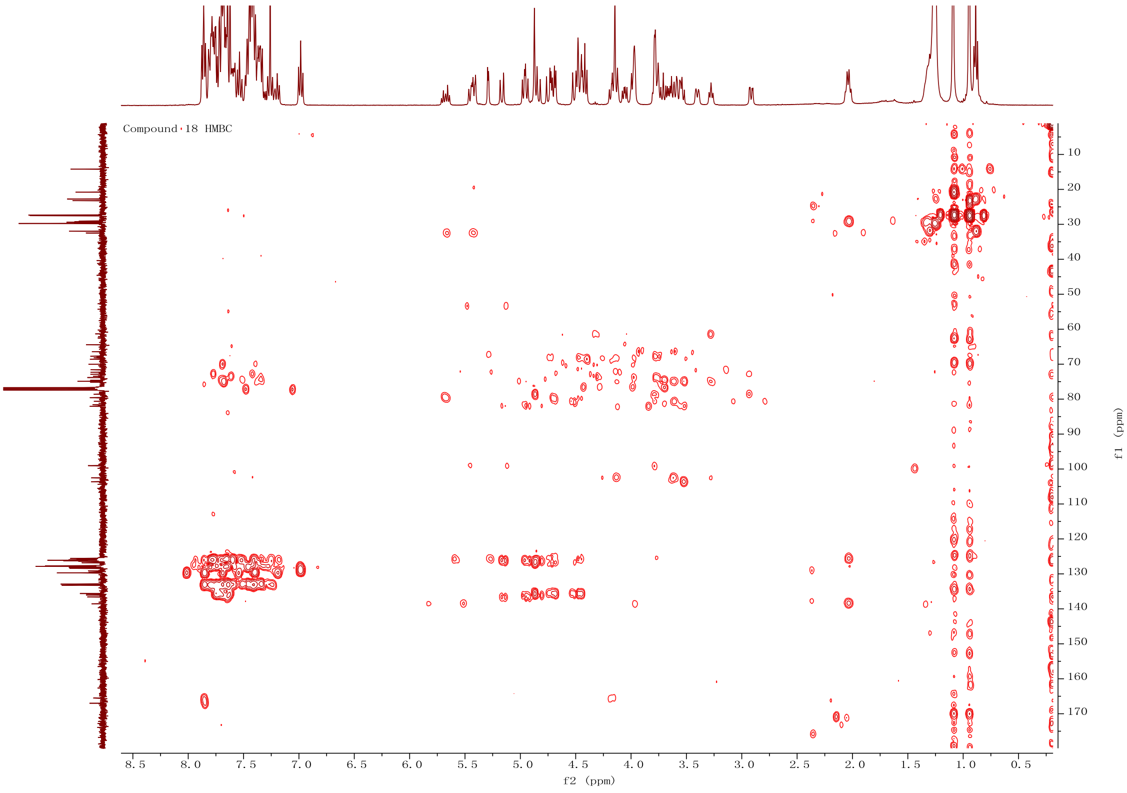
Compound 18 HMBC
f2 (ppm)
f1 (ppm)

^{1}H NMR (600 MHz, $CDCl_3$)

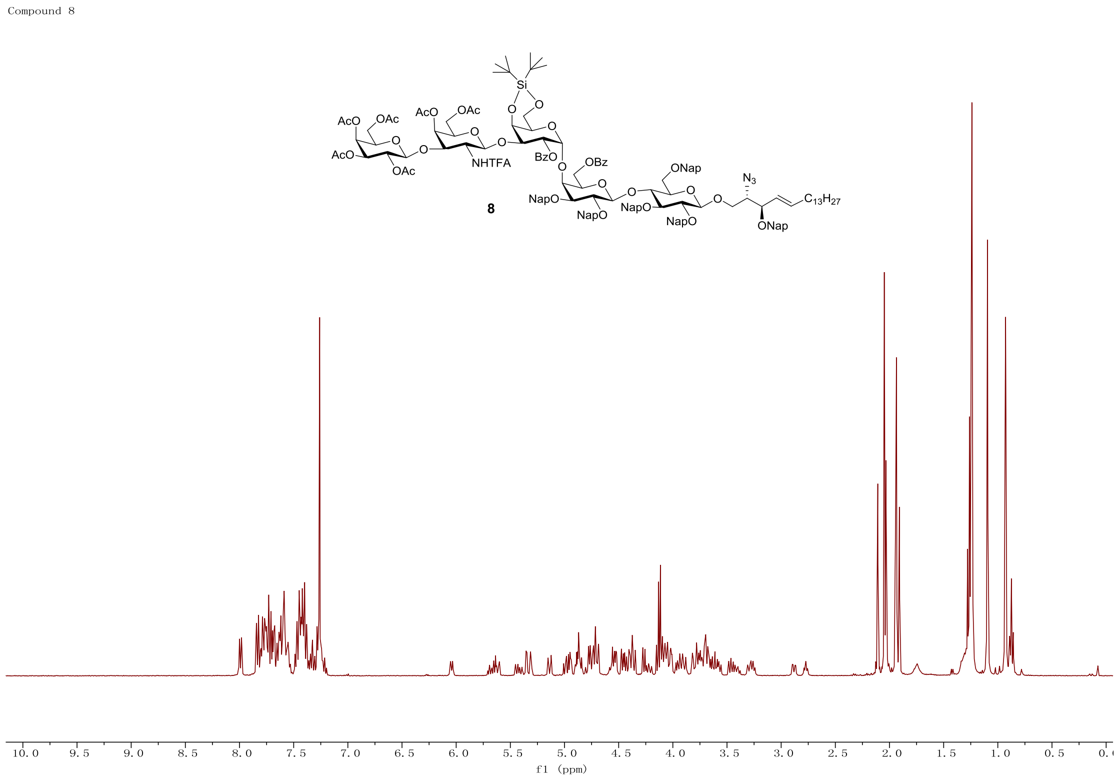

^{13}C NMR (150 MHz, $CDCl_3$)

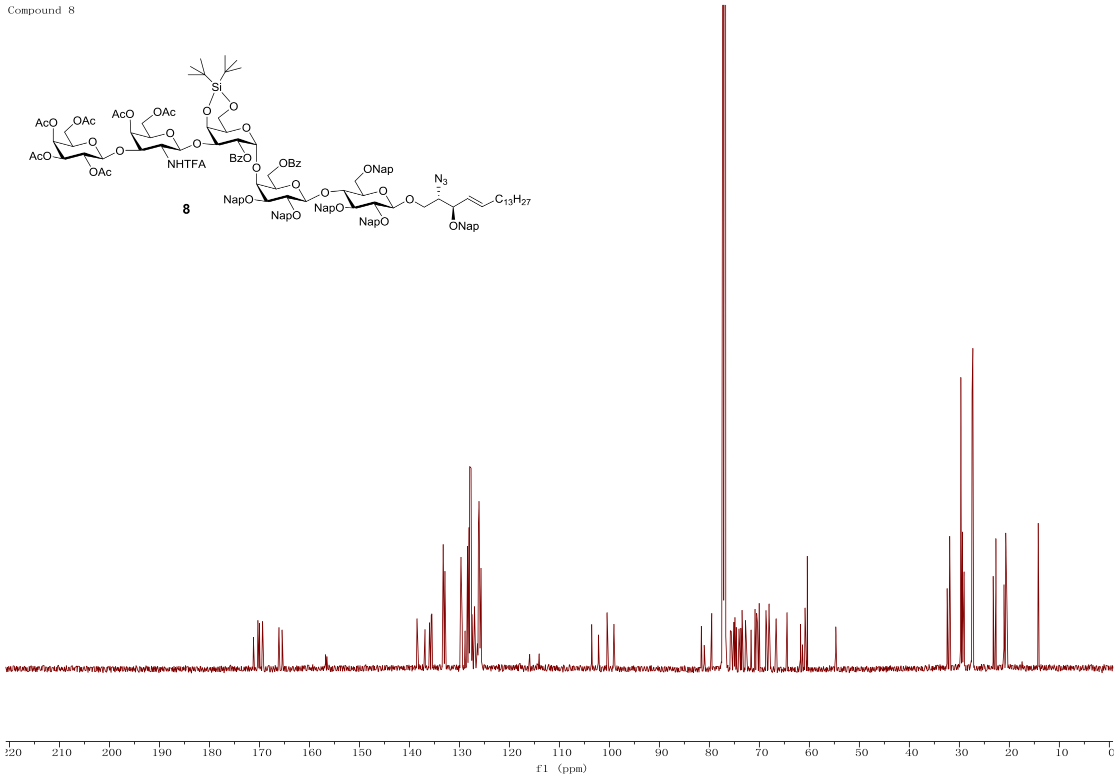

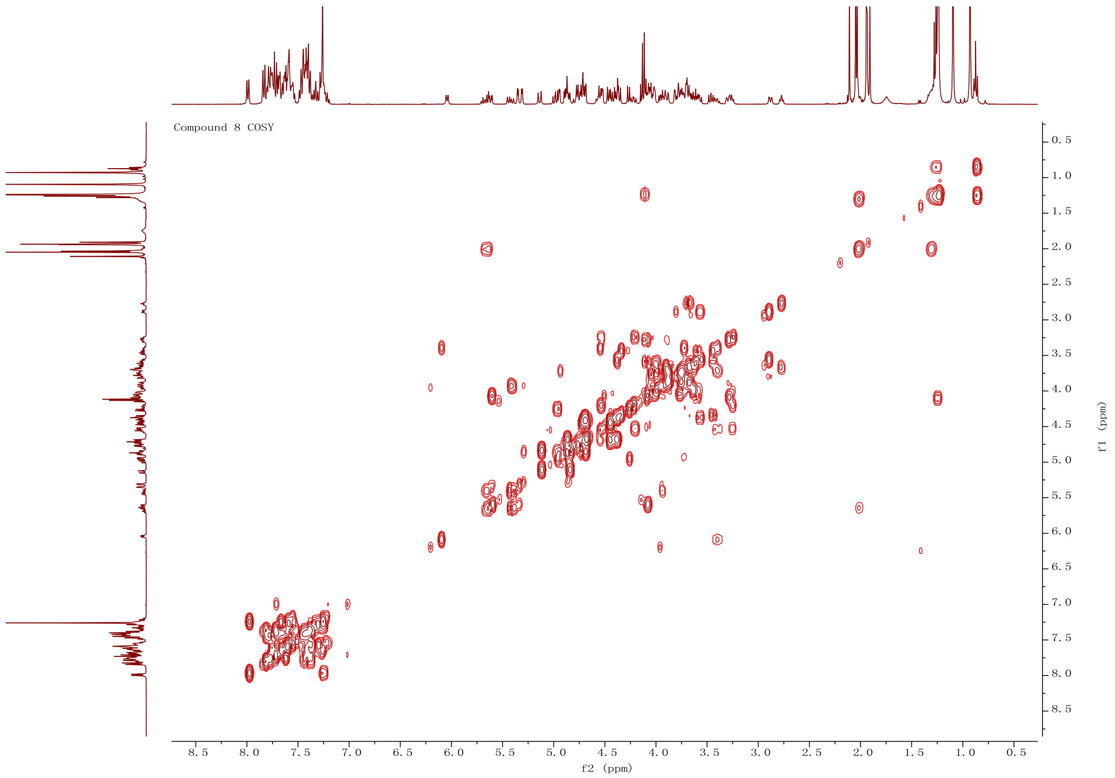
Compound 8 COSY
f2 (ppm)
f1 (ppm)

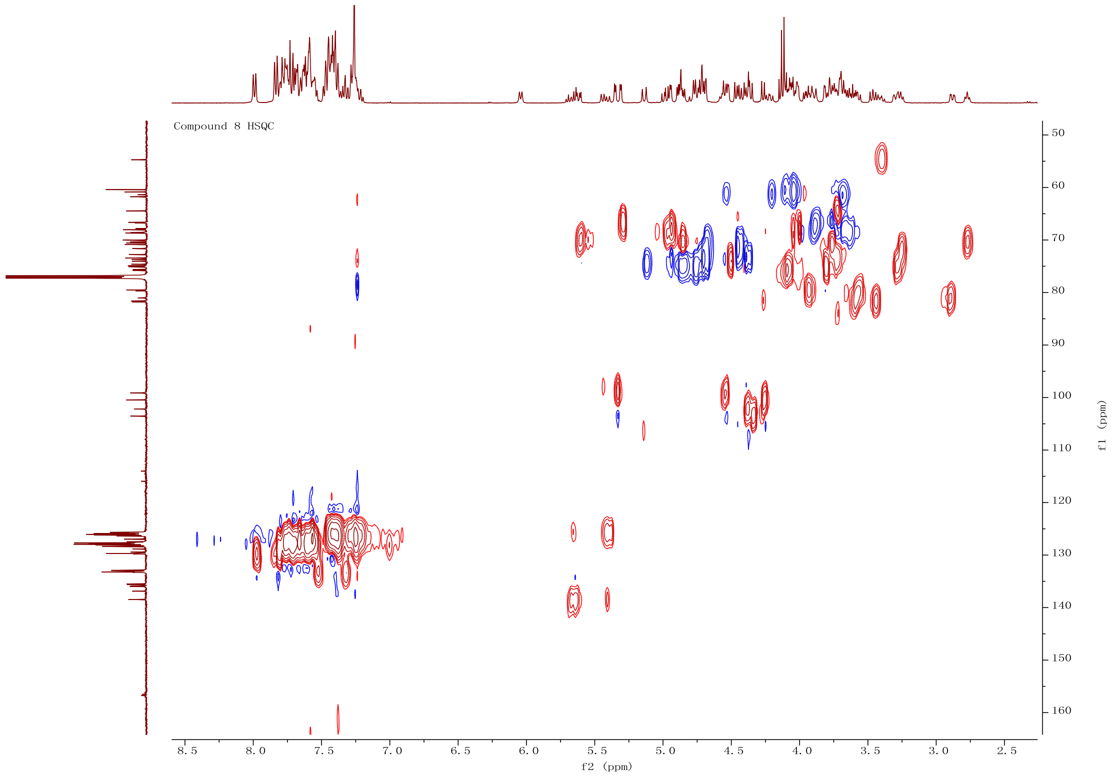
Compound 8 HSQC
f2 (ppm)
f1 (ppm)

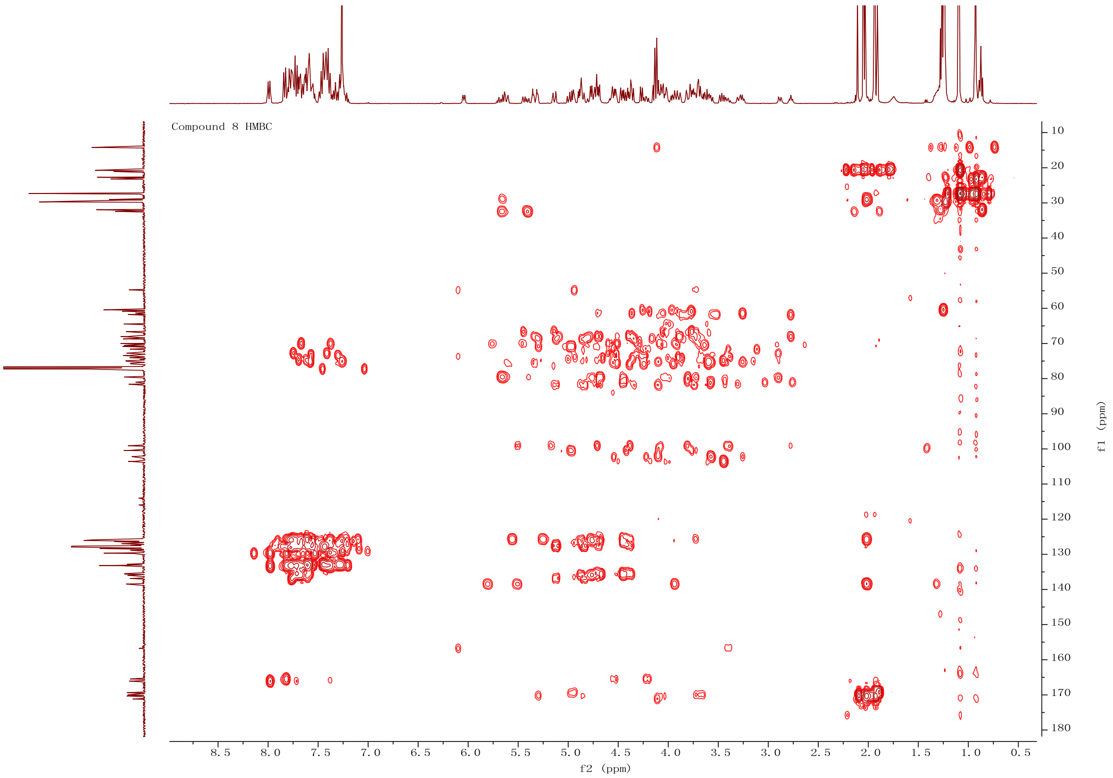

Compound 8 HMBC
f2 (ppm)
f1 (ppm)

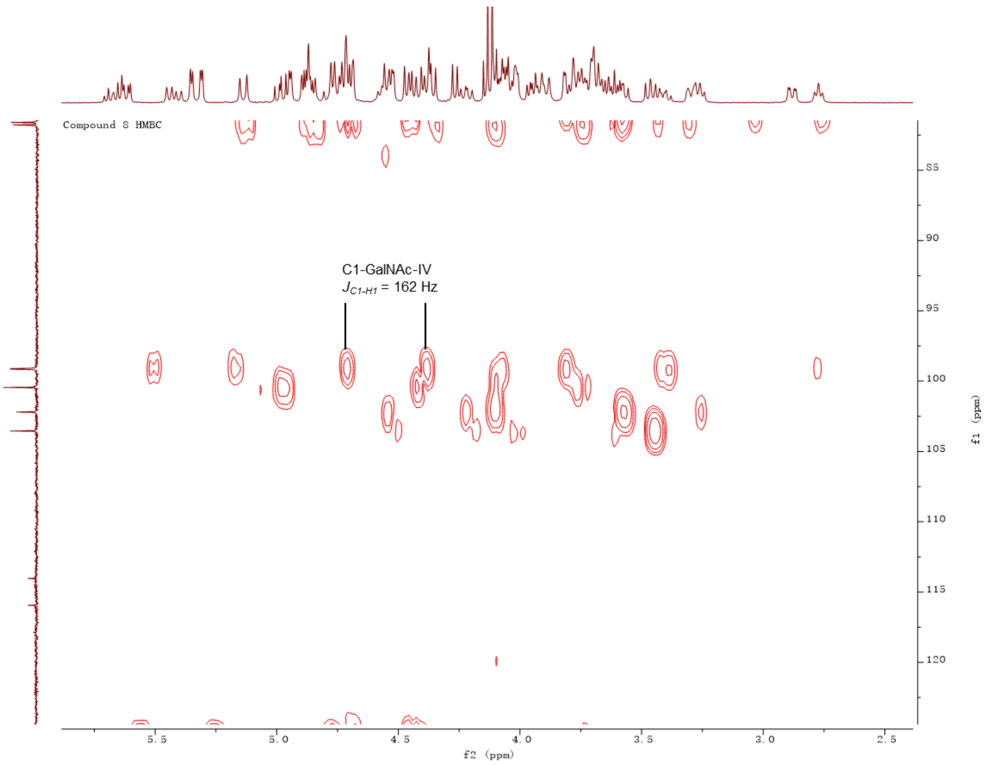

Compound 8 HMBC
C1-GalNAc-IV
$J_{C1\text{-}H1}$ = 162 Hz
f2 (ppm)
f1 (ppm)

^{1}H NMR (500 MHz, $CDCl_3$:CD_3OD 1:1)

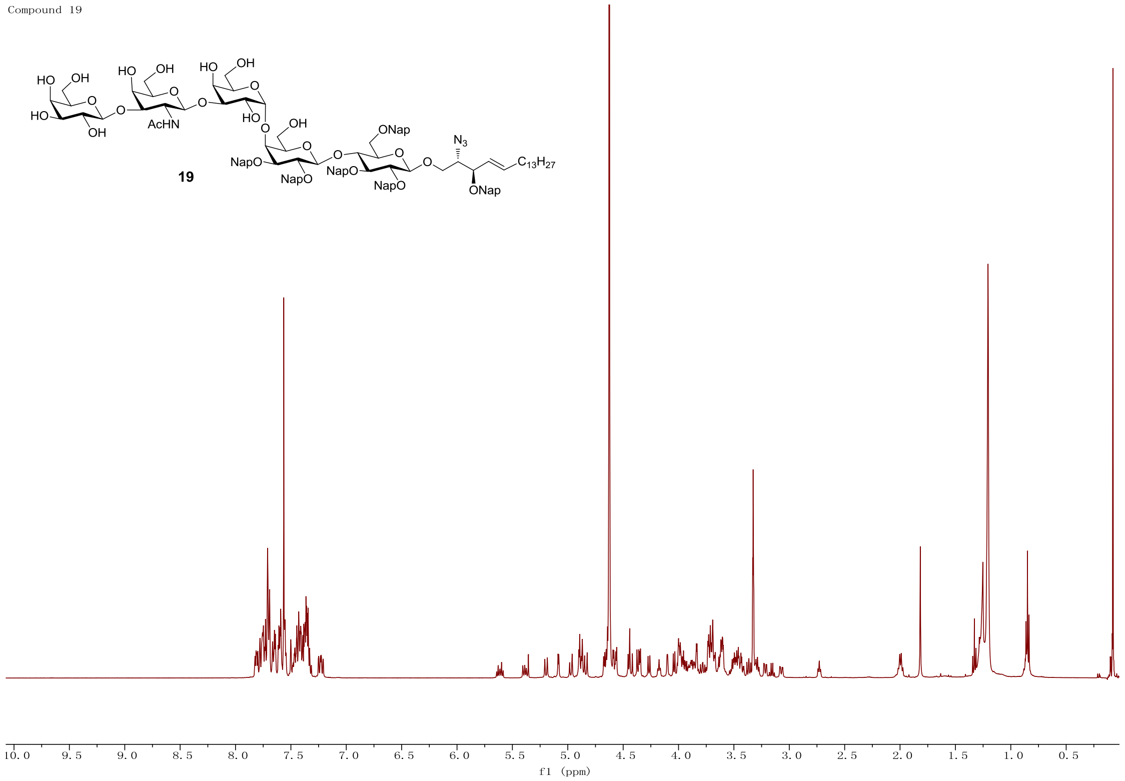

^{13}C NMR (125 MHz, $CDCl_3$:CD_3OD=1:1)

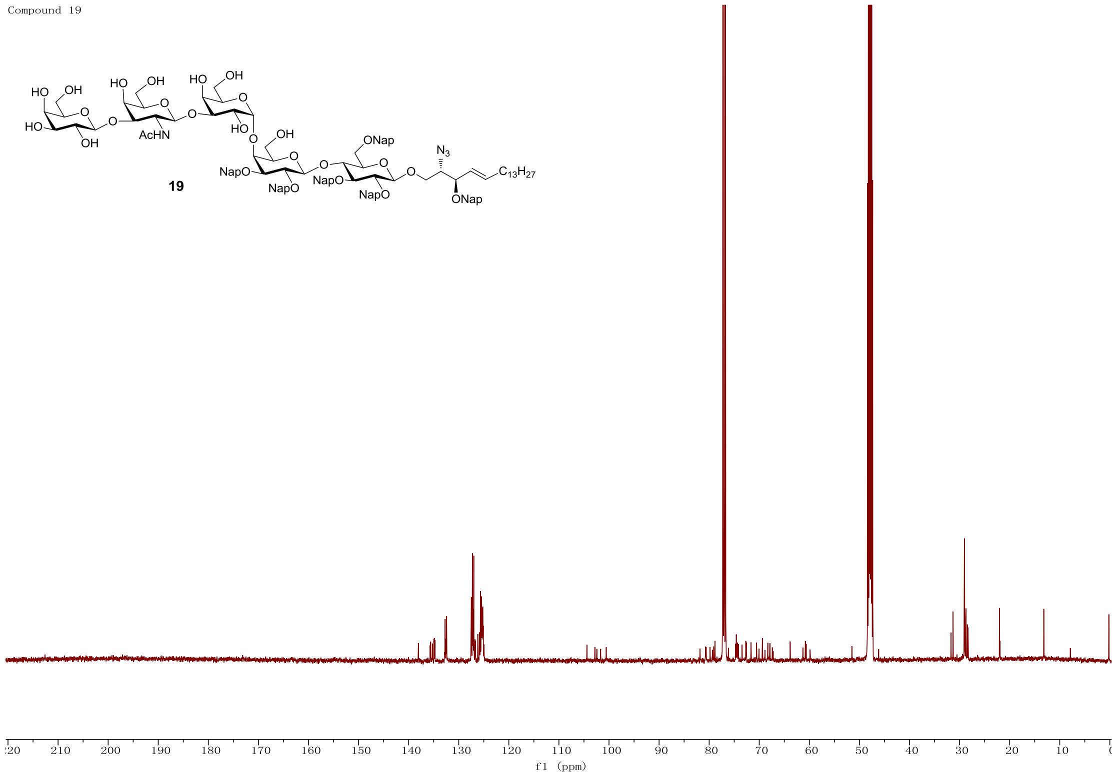

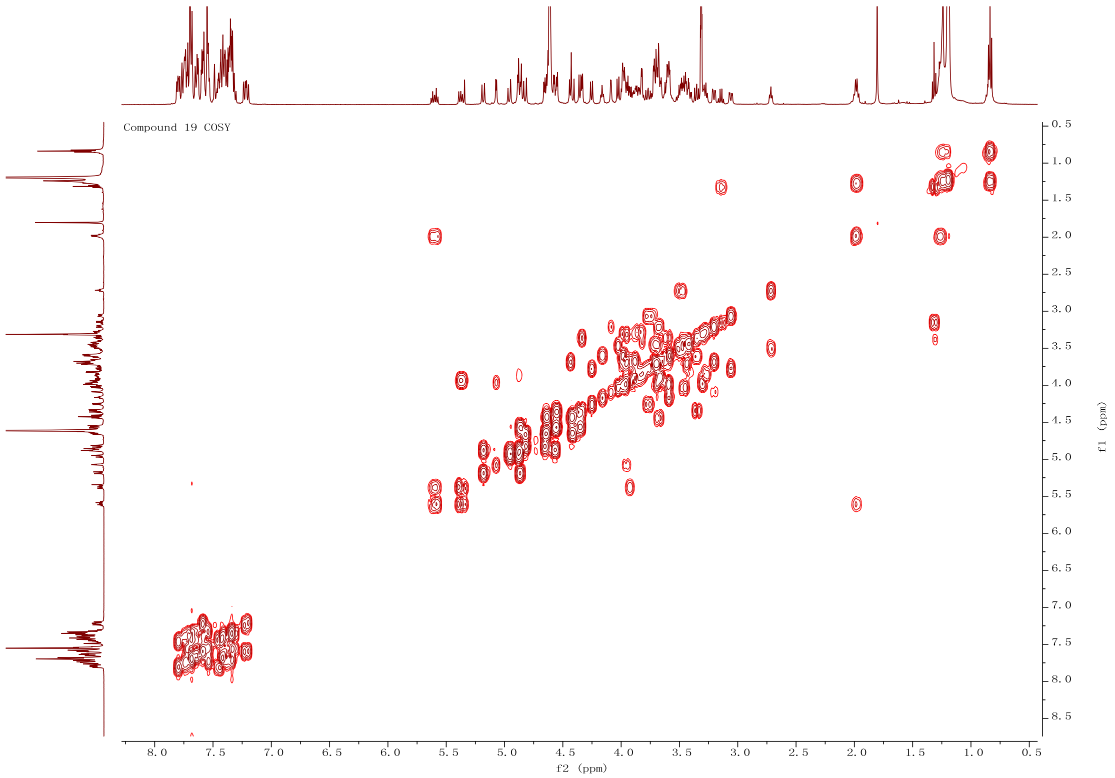
Compound 19 COSY
f2 (ppm)
f1 (ppm)

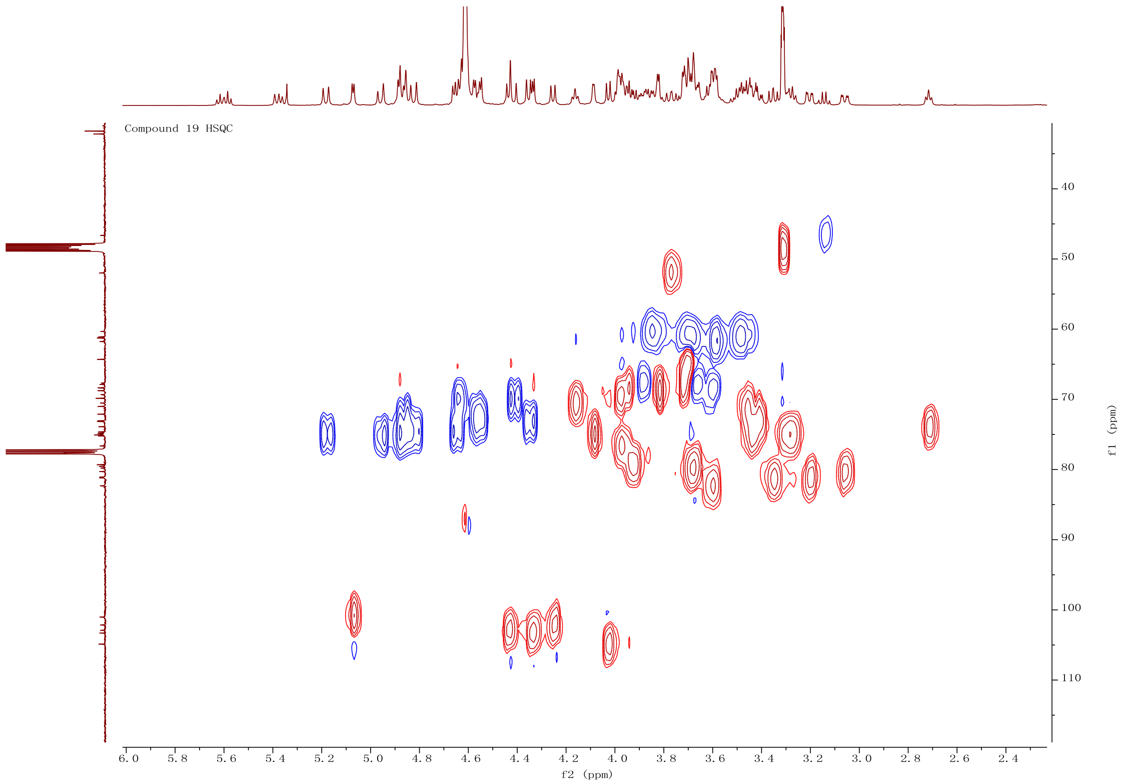
Compound 19 HSQC
f2 (ppm)
f1 (ppm)

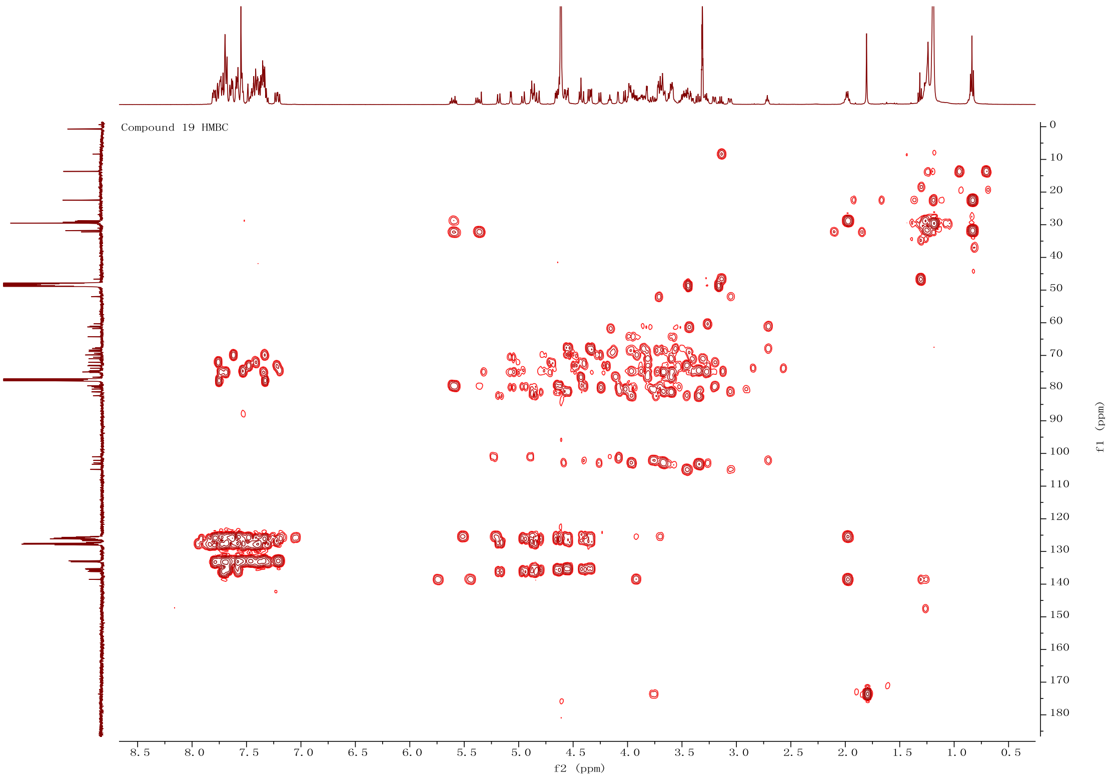

^{1}H NMR (800 MHz, CD_3OD)

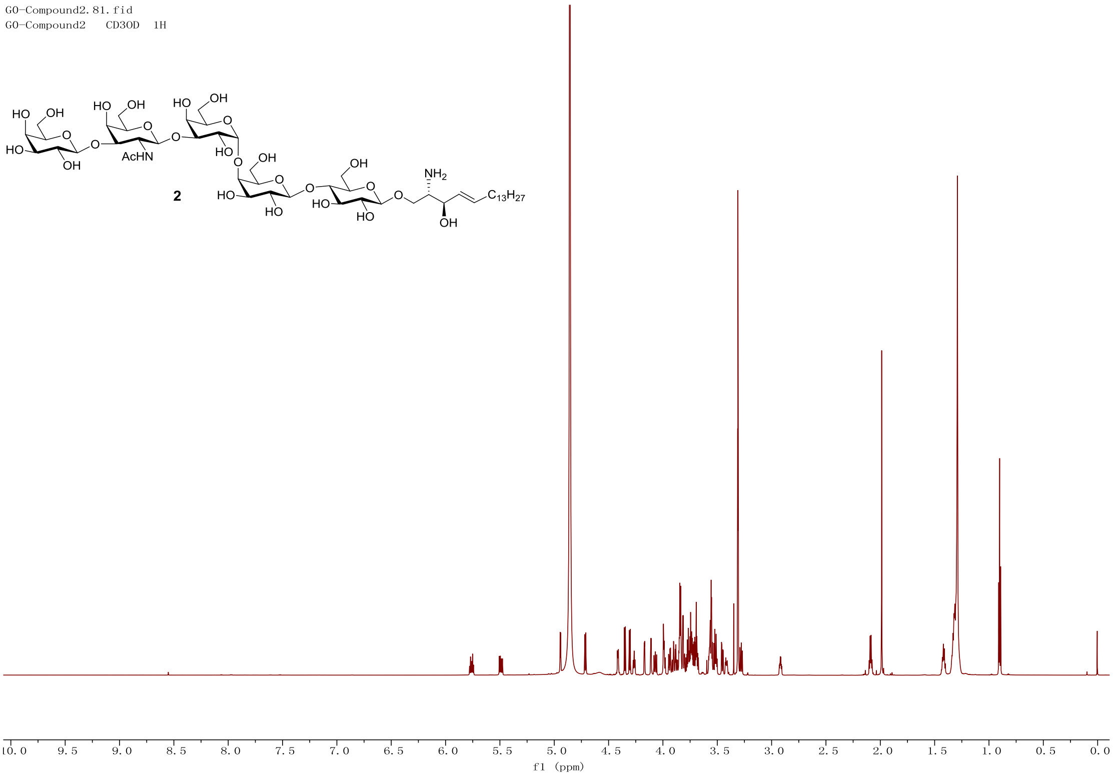

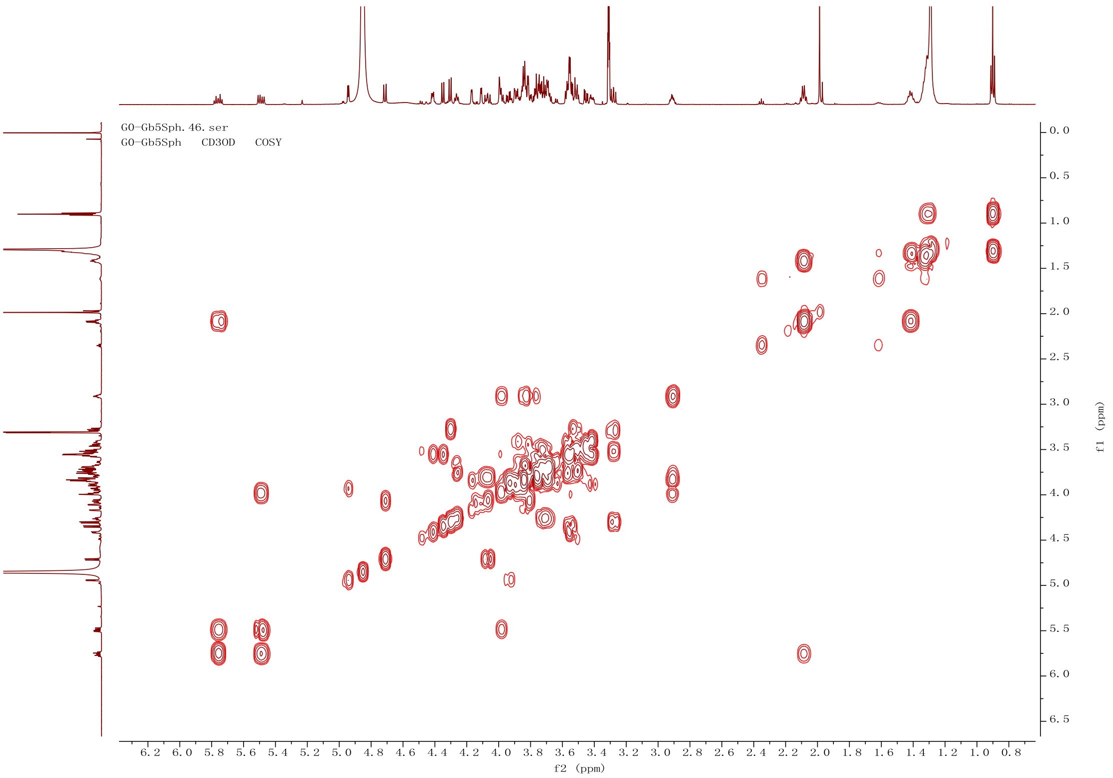
GO-Gb5Sph.46.ser
GO-Gb5Sph CD3OD COSY
f2 (ppm)
f1 (ppm)

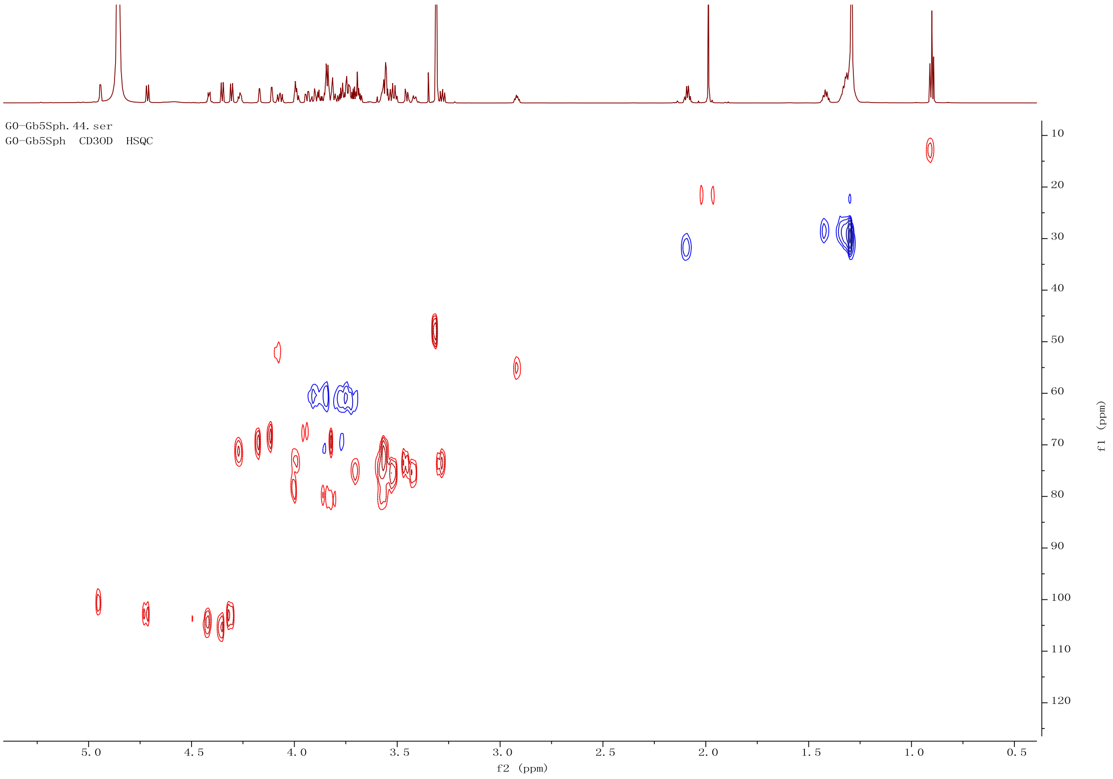
GO-Gb5Sph.44.ser
GO-Gb5Sph CD3OD HSQC
f2 (ppm)
f1 (ppm)

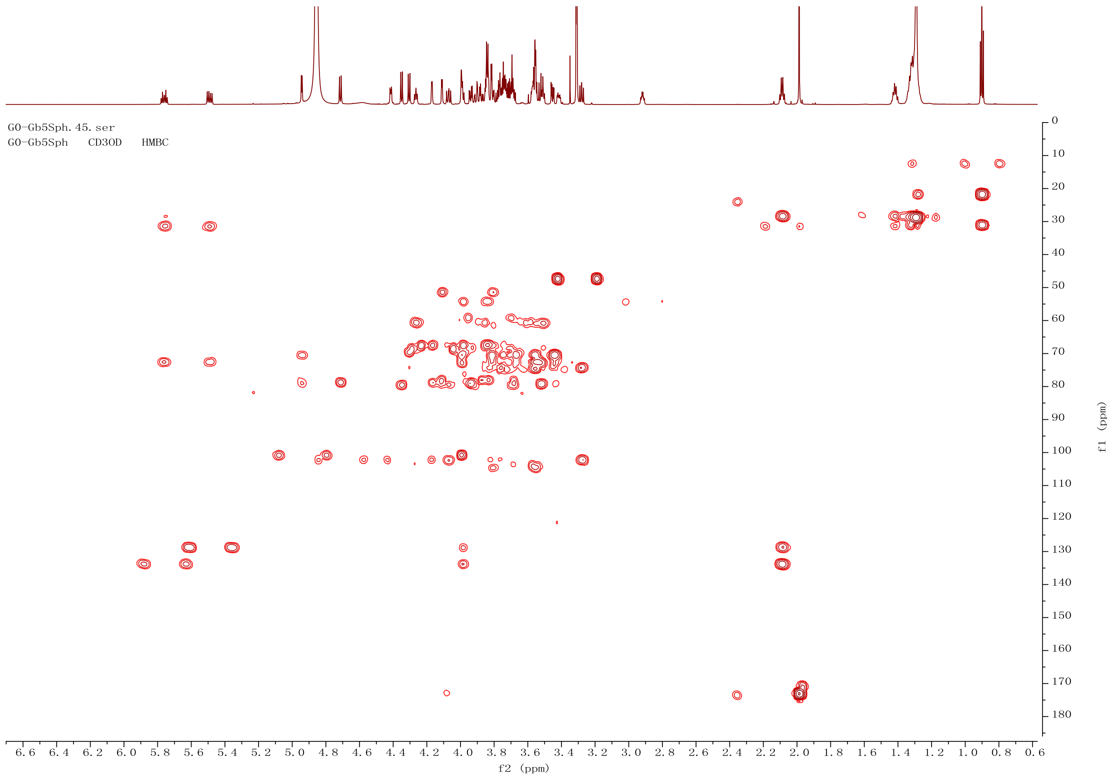

^{1}H NMR (600 MHz, CD_3OD)

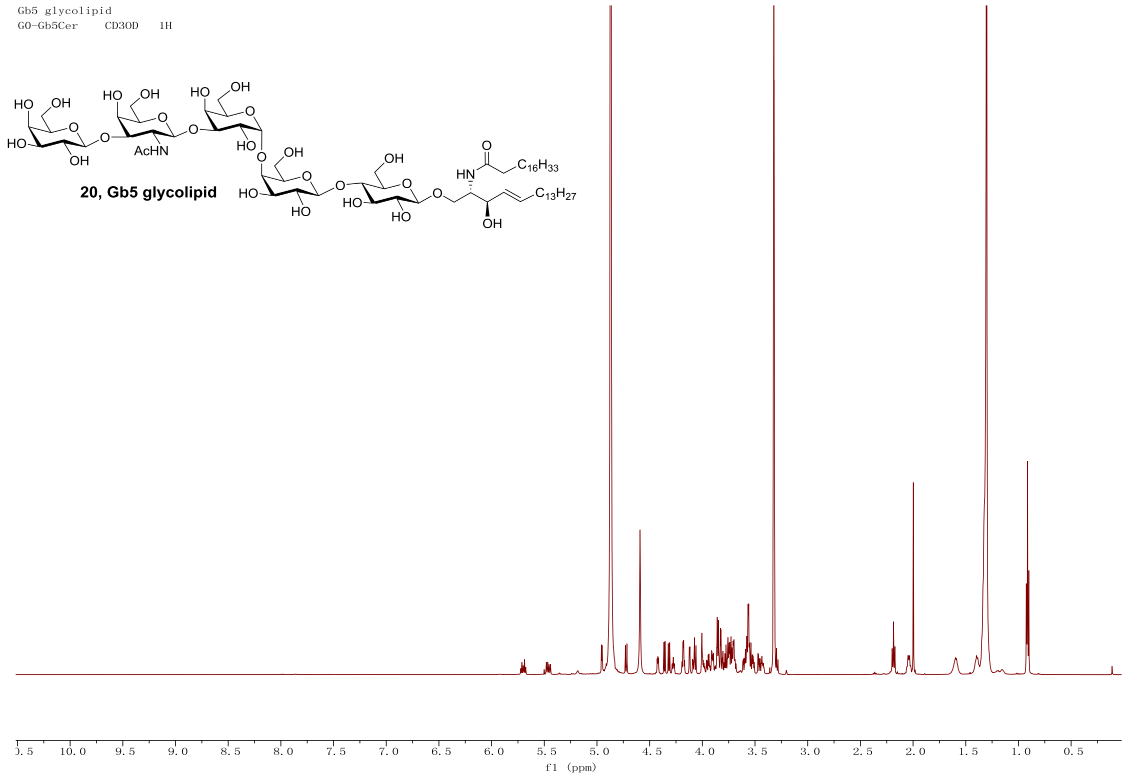

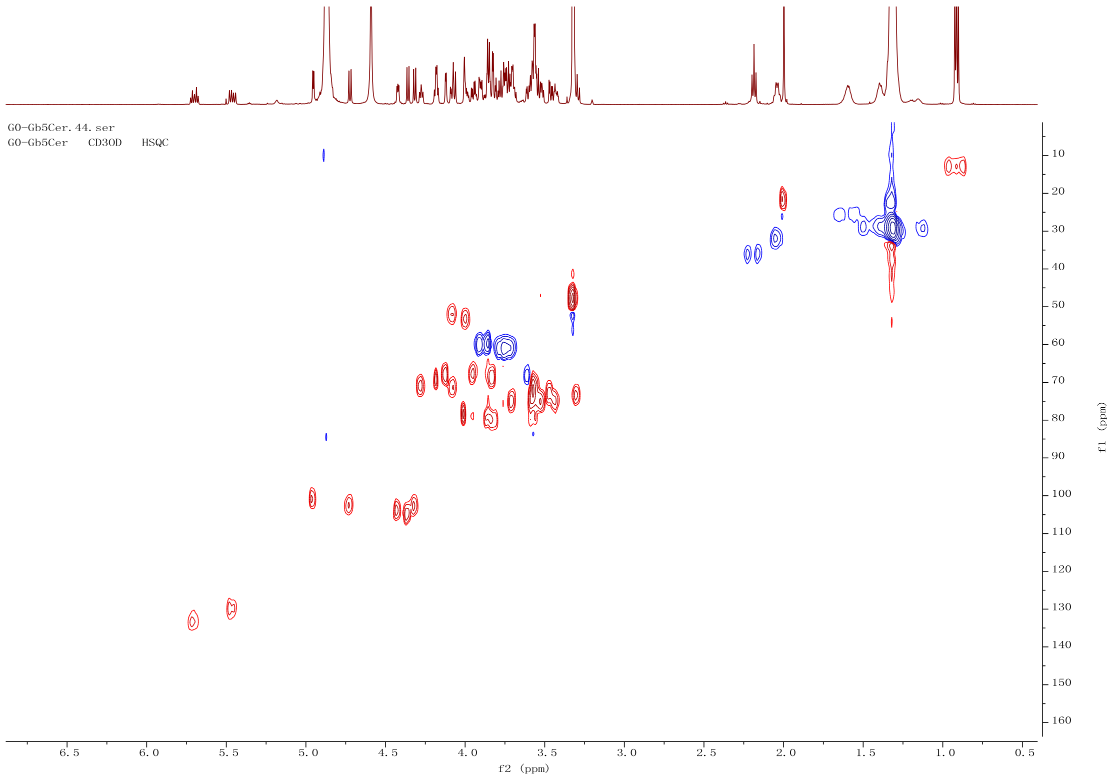

GO-Gb5Cer.44.ser
GO-Gb5Cer CD3OD HSQC
f2 (ppm)
f1 (ppm)

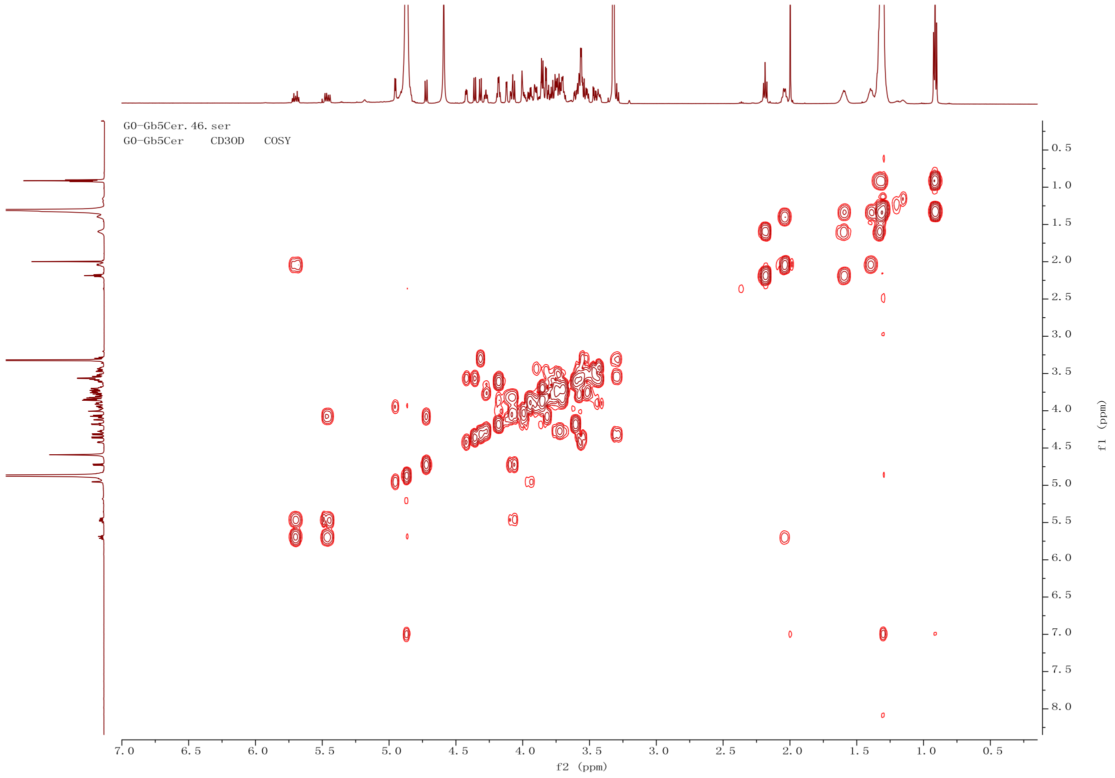

GO-Gb5Cer.46.ser
GO-Gb5Cer CD3OD COSY
f2 (ppm)
f1 (ppm)

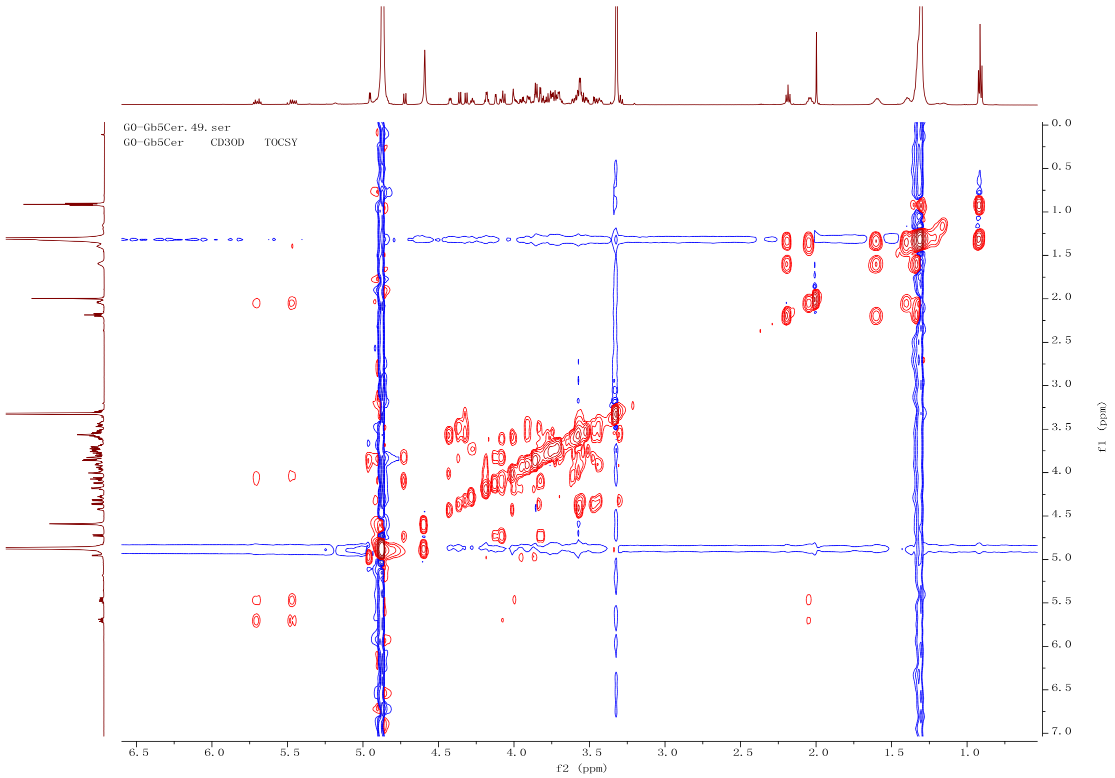

^{1}H NMR (800 MHz, CD_3OD)

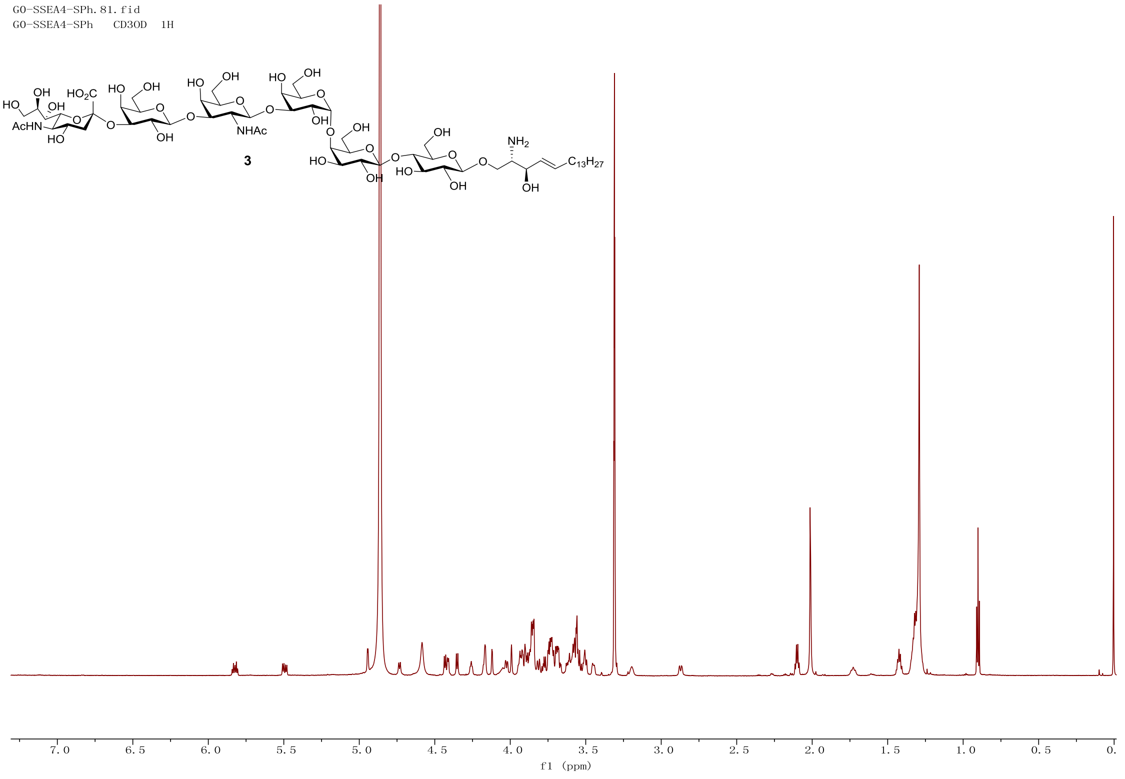

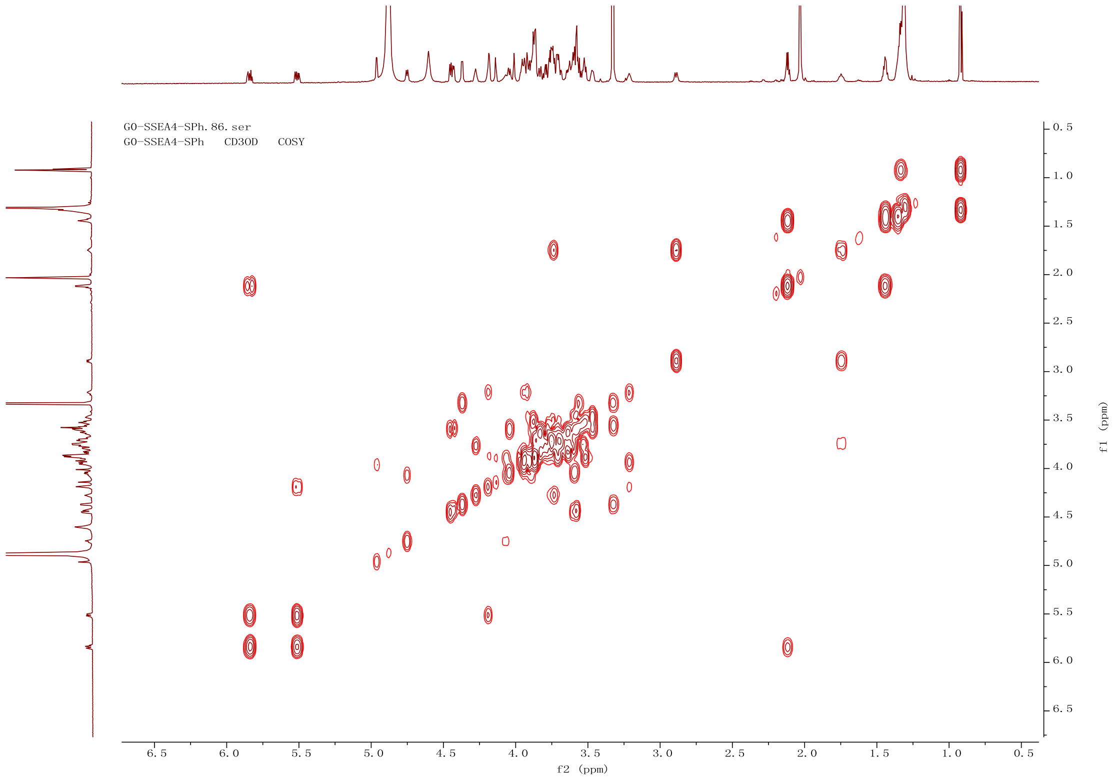
GO-SSEA4-SPh.86.ser
GO-SSEA4-SPh CD3OD COSY
f2 (ppm)
f1 (ppm)

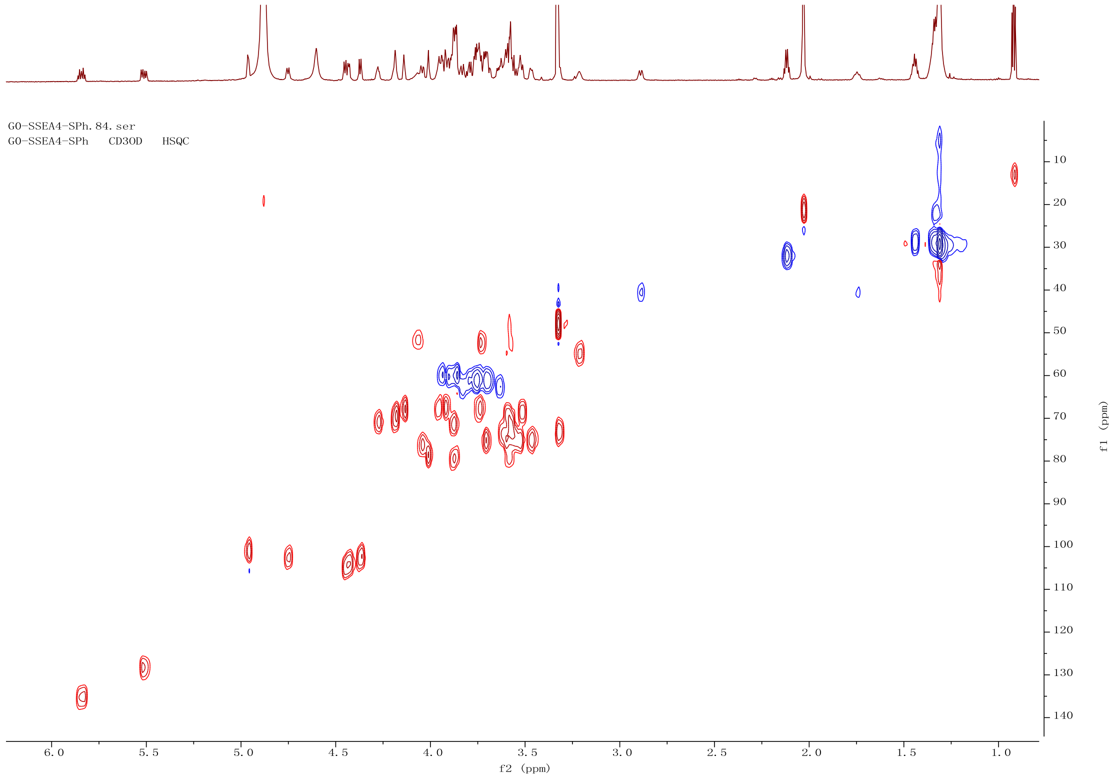
GO-SSEA4-SPh.84.ser
GO-SSEA4-SPh CD3OD HSQC
f2 (ppm)
f1 (ppm)

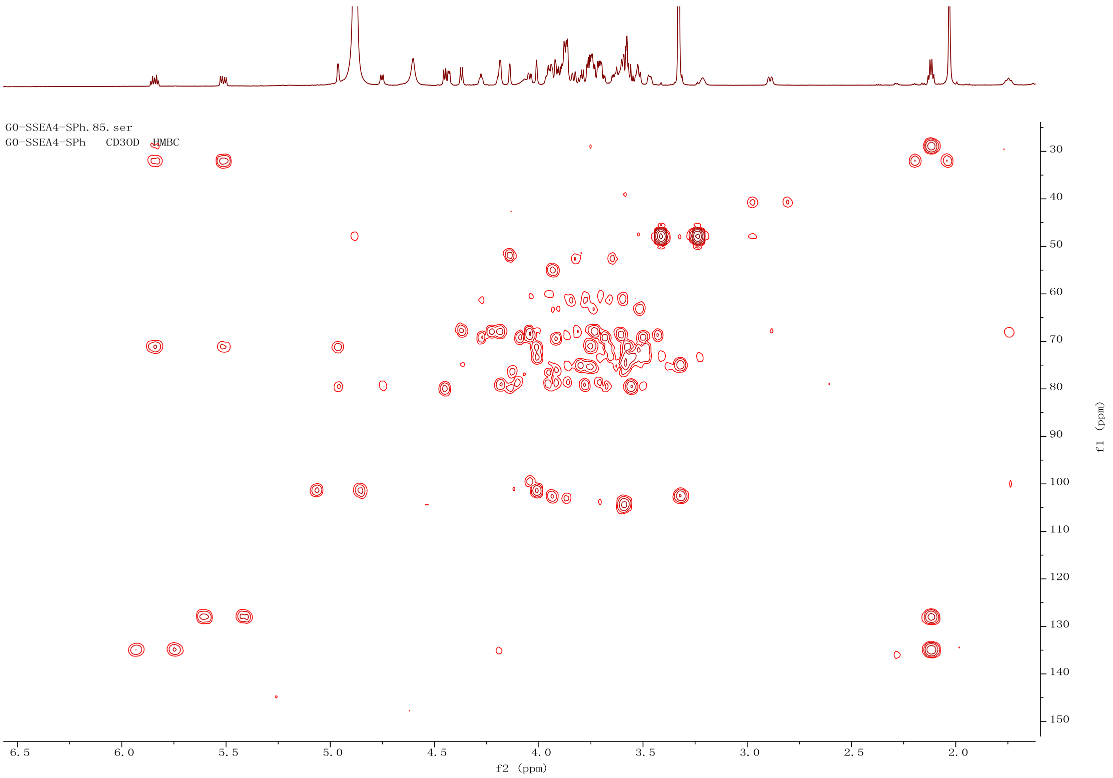

^{1}H NMR (500 MHz, CD_3OD)

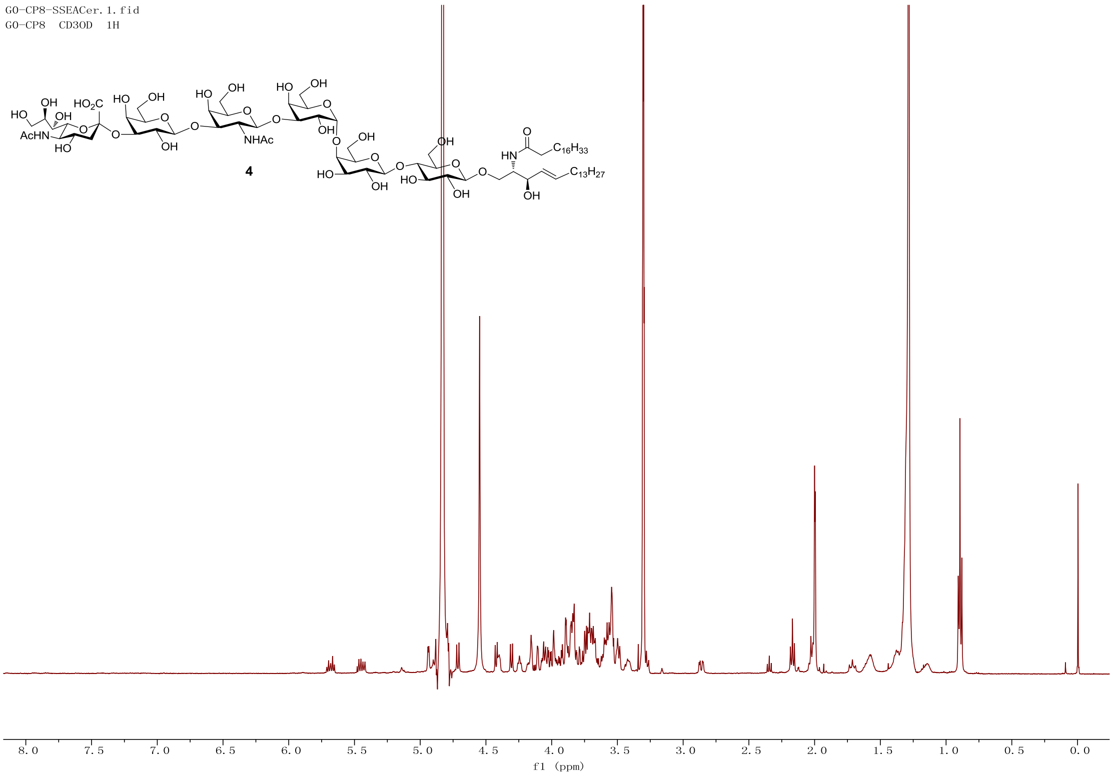

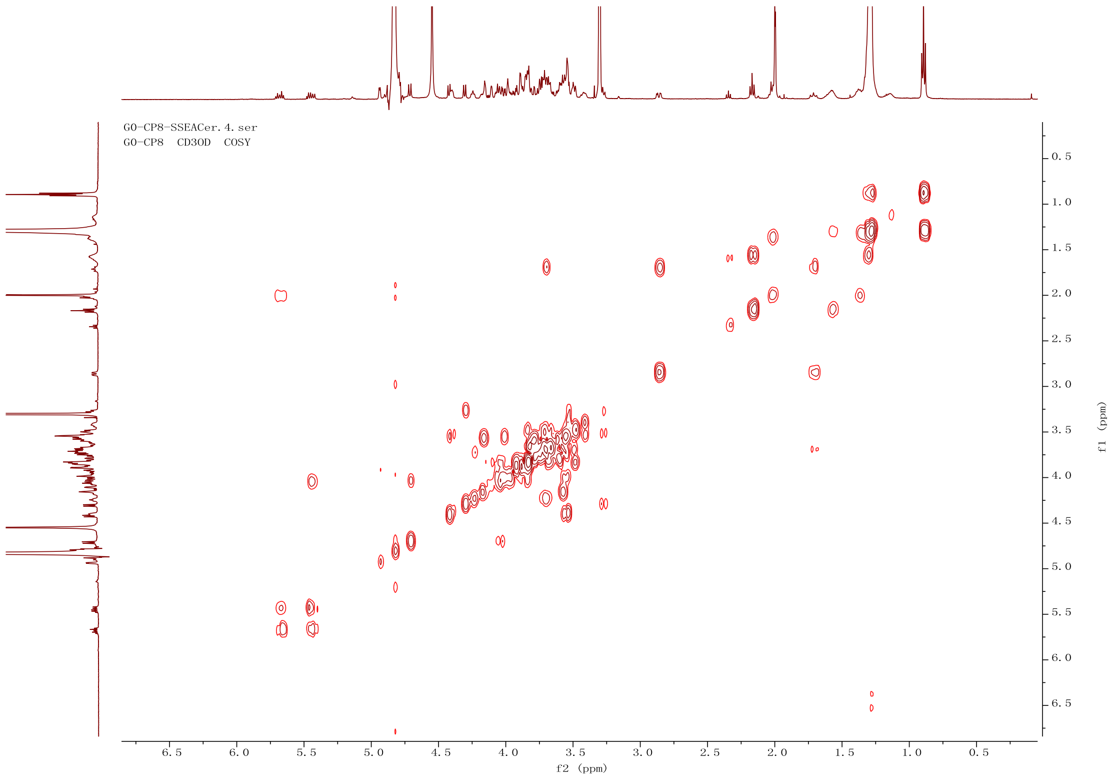
GO-CP8-SSEACer.4.ser
GO-CP8 CD3OD COSY
f2 (ppm)
f1 (ppm)

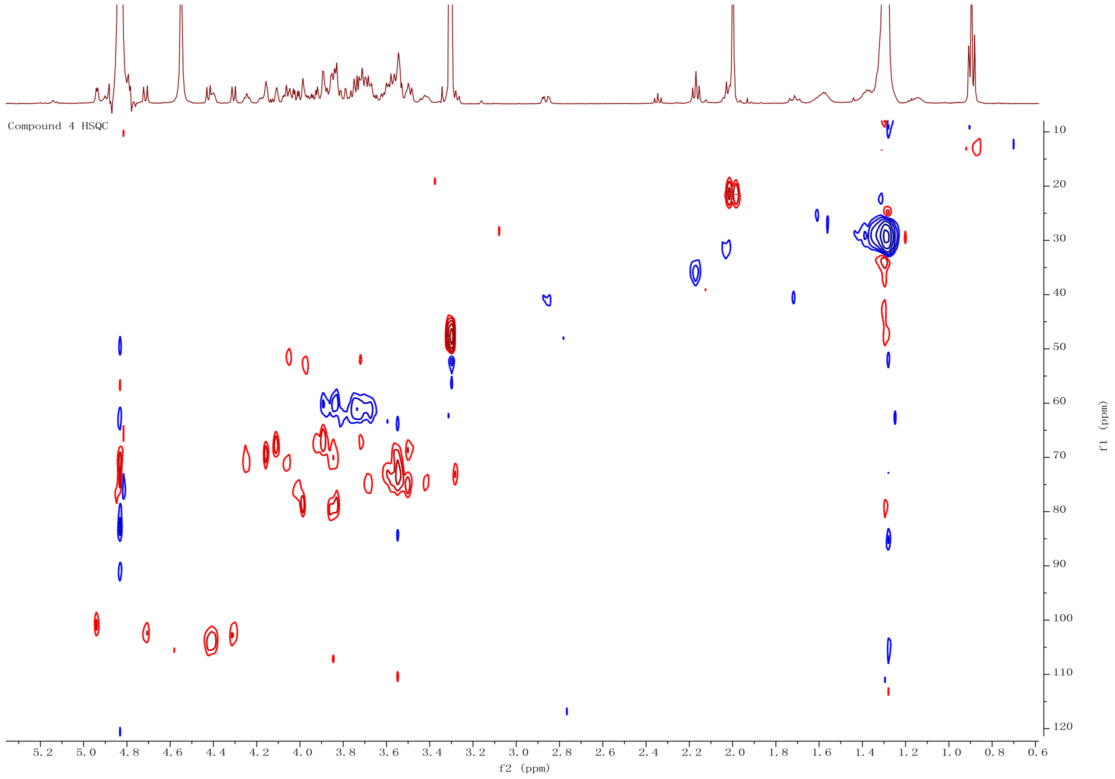
Compound 4 HSQC
f2 (ppm)
f1 (ppm)

^{1}H NMR (600 MHz, CD_3OD)

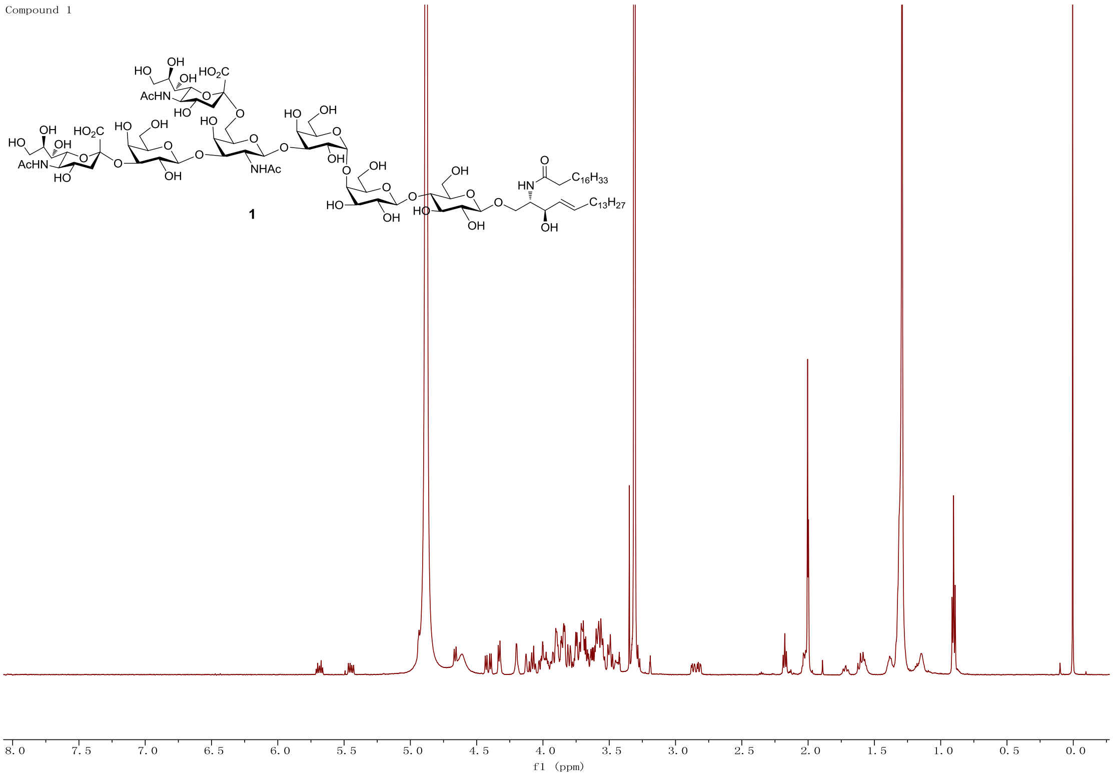

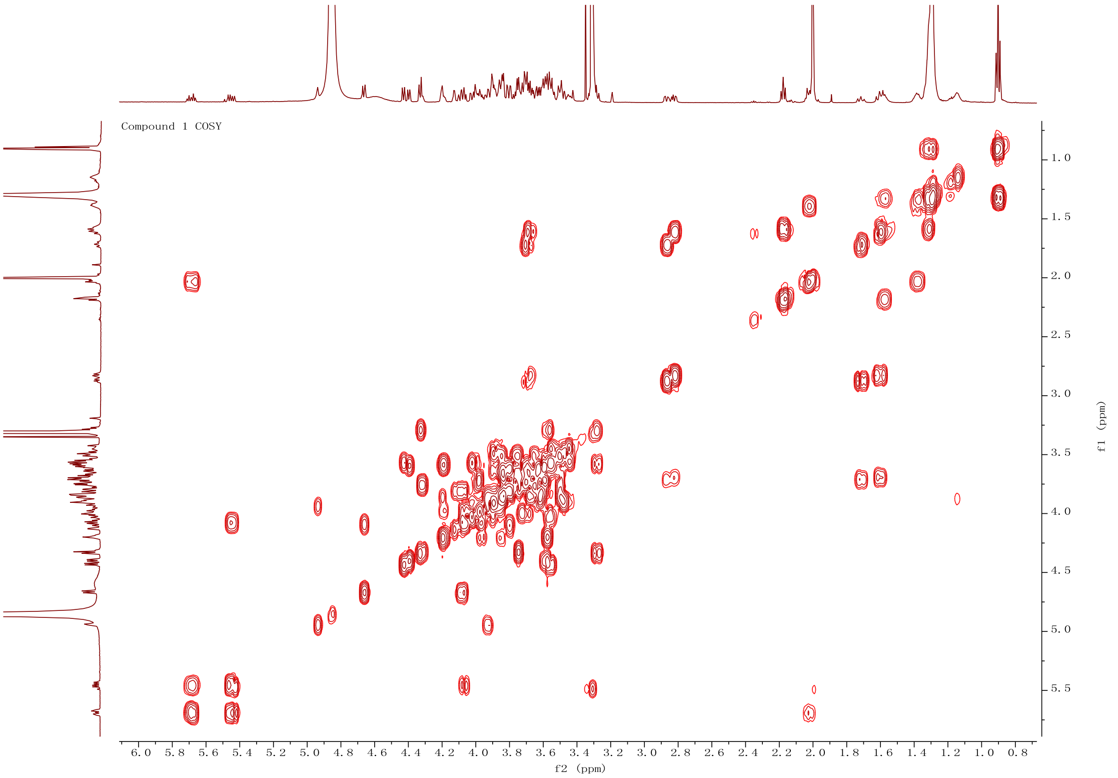

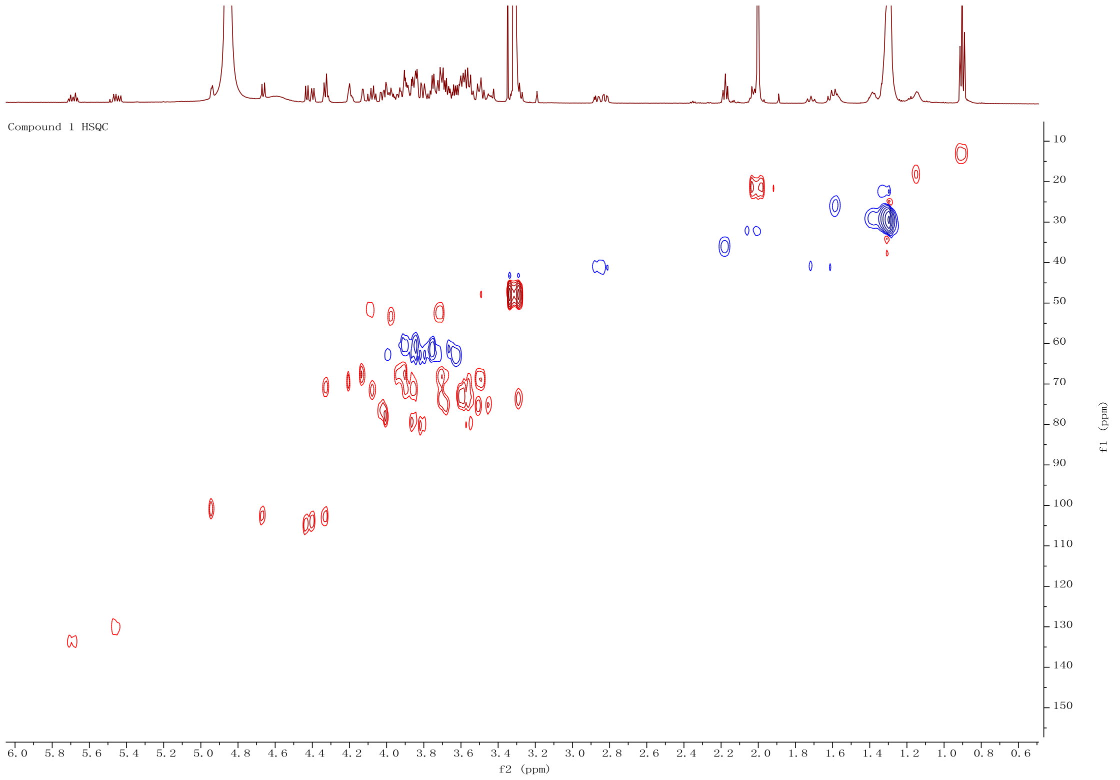

^{1}H NMR (600 MHz, D_2O)

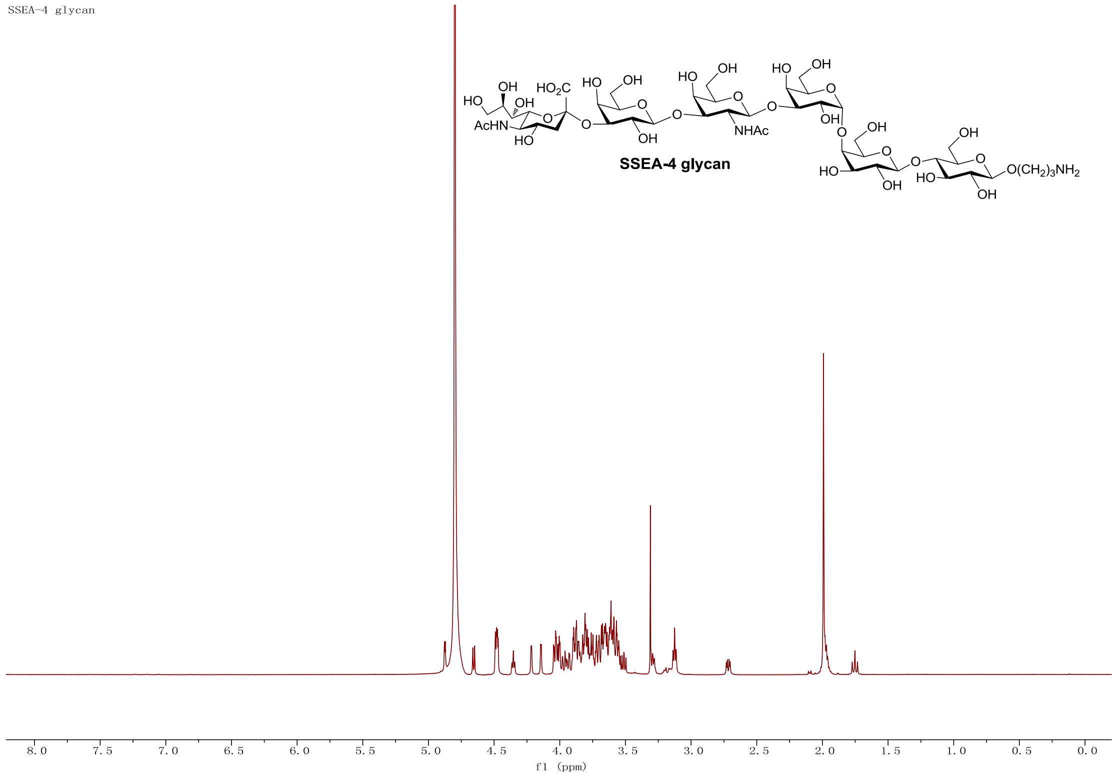

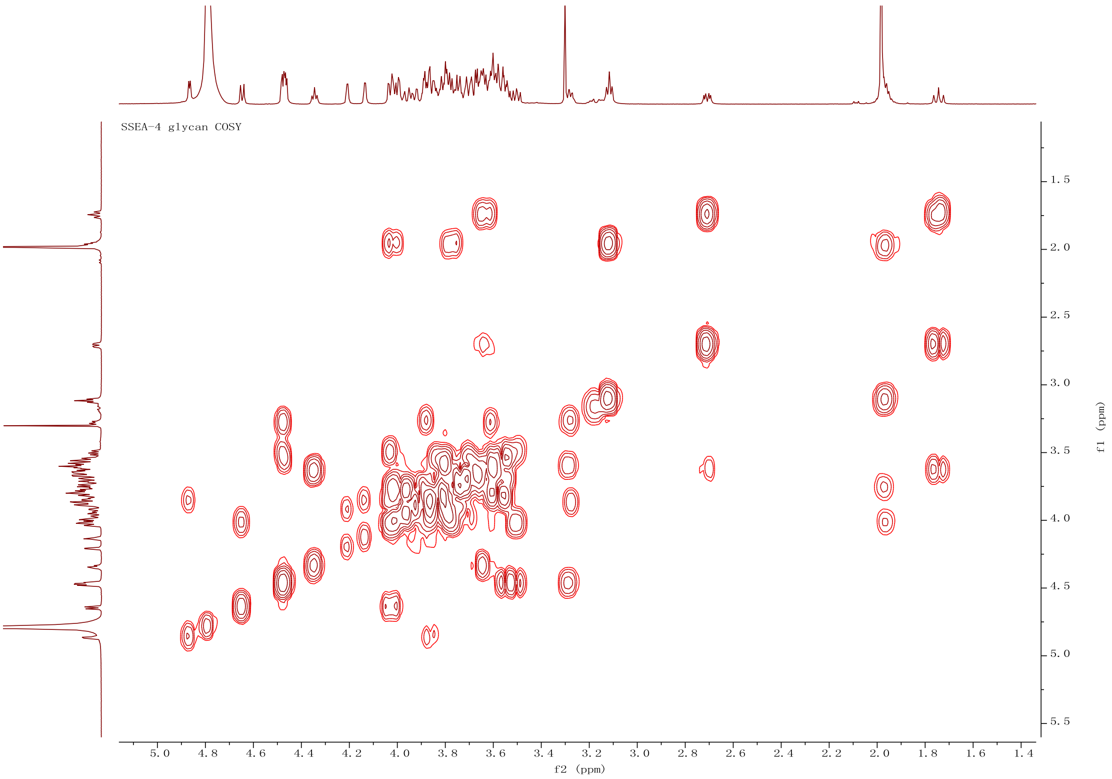
SSEA-4 glycan COSY
f2 (ppm)
f1 (ppm)

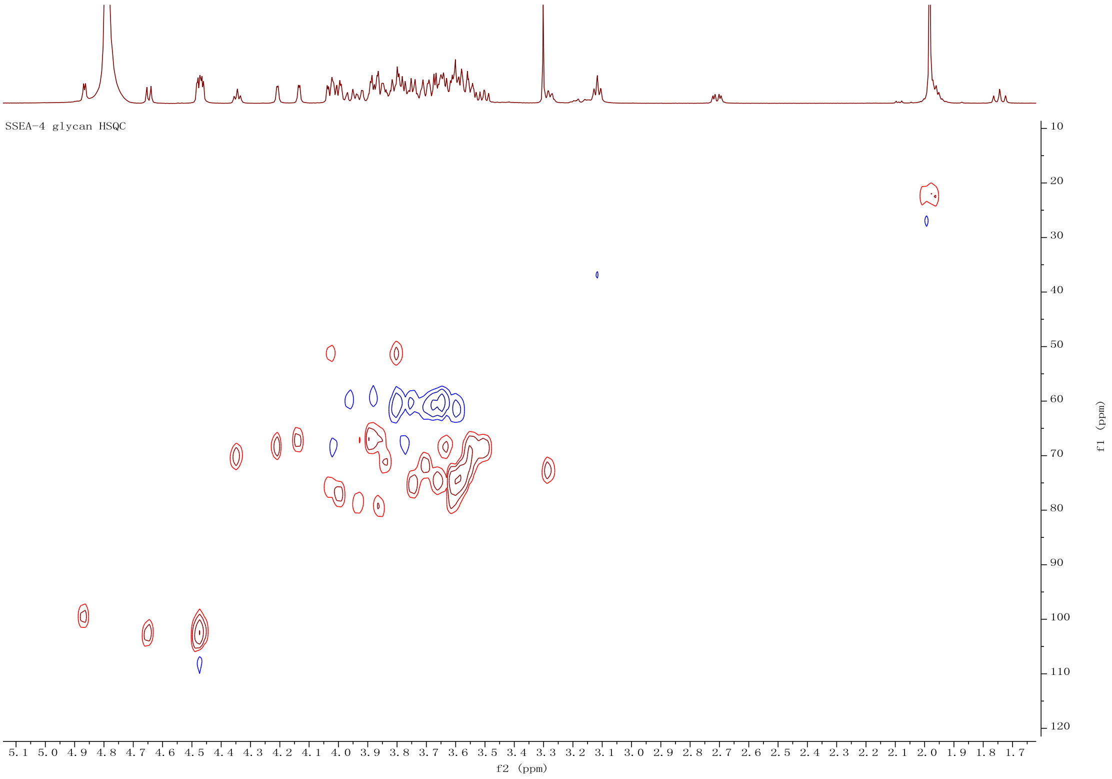
SSEA-4 glycan HSQC
f2 (ppm)
f1 (ppm)

^{1}H NMR (600 MHz, D_2O)

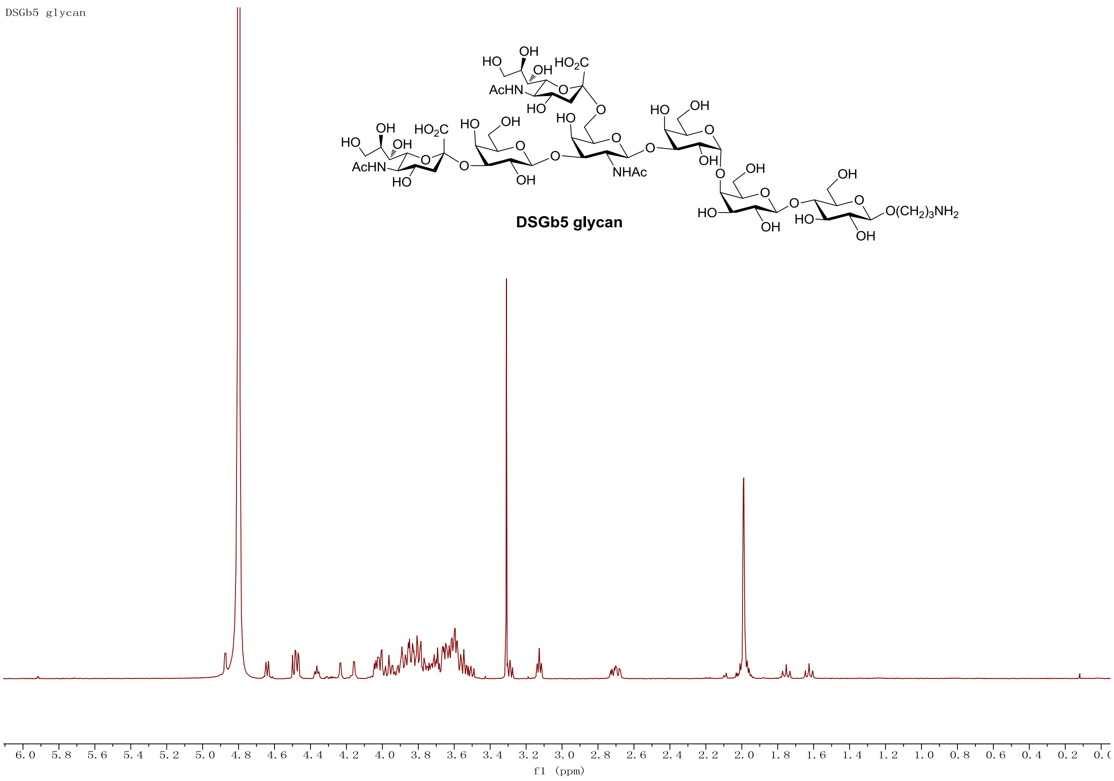

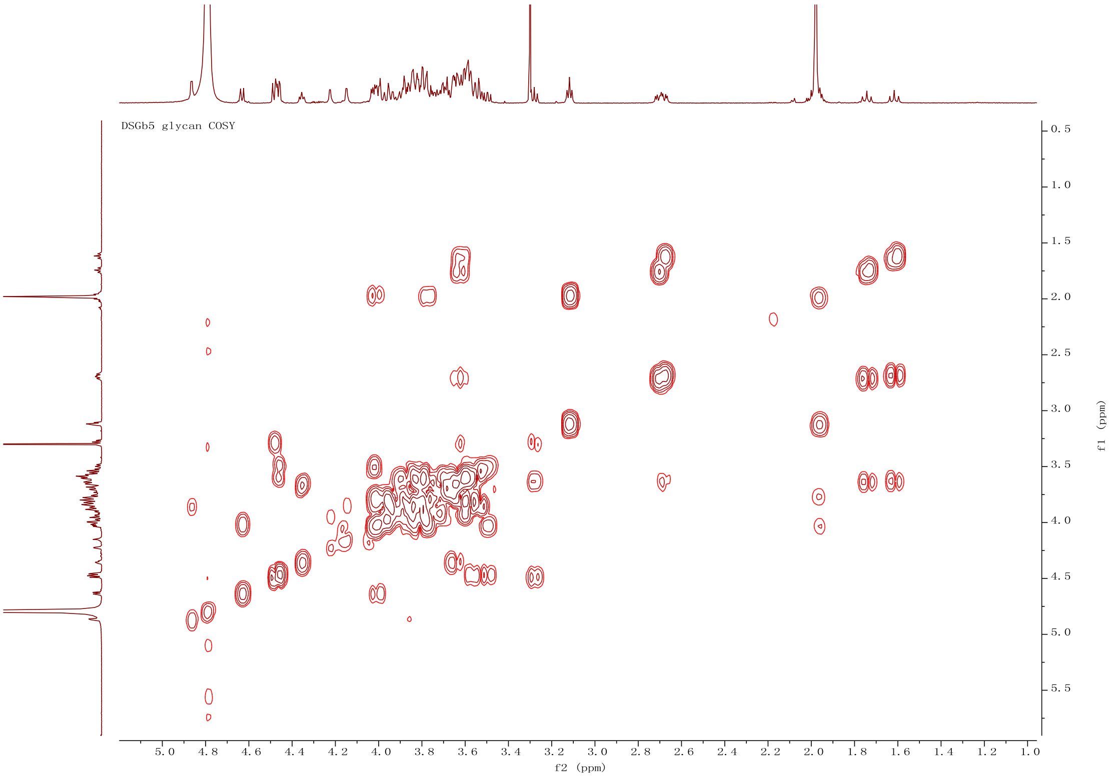

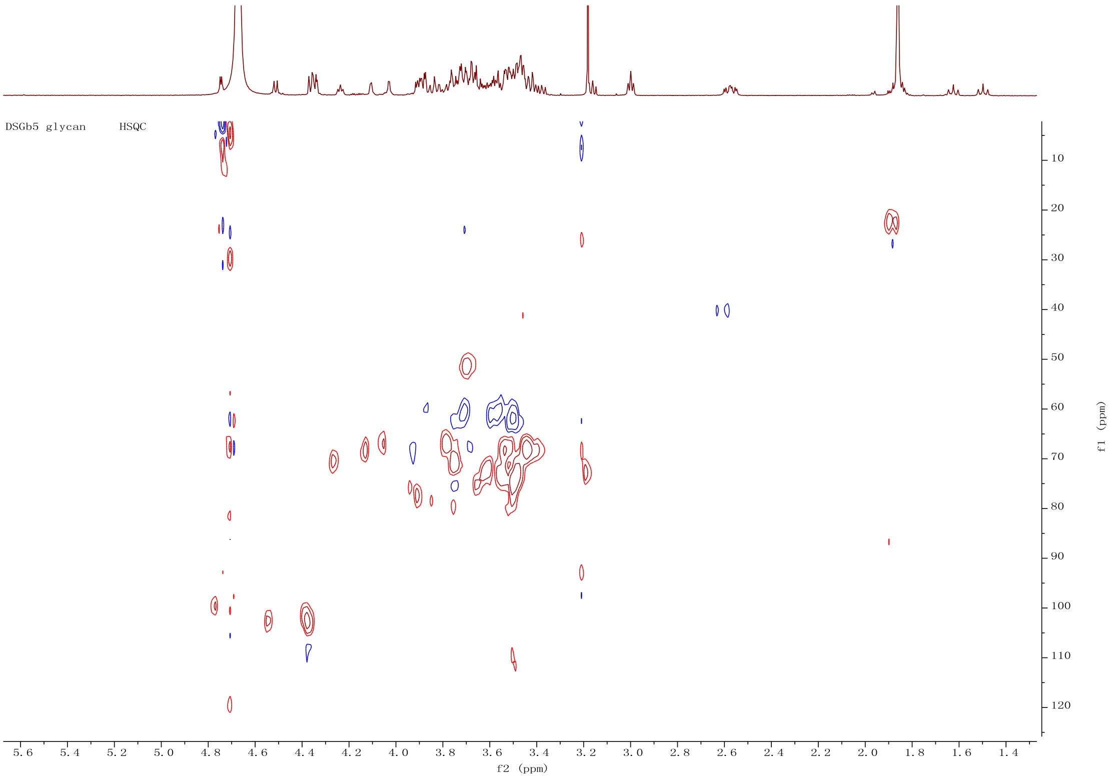

Qualitative Analysis Report

**Data Filename**	ESIH202301040-1.d	**Sample Name**	G0-G0-DSGb5Cer
**Sample ID**		**Position**	P1-C8
**Instrument Name**	Agilent G6520 Q-TOF	**Acq Method**	20160324_MS_ESIH_NEG_1min.m
**Acquired Time**	2/17/2023 15:20:26	**IRM Calibration Status**	Success
**DA Method**	small molecular data analysis method.m	**Comment**	ESIH by fangsu

User Spectra

Fragmentor Voltage	Collision Energy	Ionization Mode
170	0	ESI

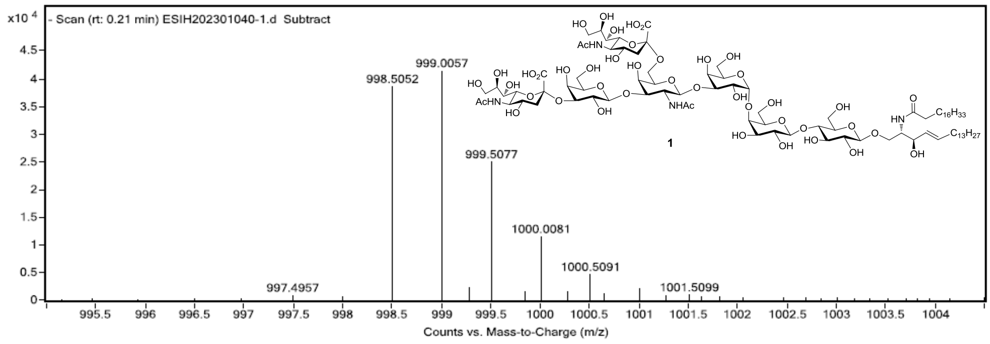

Formula Calculator Results

m/z	Calc m/z	Diff (mDa)	Diff (ppm)	Ion Formula	Ion
998.5052	998.5052	0.01	0.01	C90 H156 N4 O44	(M-2H)-2

--- End Of Report ---

Qualitative Analysis Report

**Data Filename**	ESIH202204917.d	**Sample Name**	G0-G0-DSGb5-NH2
**Sample ID**		**Position**	P1-B2
**Instrument Name**	Agilent G6520 Q-TOF	**Acq Method**	20160322_MS_ESIH_POS_1min.m
**Acquired Time**	11/22/2022 14:34:32	**IRM Calibration Status**	Success
**DA Method**	small molecular data analysis method.m	**Comment**	ESIH by fangsu

User Spectra

Fragmentor Voltage	Collision Energy	Ionization Mode
175	0	ESI

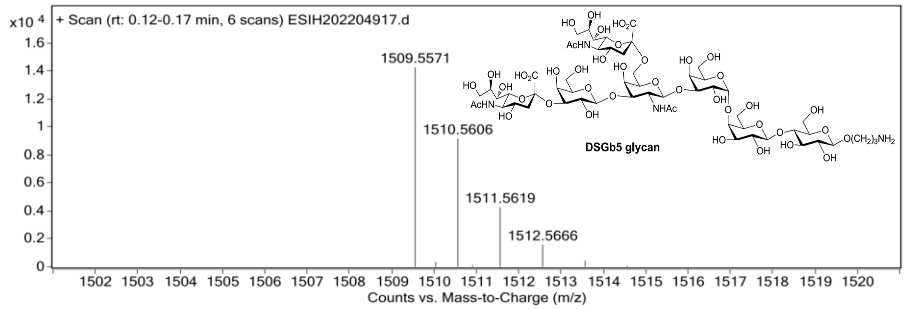

Formula Calculator Results

m/z	Calc m/z	Diff (mDa)	Diff (ppm)	Ion Formula	Ion
1509.5571	1509.5572	0.05	0.03	C57 H97 N4 O42	(M+H)+

--- End Of Report ---
